# ‘Reverse biomimetic’ synthesis of l-arogenate and its stabilized analogues from l-tyrosine†

**DOI:** 10.1039/d1sc03554a

**Published:** 2021-07-30

**Authors:** Louise Eagling, Daniel J. Leonard, Maria Schwarz, Iñaki Urruzuno, Grace Boden, J. Steven Wailes, John W. Ward, Jonathan Clayden

**Affiliations:** School of Chemistry, University of Bristol Cantock's Close Bristol BS8 1TS UK j.clayden@bristol.ac.uk; Jealott's Hill International Research Centre Bracknell RG42 6EY UK

## Abstract

l-Arogenate (also known as l-pretyrosine) is a primary metabolite on a branch of the shikimate biosynthetic pathway to aromatic amino acids. It plays a key role in the synthesis of plant secondary metabolites including alkaloids and the phenylpropanoids that are the key to carbon fixation. Yet understanding the control of arogenate metabolism has been hampered by its extreme instability and the lack of a versatile synthetic route to arogenate and its analogues. We now report a practical synthesis of l-arogenate in seven steps from *O*-benzyl l-tyrosine methyl ester in an overall yield of 20%. The synthetic route also delivers the fungal metabolite spiroarogenate, as well as a range of stable saturated and substituted analogues of arogenate. The key step in the synthesis is a carboxylative dearomatization by intramolecular electrophilic capture of tyrosine's phenolic ring using an *N*-chloroformylimidazolidinone moiety, generating a versatile, functionalizable spirodienone intermediate.

## Abstract 2

l-Tyrosine provides a precursor for a practical synthesis of the unstable primary metabolite l-arogenate and some stabilised arogenate analogues.

## Graphical abstract

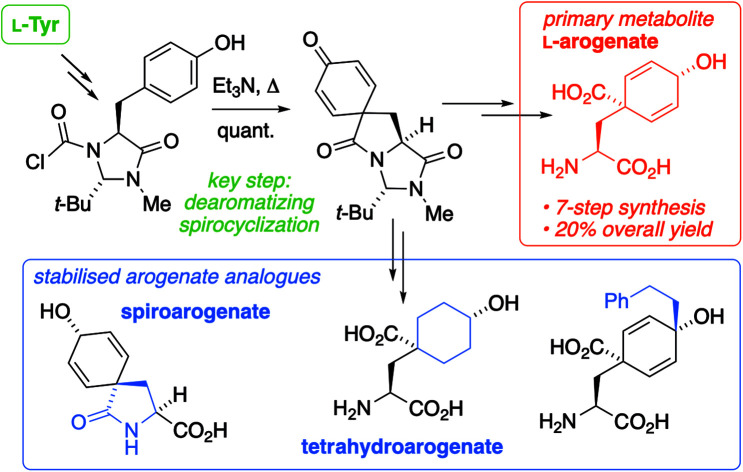

## Introduction

**Scheme 1:**
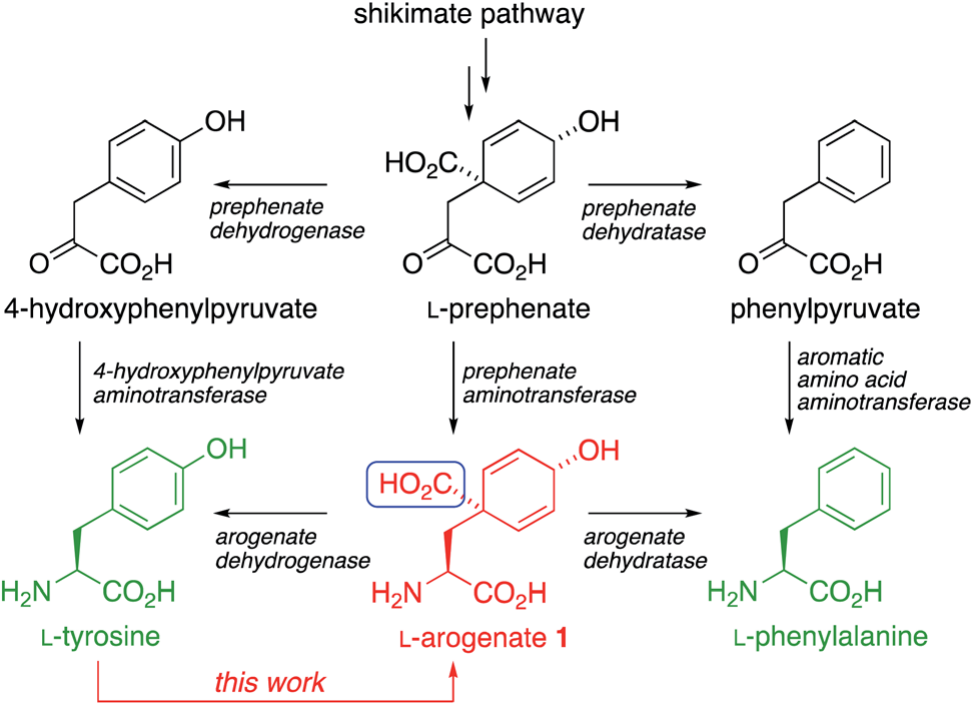
The biosynthesis of l-Tyr and l-Phe, and (in red) our ‘reverse biomimetic’ synthesis of l-arogenate **1**, introducing the highlighted carboxyl group by an intramolecular dearomatizing carboxylation.

The aromatic amino acids l-Tyr and l-Phe are biosynthesized through the shikimate pathway (Scheme 1), in which the non-aromatic precursor prephenate is reductively aminated and aromatized to yield tyrosine and phenylalanine.^1–3^ Variants of the pathway exist in which either aromatization or reductive amination occurs first. The former passes through phenylpyruvate or its 4-hydroxy derivative, while the latter (discovered by Stenmark in 1974 (ref. 3)) passes through l-arogenate, **1** (also known as pretyrosine) as the final non-aromatic intermediate.^4^ The shikimate pathway is present in plants (where it may process >30% of photosynthetically fixed carbon), bacteria, fungi, and some other organisms, but is absent from animals, which must obtain aromatic amino acids from their diet. The pathway is therefore an attractive target for development of new herbicides, antimicrobial agents, and live vaccines.^5^

Two routes to l-arogenate **1** have been reported previously. In one,^6^ the key spirocyclic cyclohexadienol intermediate was formed from a non-biogenetic precursors by Diels–Alder cycloaddition. Conversion of the adduct into l-arogenate was confirmed by NMR, but the final product was not isolated. In the other,^7^ Birch reduction and Michael addition gave a dearomatised intermediate that was resolved and converted into a sample of arogenate. We now report the first asymmetric synthesis of an isolated sample of l-arogenate by a direct and practical route, using arogenate's biosynthetic product l-tyrosine as a starting material. We characterise this strategy as a ‘reverse biomimetic’ synthesis. The synthesis is rapid, efficient, scalable, and highly amenable to the synthesis of more stable arogenate analogues.

Our synthetic route employs the dearomatizing cyclization^8^ of tyrosine's phenolic ring as an atom-economic way to install the spirocyclohexadienol ring of the target. Dearomatising cyclisations – whether by nucleophilic, electrophilic, or radical methods – allow stereochemical complexity to be built from a simple planar precursor, and have been used in the syntheses of a number of natural products.^9^ Dearomatizing spirocyclisations of phenols have been widely exploited, yet remarkably few of these employ tyrosine, and all of them are oxidative. These examples produced spirolactones,^10^ spirolactams^11^ and a spirodienone carboxylic acid precursor to the natural product discorhabdin.^12^

## Results and discussion

**Scheme 2:**
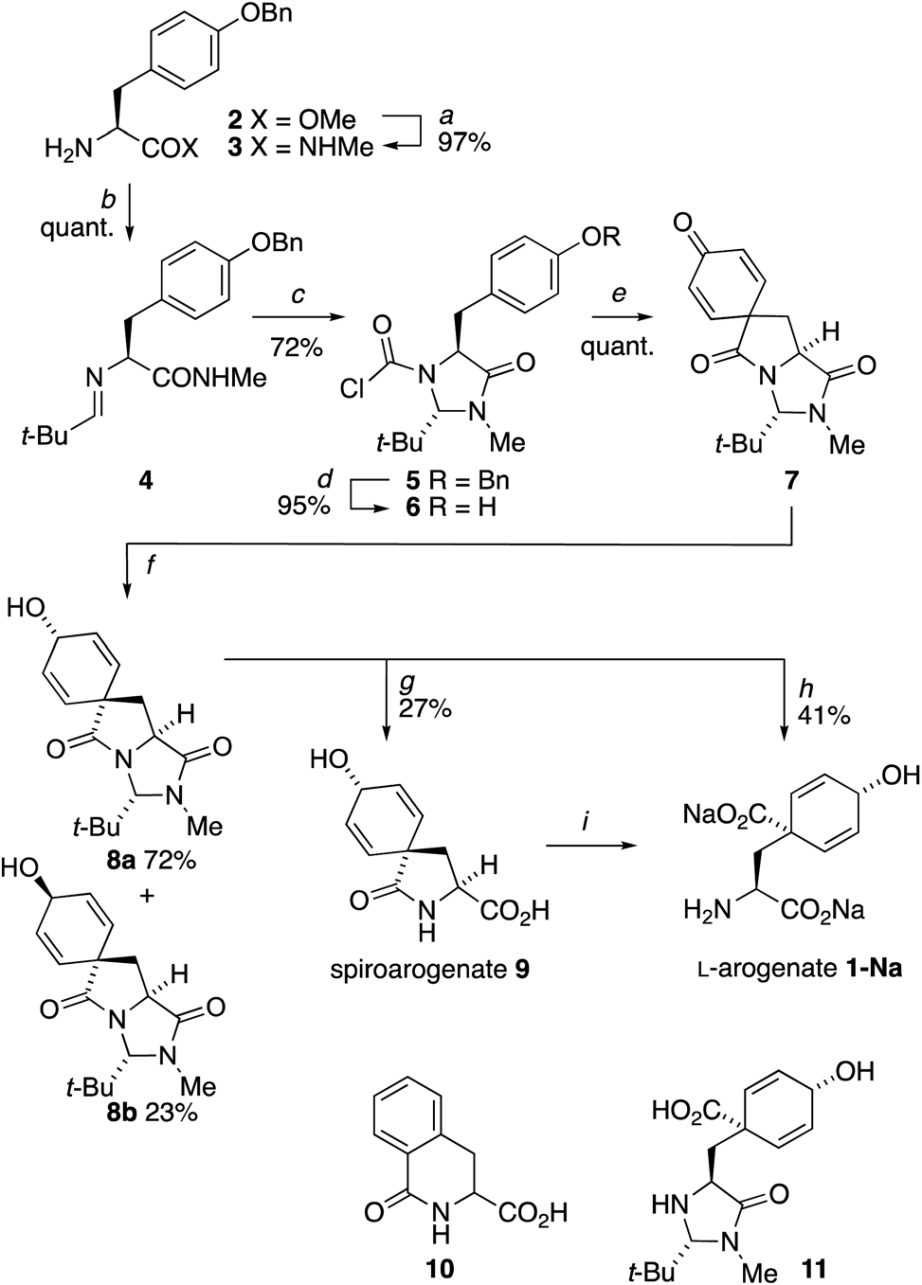
Synthesis of l-arogenate **1** by dearomatizing cyclization of the *N*-chloroformylimidazolidinone derivative **6** of l-Tyr. Reagents and conditions: (a) MeNH_2_, 24 h; (b) *t*-BuCHO, MgSO_4_, 16 h; (c) COCl_2_, pyridine, THF, rt, 3h; (d) H_2_, Pd/C, THF, rt, 4 h; (e) Et_3_N, MeCN, 150 °C (mw), 10 min; (f) NaBH_4_, CeCl_3_·7H_2_O, MeOH, −78 °C, 30 min; (g) Ba(OH)_2_, H_2_O, 30 °C, 18 h; (h) Ba(OH)_2_, BaCO_3_, H_2_O, 80 °C, 5 h; (i) Reported conditions: NaOH, Na_2_CO_3_, EtOH, 70 °C, 20 h.^6^

The biosynthetic decarboxylative aromatization that converts arogenate to tyrosine – a reaction we sought to run in reverse – involves the formal elimination of a one-carbon unit (boxed in blue in Scheme 1) at the formic acid oxidation level. In the course of an exploration of a Bischler–Napieralski type cyclization of some carbamoyl chloride derivatives of aromatic amino acids,^13^ which typically gives isoquinolinones without dearomatisation, we discovered reactivity unique to l-tyrosine. We found that tyrosine's imidazolidinone derivative **6** (Scheme 2) underwent a dearomatizing carbonylation that allowed this one-carbon unit could be reintroduced exactly as required for a possible synthesis of arogenate.

**Fig. 1:**
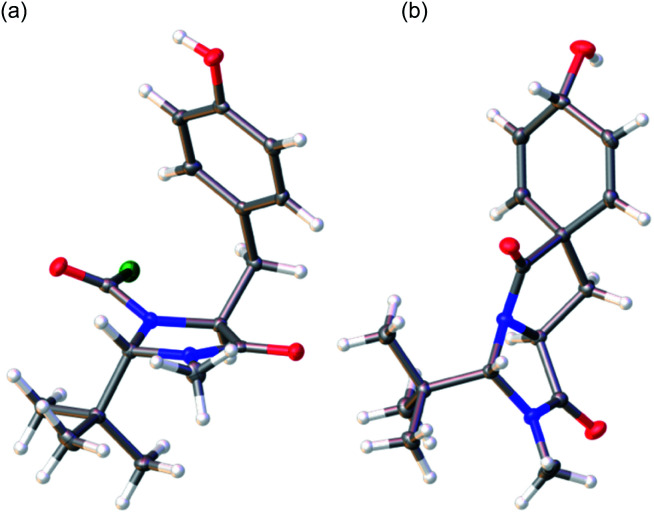
(a) X-ray crystal structure of **6**; (b) X-ray crystal structure of minor diastereoisomer **8b**.^21^

The *trans* diastereoisomer of carbamoyl chloride **5** was synthesised selectively from *O*-benzyl l-tyrosine methyl ester **2** by formation of the methyl amide **3**, condensation with pivalaldehyde, and phosgene-induced cyclization of the imine **4** under kinetic control.^14,15^ Heating benzyl ether **5** induces cyclization to an isoquinolinone ring,^13^ but quite different reactivity is observed with the free phenol **6** (X-ray crystal structure, confirming relative stereochemistry, shown in Fig. 1a) that was formed cleanly by hydrogenolysis of **5** in THF. These conditions preserved the (surprisingly stable^15,16^) carbamoyl chloride group.^17^

We found that heating **6** to 60 °C for 72 h in the presence of Et_3_N and KI induced dearomatizing spirocyclisation to the dienone **7**, a compound with clear structural homology to arogenate **1**, in 82% yield. Optimization of this cyclization (see ESI†) showed that microwave irradiation with Et_3_N in acetonitrile for 10 min at 150 °C gave **7** cleanly in quantitative yield from **6** without the need for chromatographic purification. Spirocycle **7** was fully bench stable over periods of months, but exposure to acid or base (including attempts to hydrolyse its lactam rings) led to decomposition products arising principally from rearomatization.

This spirocyclization contrasts with related electrophilic intramolecular acylations of non-phenolic compounds, which yield isoquinolinones^13^ without dearomatisation. There are no other reported acylative dearomatisations of phenols.^18^

To generate the dienol structure characteristic of arogenate, the spirocyclic dienone **7** was reduced cleanly under Luche conditions^19^ at −78 °C to a separable 3.1 : 1 mixture of diastereoisomeric alcohols **8a** and **8b**, from which **8a** was isolated in 72% yield. The minor diastereoisomer **8b** was crystalline, and an X-ray crystal structure (Fig. 1b) allowed us to assign the relative configurations of **8a** and **8b**. In the major product **8a**, formed by approach of the reducing agent *anti* to the more hindered carbonyl and *tert*-butyl group, the hydroxyl group is orientated *syn* to the carbonyl group, as is also the case in arogenate and prephenate.

Methods were sought for the conversion of **8a** to arogenate **1** by hydrolysis of the imidazolidinone and lactam rings. Heating with acid led to rearomatization to products that included dihydroisoquinolone **10** and phenylalanine. Likewise, most bases promoted rearomatization to mixtures of phenylalanine- and tyrosine-derived products. Barium hydroxide gave the most encouraging results: treatment with 12 equiv. aqueous Ba(OH)_2_ for 18 h at 30 °C opened the imidazolidinone ring to yield a sample containing >50% by ^1^H NMR of the partially hydrolysed lactam **9**, which is the natural product spiroarogenate.^20^

**Fig. 2:**
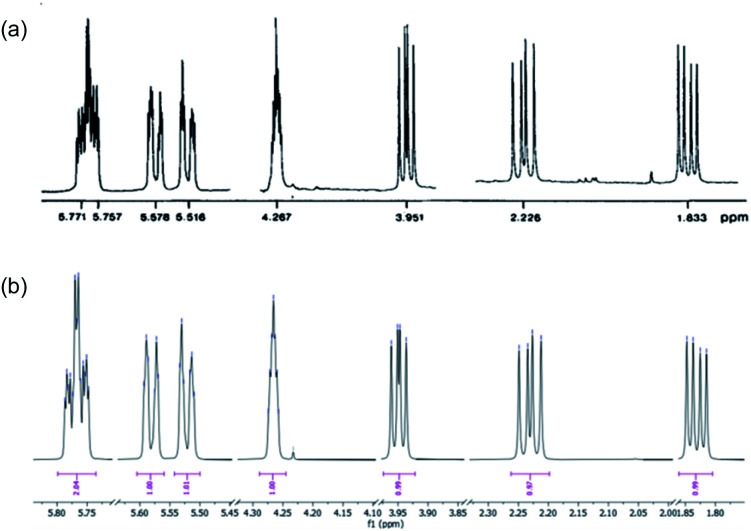
^1^H NMR spectrum of spiroarogenate **9** (a) isolated from *Neurospora crassa* (400 MHz in D_2_O);^19^ (b) synthesized by the method of Scheme 2 (400 MHz in D_2_O).

The purification of **9** and its separation from inorganic salts proved problematic. The ion-exchange chromatography reported^6,7^ for the purification of spiroarogenate and arogenate was ineffective, as a consequence of the dilution required by the barium salts. In acidic solution, **9** underwent rearomatizing fragmentation (with a half-life measured in hours at pH 5: see ESI†) to phenylalanine, preventing the use of standard reversed-phase chromatography. HPLC using hydrophilic interaction liquid chromatography (HILIC) columns was successful at basic pH, but separations were poor. Eventually, successful results were obtained using a porous graphitic carbon stationary phase in the form of a Hypercarb™ column, which successfully removed more retained aromatic impurities giving a 27% isolated yield of air-stable spiroarogenate **9**. The ^1^H NMR spectrum of **9** ([*α*]^23^_D_ = −40) matched that of spiroarogenate isolated from *Neurospora crassa* (Fig. 2).^20^

Danishefsky *et al.* reported the hydrolysis of **9** to **1** using NaOH and Na_2_CO_3_,^6^ and we were able to form traces of arogenate from **9** by this method. However, purification of **9** using a gradient of 0–80% acetonitrile in 100 mM ammonium bicarbonate buffer revealed that the conditions that formed spiroarogenate **9** from **8a** also formed a trace of arogenate **1**, along with another by-product, the imidazolidinone **11**. Raising the temperature of the hydrolysis of **8a** with Ba(OH)_2_ to 70 °C for 5 hours increased the yield of arogenate to 55% (by ^1^H NMR). The free acid of arogenate **1** was even more unstable than spiroarogenate **9**, decomposing instantly by rearomatization to phenylalanine. Use of a Na_2_CO_3_/NaHCO_3_ buffer (10 mM at pH 9.2) in place of the ammonium bicarbonate buffer solved this problem, with material produced by aqueous hydrolysis of **8a** at 80 °C with Ba(OH)_2_ (3 equiv.) and BaCO_3_ (1 equiv.)^22^ giving a sample of the stable disodium salt of arogenate, **1-Na** in 41% yield, calculated by quantitative NMR.

**Scheme 3:**
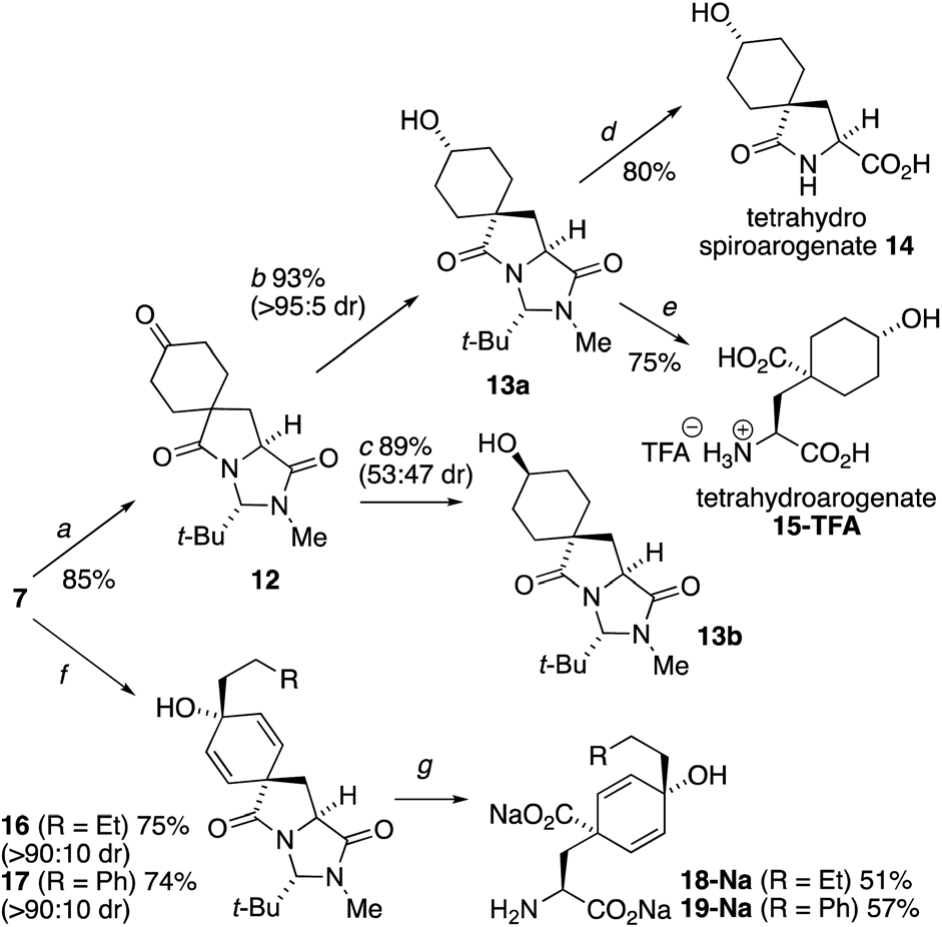
Arogenate analogues stabilized towards aromatization. Reagents and conditions: (a) H_2_, Pd/C, THF, rt, 16 h; (b) K-selectride (1.6 equiv.), THF, −78 °C, 1 h; (c) NaBH_4_ (3 equiv.), MeOH, 0 °C – rt, 18 h; (d) NaOH, H_2_O, 90 °C, 16 h; (e) Ba(OH)_2_, BaCO_3_, H_2_O, 80 °C, 20 h; (f) RCH_2_CH_2_MgBr, THF, −78 °C – rt, 2 h; (g) NaOH, H_2_O, EtOH, 70 °C, 20 h.

**Fig. 3:**
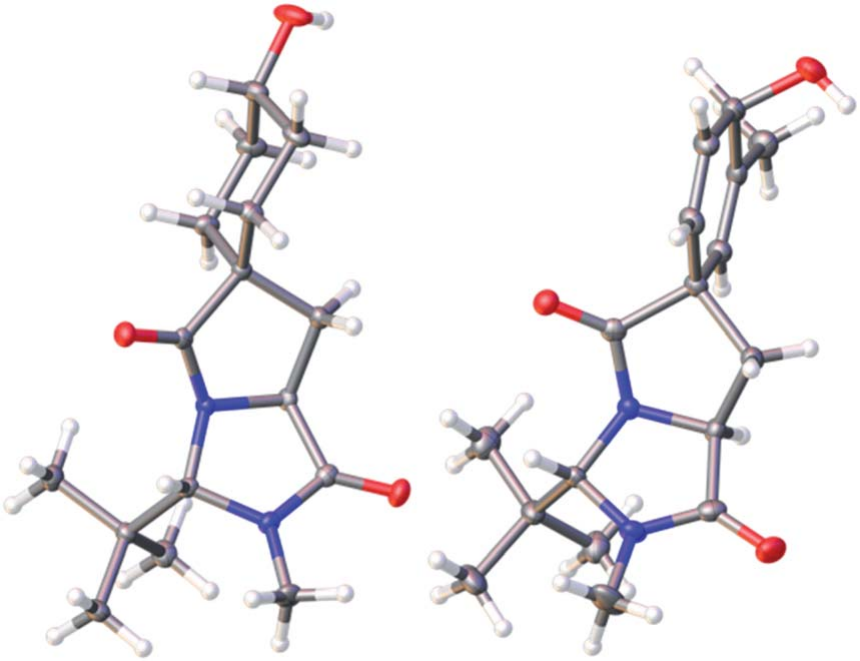
(a) X-ray crystal structure of **13b**; (b) X-ray crystal structure of **24d**.

The extreme instability of arogenate towards aromatization prompted us to explore modifications to the route that might provide more stable analogues with potential future utility as probes or modifiers of the function of arogenate-metabolising enzymes. Two alternative approaches, both starting from the spirodienone **7**, are shown in Scheme 3. Hydrogenation of **7** gave the cyclohexanone **12**, which was reduced with remarkably high diastereoselectivity (>95 : 5) to alcohol **13a** in 93% yield using K-selectride, or less selectively to a separable 47 : 53 mixture of **13a** and its crystalline alcohol epimer **13b** with NaBH_4_. The X-ray crystal structure of **13b** (Fig. 3a) confirmed the relative stereochemistry of this pair of diastereoisomers. Heating **13a** with 3 eq. NaOH in H_2_O at 90 °C gave tetrahydrospiroarogenate **14** in a yield of 80%. Modification of the hydrolysis conditions to 12 equiv. Ba(OH)_2_ and 1 eq. BaCO_3_ in H_2_O at 80 °C led directly to tetrahydroarogenate **15**, which was isolated after reversed-phase column chromatography as its TFA salt in 75% yield. Importantly, no elimination or other decomposition products were observed from these saturated analogues, giving them potential value in future biological studies.^23^

An alternative approach to stabilized arogenate analogues entailed the addition of an additional alkyl substituent at the 4-position of the dienol ring of **1**. A phenethyl moiety was selected with the possibility that analogues of this type may also mimic NADP in the binding pocket of arogenate dehydrogenase (see ESI†).^24^**16** and **17** were made (dr > 9 : 1) by 1,2-addition of Grignard reagents to the carbonyl group of the dienone **7**.^25^ The major diastereomers **16a** and **17a** were subjected to hydrolysis conditions. Heating **16a** and **17a** with 6 eq. NaOH in EtOH : H_2_O 2 : 1 at 70 °C gave the alkylated arogenate derivatives, **18** and **19**, in 51% and 57% yield.^26^**19** had increased stability in basic conditions but attempted isolation of the free acid forms of these products led to instantaneous rearomatization to their respective phenylalanine derivatives.

**Scheme 4:**
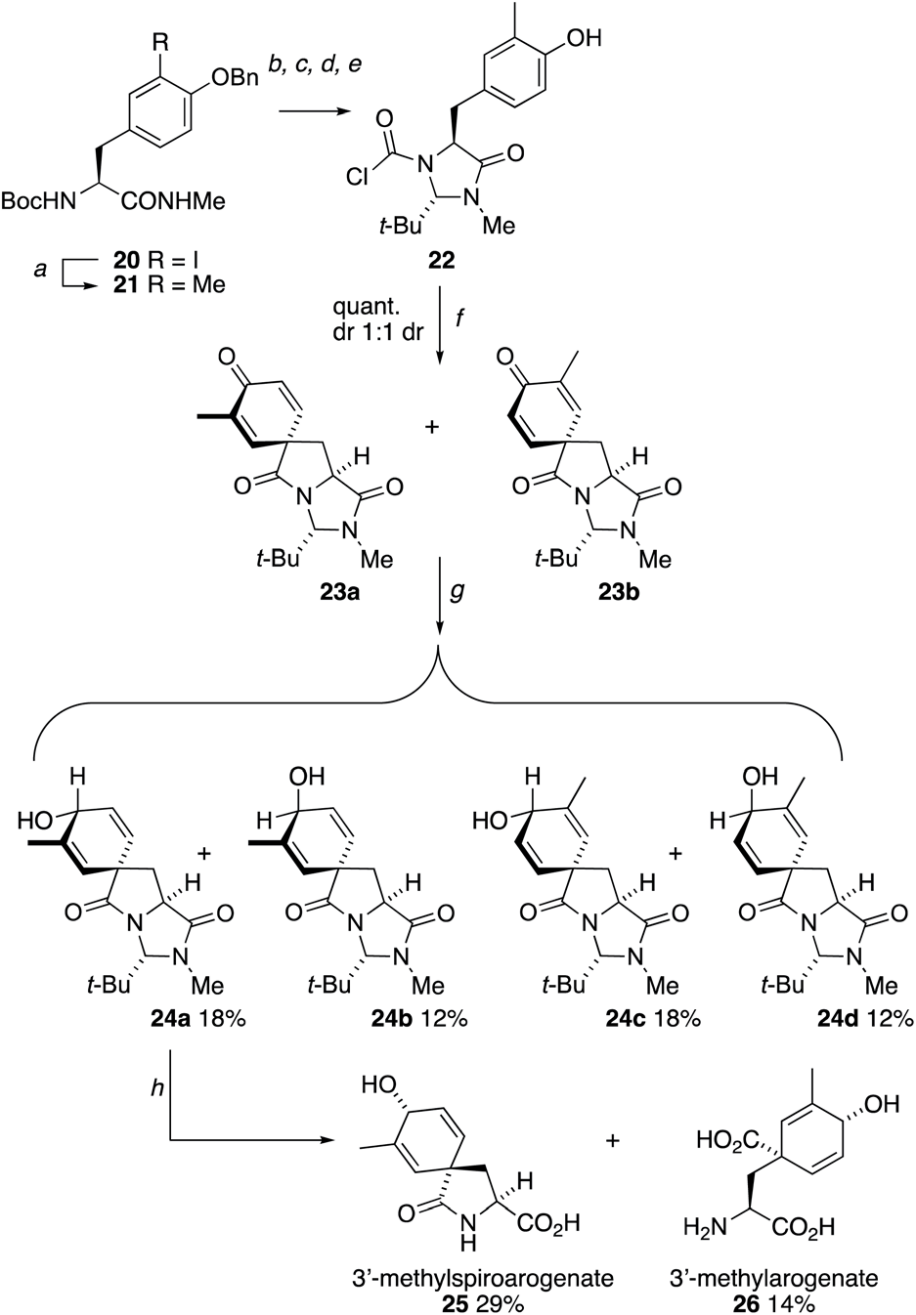
Synthesis of 3-methylarogenate **26** from 3-iodotyrosine. Reagents and conditions: (a) MeB(OH)_2_, Cs_2_CO_3_, SPhosPdG2, dioxane, 90 °C, 16 h; (b) MeNH_2_, 24 h; (c) *t*-BuCHO, MgSO_4_, 16 h; (d) COCl_2_, pyridine, THF, RT, 3 h; (e) H_2_, Pd/C, THF, RT, 4 h; (f) Et_3_N, MeCN, 150 °C (mw), 10 min; (g) NaBH_4_, CeCl_3_·7H_2_O, MeOH, −78 °C, 30 min; (h) Ba(OH)_2_, H_2_O, 40 °C, 16 h.

Other opportunities to synthesize ring-substituted arogenate derivatives entailed the spirocyclization of starting materials carrying substituted phenolic rings (Scheme 4).^27^ Starting from the protected form **20** of 3-iodo-l-tyrosine, a Suzuki coupling using methylboronic acid installed the 3-methyl substituent of **21**. Conversion of this compound into the chloroformylimidazolidinone **22** followed straightforwardly under the conditions used in Scheme 2. Spirocyclization of **22** was unaffected by the substitution on the ring, and heating with Et_3_N for 10 min at 150 °C in the microwave gave a 1 : 1 mixture of the two diastereoisomers of **23**. Reduction of these gave four diastereoisomers **24a–d** which were separated using standard column chromatography and were characterized through a combination of NOE and X-ray crystallography (the X-ray crystal structure of **24d** is shown in Fig. 3b). Hydrolysis using Ba(OH)_2_ at 40 °C yielded samples of the methylated spiroarogenate and arogenate analogues **25** and **26**. The free acid form of **26** showed significantly greater stability towards rearomatization than arogenate itself.

## Conclusions

In summary, we report a synthesis of the primary metabolite arogenate **1** in seven steps and an overall yield of 20%, an order of magnitude improvement on the maximum yield previously published.^7^ We also report the synthesis of the natural product spiroarogenate **9**. Both syntheses proceed through a synthetically versatile dienone intermediate **7**, amenable to regiodiverse functionalization. Arogenate and spiroarogenate display significant instability towards rearomatization under acidic or basic conditions, but by modifying the synthetic route to introduce saturation or alkyl substituents we were able to make analogues that are significantly more stable. Additionally, the ability to convert Tyr back to arogenate efficiently opens up the possibility of making isotopically labelled samples of arogenate for use in biosynthetic studies. Exploration of the potential utility of these analogues and isotopomers as tools for the investigation and modification of plant metabolism are under way.

## Author contributions

LE, DJL, JWW, and JC conceived the project. JC directed the work. JSW provided scientific and practical input and advice. LE, DJL, MS, IU, GB and JWW carried out the experiments. LE, DJL, JWW and JC wrote the paper.

## Conflicts of interest

There are no conflicts to declare.

## Supplementary Material

SC-012-D1SC03554A-s001

SC-012-D1SC03554A-s002
