# Supplementary material for: ‘Reverse biomimetic’ synthesis of l-arogenate and its stabilized analogues from l-tyrosine
Source: Chem Sci. 2021 Jul 30;12(34):11394–8. doi: 10.1039/d1sc03554a (PMC8447241; doi:10.1039/d1sc03554a)
Supplement: SC-012-D1SC03554A-s001 [file SC-012-D1SC03554A-s001.pdf]

Electronic Supplementary Material (ESI) for Chemical Science.
This journal is © The Royal Society of Chemistry 2021

Supplementary Material for

'Reverse Biomimetic' Synthesis of L-Arogenate and its Stabilized Analogues from L-Tyrosine

Louise Eagling,[†] Daniel J. Leonard,[†] Maria Schwarz,[†] John W. Ward,[†] Iñaki Urruzuno,[†] Grace Boden,[†] J. Steven Wailes,[⊥] and Jonathan Clayden[†*]

[†] School of Chemistry, University of Bristol, Cantock's Close, Bristol, BS8 1TS, United Kingdom

[⊥]Syngenta, Jealott's Hill International Research Centre, Bracknell, Berkshire, RG42 6EY, United Kingdom

Table of Contents

1. General Information 2
2. Experimental 3
   2.1 Synthesis of arogenate 3
   2.2. Arogenate derivatives 10
      2.2.1. 3-Methyl Arogenate 10
      2.2.2. Grignard addition 21
      2.2.3. Hydrogenation 25
3. Additional data 30
   3.1 Hydrolysis optimization 30
   3.2 Spirocyclisation optimization 32
4. NMR Spectra 33
5. CD data 66
6. References 66

1. General Information

Unless stated, all reagents and chemicals were bought from commercial suppliers and used without further purification.

When anhydrous conditions were necessary, reactions were carried out under nitrogen in flame-dried glassware using standard Schlenk syringe-septa techniques.

Anhydrous dichloromethane, THF, MeCN and toluene were dried by passing through a modified Grubbs system of alumina columns, manufactured by Anhydrous Engineering. Petrol Ether indicates fractions of petroleum ether boiling at 40-60 °C.

Thin layer chromatography (TLC) was performed with aluminium backed silica TLC plates (Merck-Keiselgel 60 F254) with a suitable solvent system. Visualisation was *via* UV light (at 254 nm) or by staining with potassium permanganate solution or 'Seebach' dip and heat.

Flash column chromatography was performed on a Biotage Isolera™ four system using 10, 25, 50 or 100 g silica or C18 Biotage® SNAP columns and a variable 200-400 nm wavelength detector scanning all wavelengths.

Preparative HPLC purification was performed with a Waters AutoPurification System with a Waters System Fluidics Organizer, a Waters 2545 Binary Gradient Module, a Waters 2767 Sample Manager and a Waters 2998 Photodiode Array Detector on a Hypercarb™ column.

Infrared spectra were recorded on a Perkin Elmer Spectrum (Spectrum One) FT-IR with an ATR accessory and frequencies are reported in wavenumbers (cm^{-1}).

^{1}H and ^{13}C NMR spectra were recorded in $CDCl_3$ solutions (unless otherwise stated) on Jeol-ECS 400, Varian 400/500, Bruker 400 and Bruker 500 (cryo-enhanced ^{13}C probe) spectrometers at ambient temperature, and were referenced to the residual deuterated solvent peak for $CDCl_3$ (7.26 ppm for 1H and 77.16 ppm for 13C) or D_2O (4.79 ppm for 1H). Chemical shifts (δ) are reported in parts per million (ppm) and coupling constants (*J*) in hertz (Hz). Multiplicities are denoted as singlet (s), doublet (d), triplet (t), quartet (q) or multiplet (m). COSY, HMBC and HSQC NMR spectra were routinely used to definitively assign the signals of ^{1}H and ^{13}C NMR spectra.

Electrospray (ESI) mass spectra were recorded on a Bruker Daltronics MicrOTOF 2 mass spectrometer.

Nanospray mass spectra were recorded on a UHPLC-MS (Dionex RS3000 LC system).

Melting points were measured on a Stuart Scientific melting point SMP 10 apparatus and are uncorrected.

Optical rotations ($[\alpha]_D^T$) were measured on a Bellingham and Stanley Ltd. ADP220 polarimeter where c is given in g/100 mL.

Suitable X-ray crystallography samples were grown by slow diffusion technique.

2. Experimental

2.1 Synthesis of arogenate

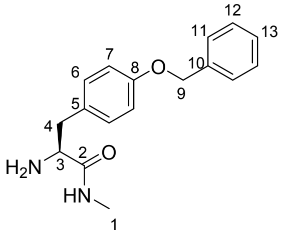

(*S*)-2-amino-3-(4-(benzyloxy)phenyl)-*N*-methylpropanamide (3)

To a solution of *O*-Benzyl-L-tyrosine methyl ester hydrochloride (5 g, 15.5 mmol) in EtOH (31 mL), methylamine (33 % wt. in EtOH)(27 mL, 217.6 mmol) was added. The mixture was stirred at room temperature for 2 days. The solution was concentrated *in vacuo*, redissolved in dichloromethane (80 mL) and washed with a saturated aqueous solution of $NaHCO_3$ (50 mL). The aqueous phase was extracted with dichloromethane (3 x 30 mL). The combined organics were washed with brine (30 mL), dried over $MgSO_4$ and concentrated in *vacuo* to yield **3** (4.06 g, 92 %) as a white powder.

^{1}H NMR (400 MHz, Chloroform-*d*) δ 7.46 – 7.27 (m, 5H, $H_{11},_{12},_{13}$), 7.12 (d, *J* = 8.6 Hz, 2H, H_6), 6.92 (d, *J* = 8.6 Hz, 2H, H_7), 5.04 (s, 2H, H_9), 3.55 (dd, *J* = 9.3, 4.0 Hz, 1H, H_3), 3.19 (dd, *J* = 13.9, 4.0 Hz, 1H, H_4), 2.80 (d, *J* = 5.0 Hz, 3H, H_1), 2.63 (dd, *J* = 13.9, 9.3 Hz, 1H, H_4)

^{13}C NMR (100 MHz, Chloroform-d) δ 174.86 (C_2), 157.74 (C_8), 137.01 (C_{10}), 130.28 (C_6), 130.21 (C_5), 128.59 (C_{12}), 127.97 (C_{13}), 127.45 (C_{11}), 115.07 (C_7), 70.06 (C_9), 56.57 (C_3), 40.19 (C_4), 25.81 (C_1)

HRMS-ESI (*m/z*) $[M+H]^+$ calc. for $C_{17}H_{21}N_2O_2$ 285.1597, found 285.1584

R$_f$ - 0.13 in 2% MeOH in EtOAc

mp 123-125 °C

[α] $_D^{20}$ = -60

Data in agreement with reported values[1]

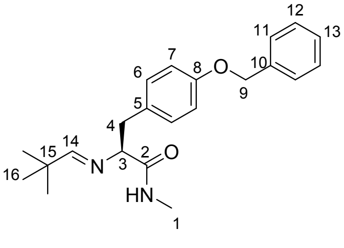

(*S,E*)-2-(benzylideneamino)-3-(4-(benzyloxy)phenyl)-*N*-methylpropanamide (4)

To a solution of **3** (4.06 g, 14.3 mmol) in dichloromethane (14 mL), $MgSO_4$ (4 g, 100% wt.) and pivaldehyde (1.87 mL, 17.2 mmol) was added. The mixture was stirred at room temperature overnight. The solution was filtered and washed with dichloromethane (10 mL). The filtrate was concentrated *in vacuo* to yield **4** (4.62 g, 99 %) as a white powder.

^{1}H NMR (400 MHz, Chloroform-*d*) δ 7.45 – 7.29 (m, 5H, $H_{11,12,13}$), 6.98 (d, *J* = 8.5 Hz, 2H, H_6), 6.88 – 6.83 (m, 3H, H_{14}, H_7), 5.04 (s, 2H, H_9), 3.65 (dd, *J* = 10.4, 3.1 Hz, 1H, H_3), 3.28 (dd, *J* = 13.5, 3.1 Hz, 1H, H_4), 2.86 (d, *J* = 5.0 Hz, 3H, H_1), 2.69 (dd, *J* = 13.5, 10.4 Hz, 1H, H_4), 0.89 (s, 9H, H_{16})

^{13}C NMR (100 MHz, Chloroform-d) δ 174.24 (C_{14}), 173.44 (C_2), 157.31 (C_8), 137.07 (C_{10}), 131.01 (C_6), 130.00 (C_5), 128.52 (C_{12}), 127.85 (C_{13}), 127.32 (C_{11}), 114.58 (C_7), 74.49 (C_3), 69.95 (C_9), 40.06 (C_4), 36.21 (C_{15}), 26.63 (C_{16}), 25.86 (C_1)

HRMS-ESI (*m/z*) $[M+H]^+$ calc. for $C_{22}H_{29}N_2O_2$ 353.2224, found 353.2225

IR vmax – 3370, 3349, 2892 (C-H), 1636 (C=O)

Data in agreement with reported values[1]

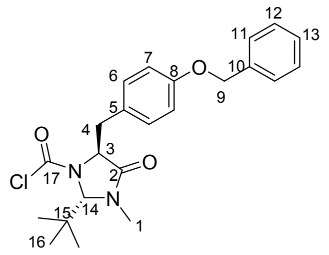

(2S,5S)-5-(4-(benzyloxy)benzyl)-2-(tert-butyl)-3-methyl-4-oxoimidazolidine-1-carbonyl chloride (5)

To a solution of **4** (3.1 g, 9.45 mmol) in THF (19 mL), phosgene (10.15 mL, 15%, 14.2 mmol) was added. The mixture was stirred at room temperature for 2 hours. Pyridine (1.5 mL, 18.9 mmol) was then added and the mixture stirred for a further 1 hour. The solution was concentrated *in vacuo* and redissolved in dichloromethane (30 mL). The organic phase was washed with HCl (1M, 50 mL) and extracted with dichloromethane (3 x 30 mL). The combined organics were washed with brine (20 mL), dried over $MgSO_4$ and concentrated in *vacuo*. Purification was carried out by trituration using diethyl ether to yield **5** (2.85 g, 72 %) as a pale-yellow powder.

NMR reported as a rotameric mixture

^{1}H NMR (500 MHz, Chloroform-*d*) δ 7.45 – 7.36 (m, 5H, $H_{11,12,13}$), 7.09 (d, *J* = 8.2 Hz, 2H, H_6), 6.86 (d, *J* = 8.2 Hz, 2H, H_7), 5.03 (s, 2H, H_9), 4.73 (s, 0.55H, H_{14}), 4.62 (s, 0.45H, H_{14}), 4.42 (s, 0.55H, H_3), 4.39 (s, 0.45H, H_3), 3.80 (d, *J* = 14.6 Hz, 0.45H, H_4), 3.69 (d, *J* = 14.6 Hz, 0.55H, H_4), 3.29 (d, *J* = 14.5 Hz, 0.45H, H_4), 3.21 (d, *J* = 14.5 Hz, 0.55H, H_4) 2.87 (s, 1.65H, H_1), 2.73 (s, 1.35H, H_1), 1.01 (s, 4.65H, H_{16}), 0.94 (s, 4.35H, H_{16})

^{13}C NMR (125 MHz, Chloroform-*d*) δ 169.82 (C_2), 169.32 (C_2), 158.07 (C_8), 157.88 (C_8), 147.30 (C_{17}), 146.06 (C_{17}), 136.97 (C_{10}), 136.89 (C_{10}), 131.21 (C_6), 131.05 (C_6), 128.55 (C_{12}), 127.96 (C_{13}), 127.56 (C_{11}), 126.50 (C_5), 126.10

(C_5), 114.75 (C_7), 114.58 (C_7), 83.40 (C_{14}), 82.79 (C_{14}), 69.92 (C_9), 63.71 (C_3), 62.79 (C_3), 41.71 (C_{15}), 41.57 (C_{15}), 35.77 (C_4), 32.05 (C_1), 31.93 (C_4), 26.70 (C_{16}), 26.44 (C_{16})

HRMS-ESI (*m/z*) $[M+H]^+$ calc. for $C_{23}H_{28}ClN_2O_3$ 415.1783, found 415.1782

IR vmax – 2965 (C-H), 1710 (C=O)

R_f - 0.36 in 100% dichloromethane

Data in agreement with reported values[1]

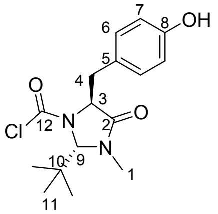

(2S,5S)-2-(tert-butyl)-5-(4-hydroxybenzyl)-3-methyl-4-oxoimidazolidine-1-carbonyl chloride (6)

To a solution of **5** (1.25 g, 3.02 mmol) in THF (30 mL) under N_2, palladium on active carbon (10 wt. %) (250 mg, 20 % wt.) was added. The mixture was then stirred at room temperature for 4 hours under hydrogen atmosphere. The reaction mixture was filtered over a pad of celite and concentrated in *vacuo* to yield **6** (978 mg, 95 %) as a white powder.

^{1}H NMR (400 MHz, Chloroform-d) δ 7.04 (d, J = 8.0 Hz, 2H, H_6), 6.72 (m, 2H, H_7), 4.75 (s, 0.53H, H_9), 4.64 (s, 0.47H, H_9), 4.40 (d, J = 11.9 Hz, 1H, H_3), 3.81 (d, J = 14.6 Hz, 0.47H, H_4), 3.68 (d, J = 14.6 Hz, 0.53H, H_4), 3.29 (d, J = 14.6 Hz, 0.47H, H_4), 3.20 (d, J = 14.6 Hz, 0.53H, H_4), 2.89 (s, 1.61H, H_1), 2.76 (s, 1.38H, H_1), 1.01 (s, 4.82H, H_{11}), 0.94 (s, 4.18H, H_{11}).

^{13}C NMR (126 MHz, Chloroform-d) δ 170.13 (C_2), 169.61 (C_2), 155.33 (C_8), 154.99 (C_8), 147.56 (C_{12}), 146.23 (C_{12}), 131.50 (C_6), 131.33 (C_6), 126.23 (C_5), 125.79 (C_5), 115.47 (C_7), 115.28 (C_7), 83.62 (C_9), 83.02 (C_9), 63.88 (C_3), 62.99 (C_3), 41.84 (C_{10}), 41.70 (C_{10}), 35.84 (C_4), 32.23 (C_1), 32.00 (C_4), 26.83 (C_{11}), 26.56 (C_{11}).

HRMS-ESI (*m/z*) $[M+H]^+$ calc. for $C_{16}H_{22}ClN_2O_3$ 325.1313, found 325.1311

IR vmax – 3375 (O-H), 2924 (C-H), 1742, 1694 (C=O)

R_f - 0.63 in 100% EtOAc

mp 150-151 °C

$[\alpha]_D^{20}$ = -28

Table 1 Crystal data and structure refinement for 6.

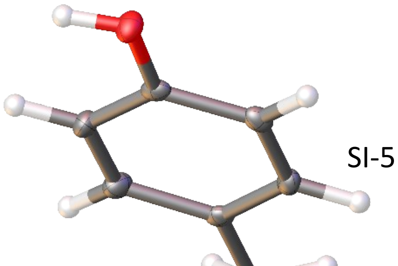

CCDC deposition number	2070018
Empirical formula	$C_{16}H_{21}ClN_2O_3$
Formula weight	324.80
Temperature/K	100(2)
Crystal system	orthorhombic
Space group	$P2_12_12_1$
a/Å	10.8233(2)
b/Å	11.9161(3)
c/Å	12.3076(3)
α/°	90
β/°	90
γ/°	90
Volume/Å^3	1587.33(6)
Z	4
ρ_{calc}g/cm^3	1.359
μ/mm^{-1}	0.255
F(000)	688.0
Crystal size/mm^3	0.426 × 0.396 × 0.166
Radiation	MoKα (λ = 0.71073)
2Θ range for data collection/°	4.758 to 55.812
Index ranges	-8 ≤ h ≤ 14, -15 ≤ k ≤ 15, -16 ≤ l ≤ 16
Reflections collected	14647
Independent reflections	3800 [R_{int} = 0.0395, R_{sigma} = 0.0362]
Data/restraints/parameters	3800/0/207
Goodness-of-fit on F^2	1.023
Final R indexes [I>=2σ (I)]	R_1 = 0.0298, wR_2 = 0.0669
Final R indexes [all data]	R_1 = 0.0347, wR_2 = 0.0691
Largest diff. peak/hole / e Å^{-3}	0.22/-0.21
Flack parameter	0.01(3)

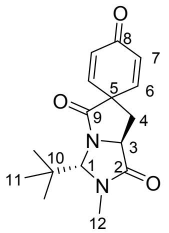

(7a'*S*)-3'-(*tert*-butyl)-2'-methyl-2',3',7',7a'-tetrahydro-1'H,5'H-spiro[cyclohexane-1,6'-pyrrolo[1,2-c]imidazole]-2,5-diene-1',4,5'-trione (7)

To a solution of **6** (5.2 g, 15.2 mmol) in MeCN (24 mL), triethylamine (2.7 mL, 19.1 mmol) was added. The mixture was then heated under microwave irradiation at 150 °C for 10 minutes. The reaction mixture was concentrated *in vacuo*, redissolved in dichloromethane (40 mL) and washed with water (20 mL). The aqueous phase was extracted with dichloromethane (3 x 30 mL). The combined organics were washed with brine (30 mL), dried over $MgSO_4$, filtered and concentrated *in vacuo* to yield **7** (4.37 g, Quant.) as a yellow powder.

^{1}H NMR (400 MHz, Chloroform-*d*) δ 6.92 (dd, *J* = 10.1, 3.0 Hz, 1H, H_6), 6.71 (dd, *J* = 10.1, 3.0 Hz, 1H, H_6), 6.47 (dd, *J* = 10.1, 1.8 Hz, 1H, H_7), 6.40 (dd, *J* = 10.1, 1.8 Hz, 1H, H_7), 4.85 (d, *J* = 1.1 Hz, 1H, H_1), 4.45 (t, *J* = 8.2 Hz, 1H, H_3), 3.03 (s, 3H, H_{12}), 2.61 (dd, *J* = 13.3, 7.4 Hz, 1H, H_4), 2.46 (dd, *J* = 13.3, 9.0 Hz, 1H, H_4), 1.04 (s, 9H, H_{11})

^{13}C NMR (100 MHz, Chloroform-d) δ 184.63 (C_8), 173.86 (C_2), 171.32 (C_9), 146.11 (C_6), 143.51 (C_6), 132.24 (C_7), 130.86 (C_7), 81.87 (C_1), 56.85 (C_3), 53.89 (C_5), 38.35 (C_{10}), 37.29 (C_4), 31.44 (C_{12}), 25.77 (C_{11})

HRMS-ESI (*m/z*) $[M+Na]^+$ calc. for $C_{16}H_{21}N_2NaO_3$ 311.1366, found 311.1365

IR vmax – 2966 (C–H), 1698 (C=O)

R_f - 0.31 in 100% EtOAc

mp 193-195 °C

[α] $^{26}_{D}$ = 8 ° (*c* = 1 in $CHCl_3$)

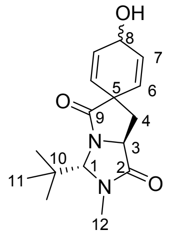

(7a'*S*)-3'-(*tert*-butyl)-4-hydroxy-2'-methyl-2',3',7',7a'-tetrahydro-1'H,5'H-spiro[cyclohexane-1,6'-pyrrolo[1,2-c]imidazole]-2,5-diene-1',5'-dione (8)

To a solution of **7** (480 mg, 1.67 mmol) in dry methanol (17 mL) under N_2, cerium(III) chloride heptahydrate (95 mg, 2.5 mmol) was added and stirred for 15 minutes at room temperature. The reaction mixture was then cooled to -78 °C and sodium borohydride (930 mg, 2.5 mmol) was added. The resultant mixture was left to stir for a further 30 minutes and then quenched with water (10 mL). The mixture was then diluted with EtOAc (50 mL) and washed with water (30 mL). The aqueous phase was extracted with EtOAc (3 x 30 mL) and brine (30 mL). The organic phase was dried over $MgSO_4$, filtered and concentrated *in vacuo*. Purification was carried out by column chromatography on silica gel eluting with 0-10% MeOH in dichloromethane to yield **8** (463 mg, 95 %, d.r. = 3.1:1) as white powders.

Major - 8a

^{1}H NMR (400 MHz, Chloroform-d) δ 6.23 (ddd, J = 9.9, 3.9, 1.7 Hz, 1H, H_6), 6.14 (ddd, J = 9.9, 3.9, 1.7 Hz, 1H, H_6), 5.95 (ddd, J = 9.9, 2.3, 1.0 Hz, 1H, H_7), 5.66 (ddd, J = 9.9, 2.3, 1.1 Hz, 1H, H_7), 4.80 (d, J = 1.1 Hz, 1H, H_1), 4.48 (dtt, J = 11.0, 3.9, 1.1 Hz, 1H, H_8), 4.41 – 4.32 (m, 1H, H_3), 3.00 (s, 3H, H_{12}), 2.49 (dd, J = 13.2, 7.7 Hz, 1H, H_4), 2.16 (dd, J = 13.2, 8.7 Hz, 1H, H_4), 1.03 (s, 9H, H_{11})

^{13}C NMR (100 MHz, Chloroform-*d*) δ 177.84 (C_2), 172.27 (C_9), 131.56 (C_6), 130.72 (C_6), 129.74 (C_7), 128.50 (C_7), 81.82 (C_1), 61.45 (C_8), 56.78 (C_3), 50.62 (C_5), 38.90 (C_4), 38.38 (C_{10}), 31.37 (C_{12}), 25.85 (C_{11})

HRMS-ESI (*m/z*) $[M+Na]^+$ calc. for $C_{16}H_{22}N_2NaO_3$ 313.1522, found 313.1521

IR vmax – 3392 (O-H), 3029, 2964, 2871 (C-H), 1695 (C=O)

R_f - 0.23 in 5% MeOH in dichloromethane

mp 58-59 °C

$[\alpha]_D^{26}$ = -4 ° (*c* = 1 in $CHCl_3$)

<u>Minor – 8b</u>

^{1}H NMR (400 MHz, Chloroform-*d*) δ 6.19 (ddd, *J* = 9.9, 3.1, 2.0 Hz, 1H, H_6), 6.07 (ddd, *J* = 9.9, 3.1, 2.0 Hz, 1H, H_6), 5.92 (dt, *J* = 9.9, 2.0 Hz, 1H, H_7), 5.63 (dt, *J* = 9.9, 2.0 Hz, 1H, H_7), 4.82 (d, *J* = 1.2 Hz, 1H, H_1), 4.71 (dtt, *J* = 8.9, 3.2, 1.7 Hz, 1H, H_8), 4.38 (t, *J* = 8.2 Hz, 1H, H_3), 3.03 (d, *J* = 0.6 Hz, 3H, H_{12}), 2.51 (dd, *J* = 13.2, 7.5 Hz, 1H, H_4), 2.23 (dd, *J* = 13.2, 8.9 Hz, 1H, H_4), 1.04 (s, 9H, H_{11})

HRMS-ESI (*m/z*) $[M+Na]^+$ calc. for $C_{16}H_{22}N_2NaO_3$ 313.1522, found 313.1522

IR vmax – 3335 (O-H), 2922, 2851 (C-H), 1692 (C=O)

R_f - 0.15 in 5% MeOH in dichloromethane

mp >220 °C decomposition

$[\alpha]_D^{26}$ = 8 ° (*c* = 1 in $CHCl_3$)

Table 2 Crystal data and structure refinement for 8b.

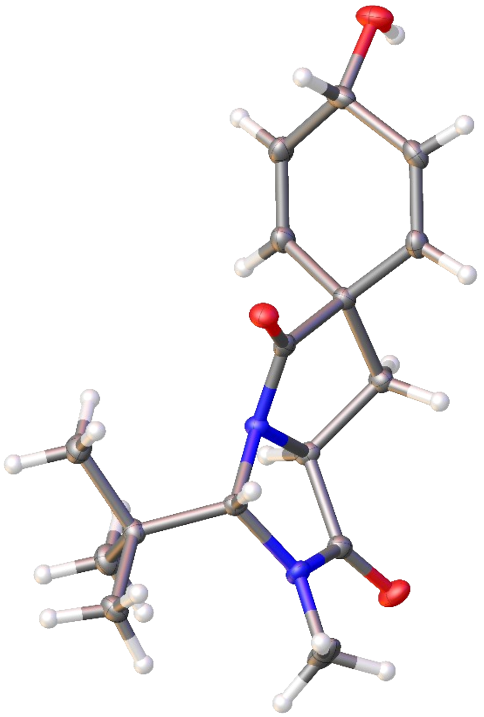

CCDC deposition number	2070019
Empirical formula	$C_{16}H_{22}N_2O_3$
Formula weight	290.35
Temperature/K	100(2)

Crystal system	monoclinic
Space group	$P2_1$
a/Å	5.7583(3)
b/Å	13.5711(6)
c/Å	9.6541(5)
α/°	90
β/°	95.964(3)
γ/°	90
Volume/Å^3	750.35(6)
Z	2
ρ_{calc}g/cm^3	1.285
μ/mm^{-1}	0.089
F(000)	312.0
Crystal size/mm^3	0.534 × 0.23 × 0.136
Radiation	MoKα (λ = 0.71073)
2Θ range for data collection/°	4.242 to 55.984
Index ranges	$-7 \le h \le 7$, $-17 \le k \le 15$, $-12 \le l \le 12$
Reflections collected	10338
Independent reflections	3447 [R_{int} = 0.0447, R_{sigma} = 0.0519]
Data/restraints/parameters	3447/1/198
Goodness-of-fit on F^2	1.025
Final R indexes [I>=2σ (I)]	R_1 = 0.0411, wR_2 = 0.0824
Final R indexes [all data]	R_1 = 0.0519, wR_2 = 0.0867
Largest diff. peak/hole / e Å^{-3}	0.23/-0.21
Flack parameter	-0.6(7)

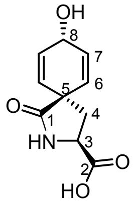

(3*S*,5r,8*S*)-8-hydroxy-1-oxo-2-azaspiro[4.5]deca-6,9-diene-3-carboxylic acid/Spiroarogenate (9)

To a solution of **8a** (120 mg, 0.41 mmol) in water (2 mL), barium hydroxide was added (213 mg, 1.24 mmol). The mixture was stirred at 30 °C overnight, then sodium carbonate anhydrous (263 mg, 2.48 mmol) and water (5 mL) were added. The resulting barium carbonate salt was separated by filtration and the aqueous layer was washed with dichloromethane (15 mL). The aqueous phase was then neutralised (approx. pH = 8) and lyophilized. The mixture was redissolved in ethanol and filtered to remove excess salt. The product was purified by Hypercarb HPLC with a gradient of 0-100% acetonitrile in 100 mM ammonium bicarbonate buffer to yield **9** (21 mg, 27 %) as a white powder.

^{1}H NMR (600 MHz, Deuterium Oxide) δ 6.12 – 6.06 (m, 2H, H_7), 5.90 (dt, J = 10.1, 2.0 Hz, 1H, H_6), 5.84 (dt, J = 10.1, 2.0 Hz, 1H, H_6), 4.59 (tt, J = 3.4, 1.5 Hz, 1H, H_8), 4.27 (dd, J = 8.7, 6.3 Hz, 1H, H_3), 2.55 (dd, J = 13.3, 8.7 Hz, 1H, H_4), 2.15 (dd, J = 13.3, 6.3 Hz, 1H, H_4).

^{13}C NMR (600 MHz, Deuterium Oxide) δ 180.49 (C_1), 180.16 (Br, C_2), 129.97 (C_6), 129.92 (C_7), 129.85 (C_7), 129.37 (C_6), 61.32 (C_8), 55.83 (Br, C_3) 48.54 (C_5), 40.26 (C_4)

HRMS-Nanospray (*m/z*) $[M-H]^-$ calc. for $C_{11}H_{14}NO_5$, 208.0610; found 208.0612

CD spectra – Positive Cotton effect below 230 nm

[α] $_D^{23}$ = -40 ° (*c* = 0.1 in H_2O)

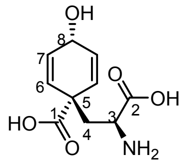

(1s,4*R*)-1-((*S*)-2-amino-2-carboxyethyl)-4-hydroxycyclohexa-2,5-diene-1-carboxylic acid/Arogenate (1)

To a solution of **8a** (32.2 mg, 0.11 mmol) in water (0.52 mL), barium hydroxide (52 mg, 0.32 mmol) and barium carbonate (20 mg, 0.11 mmol) were added. The mixture was stirred at 80 °C for 5 hours, then sodium carbonate anhydrous (70 mg, 0.63 mmol) and water (5 mL) were added. The resulting barium carbonate salt was filtered through a Whatman 0.45 µm Nylon syringe filter. The aqueous phase was lyophilized. The solid was redissolved in water (3 mL), filtered through a Millipore Millex-HN 0.45 µm Nylon syringe filter and purified by Hypercarb HPLC with a gradient of 0-80% acetonitrile in 10 mM sodium carbonate-bicarbonate buffer (pH 9.2) to yield **1** as the disodium salt (41 % by quantitative NMR using a stock solution of EtOH) as a white powder.

^{1}H NMR (500 MHz, Deuterium Oxide) δ 5.93 – 5.86 (m, 3H, $H_{6\&7}$), 5.82 – 5.76 (m, 1H, $H_{6\&7}$), 4.47 (d, *J* = 3.6 Hz, 1H, H_8), 3.66 (dd, *J* = 10.5, 3.0 Hz, 1H, H_3), 1.98 (dd, *J* = 14.3, 3.0 Hz, 1H, H_4), 1.89 (dd, *J* = 14.3, 10.5 Hz, 1H, H_4).

^{13}C NMR (126 MHz, Deuterium Oxide) δ 188.13 (C_1), 186.94 (C_2), 168.88 (Na_2CO_3), 138.06 (C_7), 137.81 (C_6), 133.68 (C_6), 132.19 (C_7), 67.36 (C_8), 60.57 (C_3), 56.12 (C_5), 46.94 (C_4).

CD spectra – Positive Cotton effect below 230 nm

HRMS-Nanospray (*m/z*) $[M-H]^-$ calc. for $C_{10}H_{12}NO_5$, 226.0715; found 226.0717

[α] $_D^{23}$ = -8 ° (*c* = 0.5 in H_2O)

2.2. Arogenate derivatives

2.2.1. 3-Methyl Arogenate

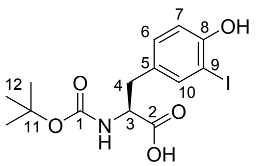

(*S*)-2-((*tert*-butoxycarbonyl)amino)-3-(4-hydroxy-3-iodophenyl)propanoic acid (21)

To a solution of 3-iodo-L-tyrosine (5 g, 16.3 mmol) in dioxane:water (80 mL, 1:1) sodium hydrogen carbonate (4.1 g, 48.9 mmol) was added portionwise. To this mixture boc anhydride (4.3 g, 19.6 mmol) was added and stirred at room temperature overnight. The resulting mixture was concentrated *in vacuo* to approx. 40 mL and the residue was acidified with 2M HCl to pH 2. The aqueous was then extracted with ethyl acetate (3 x 50 mL) and the organics was washed with brine (30 mL), dried over $MgSO_4$, and concentrated *in vacuo* to yield **21** (6.63 g, quant.) as a white powder.

^{1}H NMR (400 MHz, Chloroform-d) δ 7.48 (s, 1H, H_{10}), 7.06 (dd, J = 8.3, 2.1 Hz, 1H, H_6), 6.91 (d, J = 8.3 Hz, 1H, H_7), 4.95 (d, J = 7.9 Hz, 1H, N-H), 4.53 (d, J = 7.3 Hz, 1H, H_3), 3.19 – 2.91 (m, 2H, H_4), 1.44 (s, 9H, H_{12})

^{13}C NMR (125 MHz, Chloroform-d) δ 175.41 (C_2), 155.57 (C_1), 154.21 (C_8), 139.08 (C_{10}), 131.27 (C_6), 130.10 (C_5), 115.26 (C_7), 85.82 (C_9), 80.78 (C_{11}), 54.49 (C_3), 36.50 (C_4), 28.45 (C_{12})

IR νmax – 3326 (O-H), 2928 (C-H), 1715, 1684 (C=O)

Data in agreement with reported values[2]

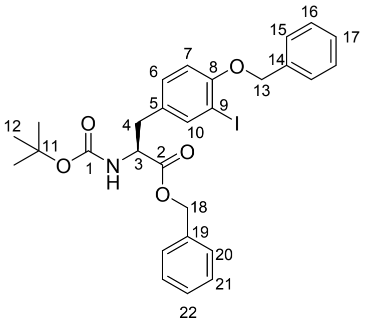

benzyl (*S*)-3-(4-(benzyloxy)-3-iodophenyl)-2-((*tert*-butoxycarbonyl)amino)propanoate (S1)

To a solution of **21** (6.3 g, 16.3 mmol) in DMF (65 mL), potassium carbonate (5.6 g, 40.75 mmol), benzyl bromide (4.85 mL, 40.75 mmol), and tetraethylammonium iodide (523 mg, 2.04 mmol) was added. The reaction mixture was stirred at room temperature overnight. The resultant mixture was diluted with ether (150 mL) and washed with water (50 mL). The aqueous phase was extracted with ether (3 x 50 mL) and the combined organics were washed with 1M HCl (50 mL) and brine (30 mL), dried over $MgSO_4$, and concentrated *in vacuo*. Purification was carried out by column chromatography on silica gel eluting with a gradient of 0-50% ethyl acetate in hexane to yield **S1** (8.89 g, 92 %) as a white powder.

^{1}H NMR (500 MHz, Chloroform-d) δ 7.55 (s, 1H, H_{10}), 7.49 (d, J = 7.1 Hz, 2H, H_{15}), 7.42 – 7.29 (m, 8H, $H_{16,17,20,21,22}$), 6.93 (d, J = 8.3 Hz, 1H, H_6), 6.68 (d, J = 8.3 Hz, 1H, H_7), 5.20 – 5.07 (m, 4H, $H_{13,18}$), 5.00 (d, J = 8.0 Hz, 1H, NH), 4.57 (q, J = 6.5 Hz, 1H, , H_3), 2.99 (qd, J = 13.9, 6.1 Hz, 2H, H_4), 1.43 (s, 9H, H_{12})

^{13}C NMR (125 MHz, Chloroform-d) δ 171.64 (C_2), 156.47 (C_8), 155.12 (C_1), 140.42 (C_{10}), 136.63 (C_{14}), 135.19 (C_{19}), 130.57 (C_6), 130.38 (C_5), 128.78 (C_{16}), 128.70 ($C_{20,21}$), 128.66 (C_{17}), 128.03 (C_{22}), 127.09 (C_{15}), 112.63 (C_7), 86.89 (C_9), 80.19 (C_{11}), 71.03 (C_{18}), 67.37 (C_{13}), 54.63 (C_3), 37.06 (C_4), 28.46 (C_{12})

HRMS-ESI (*m/z*) [M+Na$^+$] calc. for $C_{28}H_{30}INO_5Na$, 610.1061; found 610.1053

IR vmax – 3363 (N-H), 2975, 2931 (C–H), 1740, 1711 (C=O)

R$_f$ - 0.54 in 25% EtOAc in Hexane

Data in agreement with reported values[3]

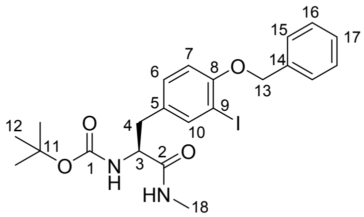

***tert*-butyl (*S*)-(3-(4-(benzyloxy)-3-iodophenyl)-1-(methylamino)-1-oxopropan-2-yl)carbamate (S2)**

To a solution of **S1** (8.7 g, 14.8 mmol) in EtOH (30 mL), methylamine (33 % wt. in EtOH)(12.9 mL, 103.6 mmol) was added. The mixture was stirred at room temperature for 2 days. The solution was concentrated *in vacuo*, redissolved in dichloromethane (80 mL) and washed with a saturated aqueous solution of $NaHCO_3$ (50 mL). The aqueous phase was extracted with dichloromethane (3 x 30 mL). The combined organics were washed with brine (30 mL), dried over $MgSO_4$ and concentrated in *vacuo* to yield **S2** (7.75 g, Quant.) as a white powder.

^{1}H NMR (500 MHz, Chloroform-d) δ 7.63 (d, J = 2.2 Hz, 1H, H_{10}), 7.51 – 7.44 (m, 2H, H_{15}), 7.39 (t, J = 7.4 Hz, 2H, H_{16}), 7.32 (t, J = 7.4 Hz, 1H, H_{17}), 7.10 (dd, J = 8.4, 2.2 Hz, 1H, H_6), 6.77 (d, J = 8.4 Hz, 1H, H_7), 5.78 (s, 1H, N-H), 5.12 (s, 2H, H_{13}), 5.07 – 4.96 (m, 1H, N-H), 4.21 (q, J = 7.2 Hz, 1H, H_3), 2.95 (d, J = 7.2 Hz, 2H, H_4), 2.74 (d, J = 4.8 Hz, 3H, H_{18}), 1.41 (s, 9H, H_{12})

^{13}C NMR (125 MHz, Chloroform-d) δ 171.63 (C_1), 156.38 (C_2), 155.50 (C_8), 140.29 (C_{10}), 136.57 (C_{14}), 131.45 (C_5), 130.41 (C_6), 128.70 (C_{16}), 128.06 (C_{17}), 127.13 (C_{15}), 112.76 (C_7), 86.99 (C_9), 71.05 (C_{13}), 56.15 (C_3), 37.51 (C_4), 28.44 (C_{12}), 26.32 (C_{18})

HRMS-ESI (*m/z*) [M+Na]$^+$ calc. for $C_{22}H_{27}IN_2O_4Na$, 533.0908; found 533.0888

IR vmax – 3325 (N-H), 2970, 2921, 2851 (C-H), 1683, 1649 (C=O)

R$_f$ - 0.51 in 10% MeOH in dichloromethane

mp 114-116 °C

[α] $_D^{26}$ = 12 ° (*c* = 1 in $CHCl_3$)

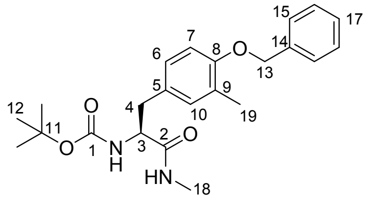

tert-butyl(_S_)-(3-(4-(benzyloxy)-3-methylphenyl)-1-(methylamino)-1-oxopropan-2-yl)carbamate (S3)

To a solution of **S2** (7.75 g, 15.2 mmol) in Dry degassed dioxane (100 mL), cesium carbonate (24.9 g, 76.4 mmol), SPhos Pd G_2 (1.01 g, 1.52 mmol) and Methyl boronic acid (4.6 g, 76.4 mmol). The mixture was stirred at 90 ^oC overnight. The solution was diluted with dichloromethane (100 mL) and water (100 mL). The aqueous phase was extracted with dichloromethane (3 x 50 mL). The combined organic phases dried over $MgSO_4$ and concentrated in *vacuo*. Purification was carried out by column chromatography on silica gel eluting with 0-100% EtOAc in petroleum ether to yield **S3** (4.43 g, 73 %) as a yellow powder.

^{1}H NMR (500 MHz, Chloroform-d) δ 7.45 – 7.41 (m, 2H, H_{15}), 7.38 (t, J = 7.5 Hz, 2H, H_{16}), 7.35 – 7.28 (m, 1H, H_{17}), 7.11 (d, J = 8.3 Hz, 0.25H, H_6), 6.99 (s, 1H, H_{10}), 6.95 (d, J = 8.3 Hz, 0.75H, H_6), 6.91 (m, 0.25H, H_7), 6.80 (d, J = 8.3 Hz, 0.75H, H_7), 5.66 (s, 1H, N-H), 5.05 (s, 2H, H_{13}), 4.21 (q, J = 7.5 Hz, 1H, H_3), 3.03 – 2.97 (m, 1H, H_4), 2.91 (dd, J = 13.7, 7.5 Hz, 1H, H_4), 2.72 (d, J = 4.9 Hz, 3H, H_{18}), 2.25 (s, 3H, H_{19}), 1.41 (s, 9H, H_{12})

^{13}C NMR (125 MHz, Chloroform-d) δ 172.02 (C_2), 156.01 (C_8), 155.54 (C_1), 137.51 (C_{14}), 131.79 (C_{10}), 130.46 (C_6), 128.72 (C_5), 128.65 (C_{16}), 127.92 (C_{17}), 127.61 (C_9), 127.58 (C_6), 127.23 (C_{15}), 115.16 (C_7), 111.66 (C_7), 80.24 (C_{11}), 70.03 (C_{13}), 56.37 (C_3), 38.11 (C_4), 28.44 (C_{12}), 26.26 (C_{18}), 16.49 (C_{19})

HRMS-ESI (*m/z*) $[M+Na]^+$ calc. for $C_{23}H_{30}N_2O_4Na$, 421.2098; found 421.2101

IR vmax – 3317 (N-H), 3063, 3032 (Ar-H), 2973, 2929, 2856 (C-H), 1704, 1658 (C=O)

R_f - 0.65 in dichloromethane

mp 134-135 °C

[α] $_D^{26}$ = 16 ° (*c* = 1 in $CHCl_3$)

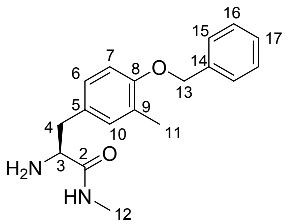

(S)-2-amino-3-(4-(benzyloxy)-3-methylphenyl)-N-methylpropanamide (S4)

To a solution of **S3** (4,3 g, 10.8 mmol) in MeOH (43 mL) at 0 ^oC, acetyl chloride (4.15 mL, 58.3 mmol) was slowly added. The mixture was warmed to room temperature and stirred overnight. The solution was diluted with dichloromethane (200 mL) and washed with saturated $NaHCO_3$ (50 mL) and brine (30 mL). The organic phase was dried over $MgSO_4$ and concentrated. Purification was carried out by column chromatography on silica gel eluting with a gradient of 0-10% methanol in dichloromethane to yield **S4** (3.11 g, 97%) as a yellow powder.

^{1}H NMR (500 MHz, Chloroform-d) δ 7.46 – 7.41 (m, 2H, H_{15}), 7.41 – 7.36 (m, 2H, H_{16}), 7.34 – 7.29 (m, 1H, H_{17}), 7.02 (d, J = 2.2 Hz, 1H, H_{10}), 6.98 (dd, J = 8.2, 2.2 Hz, 1H, H_6), 6.82 (d, J = 8.2 Hz, 1H, H_7), 5.06 (s, 2H, H_{13}), 3.57 (dd, J = 9.4, 4.1 Hz, 1H, H_3), 3.19 (dd, J = 13.8, 4.1 Hz, 1H, H_3), 2.82 (d, J = 5.0 Hz, 3H, H_{12}), 2.60 (dd, J = 13.8, 9.4 Hz, 1H, H_4), 2.27 (s, 3H, H_{11})

^{13}C NMR (125 MHz, Chloroform-d) δ 175.01 (C_2), 155.97 (C_8), 137.56 (C_{10}), 131.79 (C_{14}), 129.86 (C_5), 128.65 (C_{16}), 127.90 (C_{17}), 127.55 (C_6), 127.53 (C_9), 127.21 (C_{15}), 111.69 (C_7), 70.05 (C_{13}), 56.72 (C_3), 40.26 (C_4), 25.95 (C_{12}), 16.55 (C_{11})

HRMS-ESI (*m/z*) $[M+H]^+$ calc. for $C_{18}H_{23}N_2O_2$, 299.1754; found 299.1747

IR vmax – 3366, 3299 (N-H), 3064, 3032 (Ar-H), 2921 (C-H), 1654 (C=O)

R_f - 0.25 in 10% MeOH in dichloromethane

mp 78-80 °C

[α] $^{26}_{D}$ = -64 ° (*c* = 1 in $CHCl_3$)

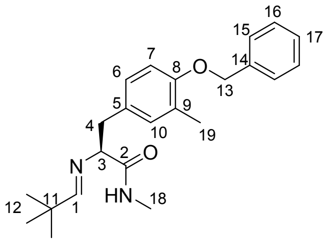

(S,E)-3-(4-(benzyloxy)-3-methylphenyl)-2-((2,2-dimethylpropylidene)amino)-N-methylpropanamide (S5)

To a solution of **S4** (3.00 g, 10.05 mmol) in dichloromethane (10 mL), $MgSO_4$ (3.00 g, 100% wt.) and pivaldehyde (1.31 mL, 12.07 mmol) was added. The mixture was stirred at room temperature overnight. The solution was filtered and washed with dichloromethane (3 x 30 mL). The filtrate was concentrated *in vacuo* to yield **S5** (3.68 g, Quant.) as a white powder.

^{1}H NMR (400 MHz, Chloroform-d) δ 7.45 – 7.40 (m, 2H, H_{15}), 7.40 – 7.34 (m, 2H, H_{16}), 7.33 – 7.28 (m, 1H, H_{17}), 6.91 (d, J = 5.0 Hz, 1H, N-H), 6.86-6.79 (m, 3H, $H_{1,7,10}$), 6.75 (d, J = 8.0 Hz, 1H, H_6), 5.05 (s, 2H, H_{13}), 3.63 (dd, J = 10.5, 3.0 Hz, 1H, H_3), 3.26 (dd, J = 13.5, 3.0 Hz, 1H, H_4), 2.84 (d, J = 5.0 Hz, 3H, H_{18}), 2.64 (dd, J = 13.5, 10.5 Hz, 1H, H_4), 2.23 (s, 3H, H_{19}), 0.89 (s, 9H, H_{12})

^{13}C NMR (100 MHz, Chloroform-d) δ 174.26 (C_1), 173.72 (C_2), 155.52 (C_8), 137.64 (C_{14}), 132.76 (C_{10}), 129.73 (C_5), 128.60 (C_{16}), 128.23 ($C_{7,1}$), 127.81 (C_{17}), 127.12 (C_{15}), 126.71 (C_9), 111.44 (C_6), 74.67 (C_3), 70.00 (C_{13}), 40.23 (C_4), 36.38 (C_{11}), 26.79 (C_{12}), 26.01 (C_{18}), 16.49 (C_{19})

HRMS-ESI (*m/z*) $[M+H]^+$ calc. for $C_{23}H_{31}N_2O_2$, 367.2380; found 367.2396

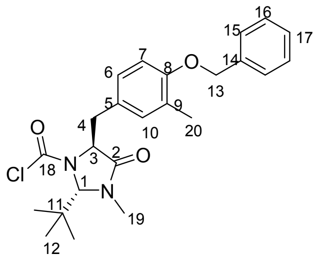

(5*S*)-5-(4-(benzyloxy)-3-methylbenzyl)-2-(*tert*-butyl)-3-methyl-4-oxoimidazolidine-1-carbonyl chloride (S6)

To a solution of **S5** (3.68 g, 10.05 mmol) in THF (20 mL), phosgene (10.78 mL, 15%, 15.08 mmol) was added. The mixture was stirred at room temperature for 2 hours. Pyridine (1.63 mL, 20.10 mmol) was then added and the mixture stirred for a further 2 hour. The solution was concentrated *in vacuo* and redissolved in dichloromethane (50 mL). The organic phase was washed with HCl (1M, 3 x 30 mL) and brine (20 mL), then dried over $MgSO_4$ and concentrated in *vacuo*. Purification was carried out by column chromatography on silica gel eluting with a gradient of 0-100% EtOAc in hexane to yield **S6** (3.46 g, 80 %) as a yellow oil.

NMR reported as a rotameric mixture

^{1}H NMR (400 MHz, Chloroform-d) δ 7.43 (d, J = 7.0 Hz, 2H, H_{15}), 7.41 – 7.35 (m, 2H, H_{16}), 7.33 – 7.29 (m, 1H, H_{17}), 7.00-6.90 (m, 2H, $H_{6\&10}$), 6.76 (d, J = 8.3 Hz, 1H, H_7), 5.04 (s, 2H, H_{13}), 4.72 (s, 0.54H, H_1), 4.60 (s, 0.46H, H_1), 4.41 (d, J = 5.0 Hz, 0.54H, H_3), 4.37 (d, J = 5.0 Hz, 0.46H, H_3), 3.78 (dd, J = 14.6, 5.0 Hz, 0.46H, H_4), 3.65 (dd, J = 14.6, 5.0 Hz, 0.54H, H_4), 3.27 (d, J = 14.6 Hz, 0.46H, H_4), 3.18 (d, J = 14.6 Hz, 0.54H, H_4), 2.86 (s, 1.62H, H_{19}), 2.75 (s, 1.38H, H_{19}), 2.23 (s, 3H, H_{20}), 1.00 (s, 4.86H, H_{12}), 0.93 (s, 4.14H, H_{12})

^{13}C NMR (125 MHz, Chloroform-d) δ 170.05 (C_2), 169.64 (C_2), 156.24 (C_8), 156.02 (C_8), 147.42 (C_{18}), 146.24 (C_{18}), 137.55 (C_{14}), 137.46 (C_{14}), 132.48 (C_{10}), 132.26 (C_{10}), 128.58 ($C_{16\&6}$), 127.85 (C_{17}), 127.27 (C_{15}), 126.19 (C_5), 125.80 (C_5), 111.31 (C_7), 83.49 (C_1), 82.90 (C_1), 69.89 (C_{13}), 63.89 (C_3), 62.87 (C_3), 41.80 (C_{11}), 41.70 (C_{11}), 35.92 (C_4), 32.14 (C_{19}), 32.09 (C_4), 26.79 (C_{12}), 26.54 (C_{12}), 16.34 (C_{20})

HRMS-ESI (*m/z*) $[M+H]^+$ calc. for $C_{24}H_{29}N_2O_3Cl$, 429.1939; found 429.1924

IR vmax – 2963, 2931 (C-H), 1739, 1708 (C=O)

R_f - 0.59 in 50% EtOAc in Hexane

mp 92-94 °C

[α] $^{26}_{D}$ = 4 ° (*c* = 1 in $CHCl_3$)

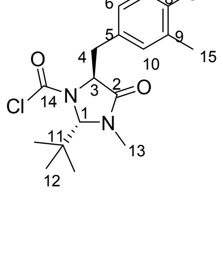

(5*S*)-2-(*tert*-butyl)-5-(4-hydroxy-3-methylbenzyl)-3-methyl-4-oxoimidazolidine-1-carbonyl chloride (22)

To a solution of **S6** (3.35 g, 7.82 mmol) in THF (39 mL) under N_2, palladium on active carbon (10 wt. %) (670 mg, 20 % wt.) was added. The mixture was then stirred at room temperature for 2 hours under hydrogen atmosphere. The reaction mixture was filtered over a pad of celite and concentrated in *vacuo* to yield **22** (2.61 g, 94 %) as a white powder.

^{1}H NMR (500 MHz, Chloroform-d) δ 6.93 (s, 1H, H_{10}), 6.87 (d, J = 8.2 Hz, 1H, $H_{6/7}$), 6.65 (t, J = 8.6 Hz, 1H, $H_{6/7}$), 4.73 (s, 0.55H, H_1), 4.62 (s, 0.45H, H_1) 4.44 – 4.34 (m, 1H, H_3), 3.84 – 3.72 (m, 0.55H, H_4), 3.64 (dd, J = 14.6, 5.2 Hz, 0.45H, H_4) 3.26 (d, J = 14.6 Hz, 0.45H, H_4), 3.15 (d, J = 14.6 Hz, 0.55H, H_4), 2.88 (s, 1.65H, H_{13}), 2.77 (s, 1.35H, H_{13}), 2.19 (s, 3H, H_{15}), 1.00 (s, 4.95H, H_{12}), 0.93 (s, 4.05H, H_{12}).

^{13}C NMR (126 MHz, Chloroform-d) δ 170.19 (C_2), 169.77 (C_2), 153.47 (C_8), 153.14 (C_8), 147.54 (C_{14}), 146.31 (C_{14}), 132.83 (C_{10}), 132.61 (C_{10}), 128.88 ($C_{6/7}$), 128.73 ($C_{6/7}$), 126.16 (C_5), 125.77 (C_5), 124.02 (C_9), 123.71 (C_9), 114.95 ($C_{6/7}$), 114.82 ($C_{6/7}$), 83.59 (C_1), 82.99 (C_1), 63.95 (C_3), 62.95 (C_3), 41.83 (C_{11}), 41.71 (C_{11}), 35.87 (C_4), 32.19 (C_4), 32.04 (C_{13}), 26.80 (C_{12}), 26.55 (C_{12}), 15.75 (C_{15}).

HRMS-ESI (*m/z*) $[M+H-H_2O]^+$ calc. for $C_{17}H_{23}N_2O_3$, 303.1703; found 303.1703

IR vmax – 3363 (O-H), 2968, 2875 (C-H), 1738, 1691 (C=O)

R_f - 0.46 in 10% MeOH in Dichloromethane

mp 134-135 °C

$[\alpha]_D^{26}$ = -12 ° (*c* = 1 in $CHCl_3$)

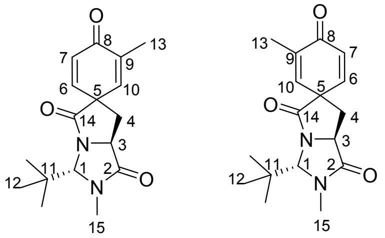

(7a'S)-3'-(tert-butyl)-2',3-dimethyl-2',3',7',7a'-tetrahydro-1'H,5'H-spiro[cyclohexane-1,6'-pyrrolo[1,2-c]imidazole]-2,5-diene-1',4,5'-trione (23)

To a solution of **22** (1.1 g, 3.08 mmol) in MeCN (4.4 mL), triethylamine (0.46 mL, 3.39 mmol) was added. The mixture was then heated under microwave irradiation at 150 °C for 10 minutes. The reaction mixture was concentrated *in vacuo*, redissolved in dichloromethane (20 mL) and washed with water (20 mL). The aqueous phase was extracted with dichloromethane (3 x 20 mL). The organic layer was washed with brine (10 mL), dried over $MgSO_4$ and concentrated in *vacuo* to yield **23** (926 mg, Quant., 1:1) as a white powder.

^{1}H NMR (400 MHz, Chloroform-d) δ 6.90 (dd, J = 9.8, 3.0 Hz, 0.5H, H_6), 6.71 – 6.65 (m, 1H, $H_{6\&10}$), 6.52 – 6.44 (m, 1H, $H_{7\&10}$), 6.40 (d, J = 9.8 Hz, 0.5H, H_7), 4.85 (t, J = 1.4 Hz, 1H, H_1), 4.45 (q, J = 8.2 Hz, 1H, H_3), 3.04 (d, J = 1.2 Hz,

3H, H_{15}), 2.59 (ddd, J = 13.2, 7.4, 3.5 Hz, 1H, H_4), 2.44 (ddd, J = 13.2, 9.0, 1.1 Hz, 1H, H_4), 1.96 (t, J = 1.4 Hz, 3H, H_{13}), 1.05 (s, 4.5H, H_{12}), 1.04 (s, 4.5H, H_{12}) - as 1:1 mix of diastereomers

^{13}C NMR (100 MHz, Chloroform-d) δ 185.60 (C_8), 185.50 (C_8), 174.87 (C_2), 174.72 (C_2), 171.70 (C_{14}), 171.68 (C_4), 146.08 ($C_{6/10}$), 143.37 (C_6), 141.70 ($C_{7/10}$), 139.22 (C_9), 138.81 ($C_{6/10}$), 137.97 (C_9), 132.08 ($C_{7/10}$), 130.73 (C_7), 82.03 (C_1), 82.01 (C_1), 57.13 (C_3), 56.94 (C_3), 54.16 (C_5), 54.05 (C_5), 38.53 (C_{11}), 37.65 (C_4), 37.58 (C_4), 31.61 (C_{15}), 31.60 (C_{15}), 25.98 (C_{12}), 25.95 (C_{12}), 16.34 (C_{13}), 16.31 (C_{13}).

HRMS-ESI (*m/z*) $[M+Na]^+$ calc. for $C_{17}H_{22}N_2O_3Na$, 325.1523; found 325.1515.

R_f – 0.28/0.34 in 5% MeOH in dichloromethane.

The diastereomers could be separated by column chromatography on silica gel eluting with a gradient of 0-30% Acetone in dichloromethane for testing purposes, otherwise material was taken through crude.

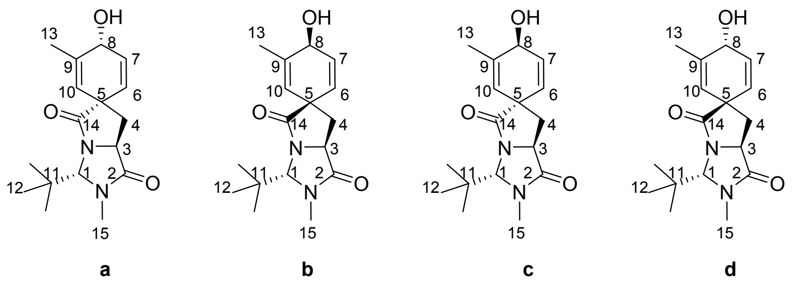

(1R,4S,7a'S)-3'-(tert-butyl)-4-hydroxy-2',3-dimethyl-2',3',7',7a'-tetrahydro-1'H,5'H-spiro[cyclohexane-1,6'-pyrrolo[1,2-c]imidazole]-2,5-diene-1',5'-dione & diastereomers (24)

To a solution of **23** (0.93 g, 3.08 mmol) in dry methanol (30 mL) under N_2, cerium(III) chloride heptahydrate (175 mg, 4.62 mmol) was added and stirred for 15 minutes at room temperature. The reaction mixture was then cooled to -78 °C and sodium borohydride (1.72 g, 4.62 mmol) was added. The resultant mixture was left to stir for a further 30 minutes and then quenched with water (10 mL). The mixture was then diluted with EtOAc (50 mL) and washed with water (30 mL). The aqueous phase was extracted with EtOAc (3 x 30 mL). The combined organics were washed with brine (30 mL), dried over $MgSO_4$, filtered and concentrated *in vacuo*. Purification was carried out by column chromatography on silica gel eluting with 0-10% MeOH in dichloromethane to yield **24** (615 mg, 66 %, d.r. = 1.5:1.5:1.2:1) white powders.

<u>**24a**</u>

(177 mg, 18%)

^{1}H NMR (500 MHz, Chloroform-d) δ 6.12 (dd, J = 9.7, 4.2 Hz, 1H, H_7), 5.92 (dd, J = 9.7, 1.6 Hz, 1H, H_6), 5.34-5.30 (m, 1H, H_{10}), 4.77 (d, J = 1.1 Hz, 1H, H_1), 4.34 (t, J = 8.2 Hz, 1H, H_3), 4.27 (dd, J = 11.1, 4.2 Hz, 1H, H_8), 3.00 (s, 3H, H_{15}), 2.47 (dd, J = 13.2, 7.8 Hz, 1H, H_4), 2.13 (dd, J = 13.2, 8.6 Hz, 1H, H_4), 1.97 (d, J = 11.2 Hz, 3H, O-H), 1.93 (d, J = 1.5 Hz, 1H, H_{13}), 1.01 (s, 9H, H_{12})

^{13}C NMR (125 MHz, Chloroform-d) δ 178.52 (C_2), 172.58 (C_{14}), 139.69 (C_9), 130.70 (C_7), 128.86 (C_6), 125.25 (C_{10}), 82.05 (C_1), 65.29 (C_8), 56.92 (C_3), 51.71 (C_5), 38.98 (C_4), 38.50 (C_{11}), 31.49 (C_{15}), 25.99 (C_{12}), 20.43 (C_{13})

IR vmax – 3416 (O-H), 2965 (C-H), 1700 (C=O)

HRMS-ESI (*m/z*) $[M+Na]^+$ calc. for $C_{17}H_{24}N_2O_3Na$, 327.1679; found 327.1680

R_f – 0.38 in 10% MeOH in dichloromethane

mp 173-175 °C

[α] $_D^{26}$ = -4 ° (*c* = 1 in $CHCl_3$)

Carbonyl down confirmed by NOE (Doublet 'sees' alpha)

Major isomer assumed as *cis*

24b

(181 mg, 18%)

^{1}H NMR (500 MHz, Chloroform-d) δ 6.23 (dd, J = 9.7, 4.2 Hz, 1H, H_7), 5.66 – 5.57 (m, 2H, $H_{6\&9}$), 4.78 (d, J = 1.1 Hz, 1H, H_1), 4.36 (t, J = 8.2 Hz, 1H, H_3), 4.27 (dd, J = 11.2, 4.2 Hz, 1H, H_8), 3.00 (d, J = 0.4 Hz, 3H, H_{15}), 2.46 (dd, J = 13.2, 7.8 Hz, 1H, H_4), 2.16 (dd, J = 13.2, 8.6 Hz, 1H, H_4), 1.92 (d, J = 1.2 Hz, 3H, H_{13}), 1.80 (d, J = 11.2 Hz, 1H, O-H) 1.03 (s, 9H, H_{12})

^{13}C NMR (125 MHz, Chloroform-d) δ 178.66 (C_2), 172.59 (C_{14}), 138.65 (C_9), 131.71 (C_7), 129.86 (C_6), 123.34 (C_{10}), 82.00 (C_1), 65.24 (C_8), 56.97 (C_3), 51.46 (C_5), 38.94 (C_4), 38.49 (C_{11}), 31.51 (C_{15}), 26.01 (C_{12}), 20.42 (C_{13})

IR vmax – 3416 (O-H), 2966 (C-H), 1698 (C=O)

HRMS-ESI (*m/z*) $[M+Na]^+$ calc. for $C_{17}H_{24}N_2O_3Na$, 327.1679; found 327.1683

R_f – 0.35 in 10% MeOH in dichloromethane

mp 56-58 °C

[α] $_D^{26}$ = -8 ° (*c* = 1 in $CHCl_3$)

Carbonyl up confirmed by NOE (Singlet 'sees' alpha)

Major isomer assumed as *cis*

24c

(137 mg, 14%)

^{1}H NMR (500 MHz, Chloroform-d) δ 6.03 (dd, J = 9.8, 3.2 Hz, 1H, H_7), 5.88 (dt, J = 9.8, 1.9 Hz, 1H, H_6), 5.34 – 5.29 (m, 1H, H_{10}), 4.79 (d, J = 1.1 Hz, 1H, H_1), 4.49 (d, J = 8.5 Hz, 1H, H_8), 4.33 (t, J = 8.2 Hz, 1H, H_3), 2.99 (s, 3H , H_{15}), 2.45 (dd, J = 13.1, 7.5 Hz, 1H, H_4), 2.17 (dd, J = 13.1, 9.0 Hz, 1H, H_4), 1.93 (s, 3H, H_{13}), 1.56 (d, J = 9.1 Hz, 1H, O-H), 1.01 (s, 9H, H_{12})

^{13}C NMR (125 MHz, Chloroform-d) δ 178.31 (C_2), 172.59 (C_{14}), 138.27 (C_9), 129.98 (C_7), 126.70 (C_6), 123.73 (C_{10}), 81.73 (C_1), 65.31 (C_8), 56.81 (C_3), 51.32 (C_5), 38.95 (C_4), 38.49 (C_{11}), 31.48 (C_{15}), 25.96 (C_{12}), 20.15 (C_{13})

IR vmax – 3417 (O-H), 2966 (C-H), 1697 (C=O)

HRMS-ESI (*m/z*) $[M+Na]^+$ calc. for $C_{17}H_{24}N_2O_3Na$, 327.1679; found 327.1690

R_f – 0.32 in 10% MeOH in dichloromethane

mp >200 °C decomposition

[α]$_D^{26}$ = 20 ° (*c* = 1 in $CHCl_3$)

Carbonyl down confirmed by NOE (Doublet 'sees' alpha)

Minor isomer assumed as *trans*

<u>24d</u>

(120 mg, 12%)

^{1}H NMR (500 MHz, Chloroform-d) δ 6.14 (dd, J = 9.6, 3.3 Hz, 1H, H_7), 5.61 – 5.54 (m, 2H, $H_{6\&10}$), 4.79 (d, J = 1.2 Hz, 1H, H_1), 4.48 (s, br, 1H, H_8), 4.35 (t, J = 8.3 Hz, 1H, H_3), 3.00 (s, 3H, H_{15}), 2.45 (dd, J = 13.0, 7.5 Hz, 1H, H_4), 2.16 (dd, *J* = 13.0, 8.9 Hz, 1H, H_4), 1.90 (s, 3H, H_{13}), 1.55 (s, 1H, O-H), 1.02 (s, 9H, H_{12})

^{13}C NMR (125 MHz, Chloroform-d) δ 178.42 (C_2), 172.62 (C_{14}), 136.82 (C_9), 131.55 (C_7), 128.42 (C_6), 121.92 (C_{10}), 81.72 (C_1), 65.26 (C_8), 57.05 (C_3), 51.35 (C_5), 39.09 (C_4), 38.48 (C_{11}), 31.50 (C_{15}), 25.99 (C_{12}), 20.17 (C_{13})

IR vmax – 3425 (O-H), 2967 (C-H), 1698 (C=O)

HRMS-ESI (*m/z*) $[M+Na]^+$ calc. for $C_{17}H_{24}N_2O_3Na$, 327.1679; found 327.1676

R_f – 0.30 in 10% MeOH in dichloromethane

mp 160-162 °C

[α]$_D^{26}$ = -20 ° (*c* = 1 in $CHCl_3$)

Carbonyl up confirmed by NOE (Singlet 'sees' alpha)

Major isomer assumed as *trans* & confirmed by crystal below

Table 3 Crystal data and structure refinement for 24d.

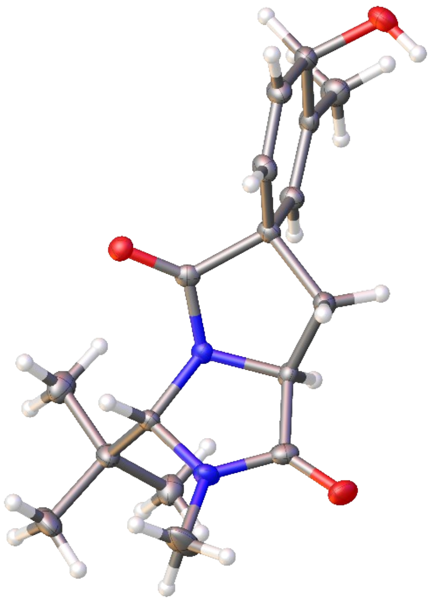

CCDC deposition number	2070024
Empirical formula	$C_{17}H_{24}N_2O_3$
Formula weight	304.38
Temperature/K	100(2)
Crystal system	monoclinic
Space group	$P2_1$
a/Å	5.7444(2)
b/Å	12.0901(5)
c/Å	12.1546(6)
α/°	90
β/°	95.471(3)
γ/°	90
Volume/Å^3	840.30(6)
Z	2
ρ_{calc}g/cm^3	1.203
μ/mm^{-1}	0.083
F(000)	328.0
Crystal size/mm^3	0.32 × 0.24 × 0.2
Radiation	MoKα (λ = 0.71073)
2Θ range for data collection/°	4.762 to 54.164
Index ranges	-7 ≤ h ≤ 7, -8 ≤ k ≤ 15, -15 ≤ l ≤ 15
Reflections collected	6822
Independent reflections	3061 [R_{int} = 0.0355, R_{sigma} = 0.0476]
Data/restraints/parameters	3061/1/205
Goodness-of-fit on F^2	1.021
Final R indexes [I>=2σ (I)]	R_1 = 0.0417, wR_2 = 0.0841
Final R indexes [all data]	R_1 = 0.0544, wR_2 = 0.0903
Largest diff. peak/hole / e Å^{-3}	0.21/-0.21

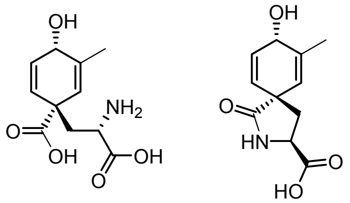

(1s,4*R*)-1-((*S*)-2-amino-2-carboxyethyl)-4-hydroxy-3-methylcyclohexa-2,5-diene-1-carboxylic acid (26) & (3*S*,5r,8*S*)-8-hydroxy-7-methyl-1-oxo-2-azaspiro[4.5]deca-6,9-diene-3-carboxylic acid (25)

To a solution of **24a** (150.9 mg, 0.50 mmol) in water (2.5 mL), barium hydroxide (255 mg, 1.49 mmol) and barium carbonate (98 mg, 0.50 mmol) was added. The mixture was stirred at 40 °C for 16 hours, then sodium carbonate anhydrous (315 mg, 2.98 mmol) and water (15 mL) were added. The resulting barium carbonate salt was filtered through a Whatman 0.45 µm Nylon syringe filter. The aqueous phase was lyophilized. The solid was

redissolved in water (15 mL), filtered through a Millipore Millex-HN 0.45 µm Nylon syringe filter and purified by Hypercarb HPLC with a gradient of 0-80% acetonitrile in 10 mM sodium carbonate-bicarbonate buffer (pH 9.2) to yield:

26 as a mixture of salts (14 % by quantitative NMR using a stock solution of EtOH) as a white powder.

25 as the sodium salt (29 % by quantitative NMR using a stock solution of EtOH) as a white powder.

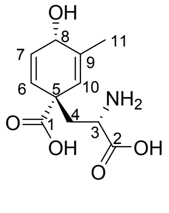

(1s,4*R*)-1-((*S*)-2-amino-2-carboxyethyl)-4-hydroxy-3-methylcyclohexa-2,5-diene-1-carboxylic acid (26)

^{1}H NMR (500 MHz, Deuterium Oxide) δ 5.86 – 5.63 (m, 2H, $H_{6\&7}$), 5.46 (s, 0.8H, H_{10}), 5.41 (s, 0.2H, H_{10}), 4.27 – 4.25 (m, 0.2H, H_8), 4.25 – 4.21 (m, 0.8H, H_8), 3.55 (dd, J = 10.4, 3.1 Hz, 0.8H, H_3), 2.88 (t, J = 6.0 Hz, 0.2H, H_3), 1.89 – 1.70 (m, 2H, H_4), 1.67 (s, 2.4H, H_{11}), 1,64 (s, 0.6H, H_{11}). 80:20 mixture of salts.

^{13}C NMR (126 MHz, Deuterium Oxide) δ 188.83 (C_1), 188.09 (C_2), 141.15 (C_8), 138.52 ($C_{6/7}$), 133.68 ($C_{6/7}$), 132.91 (C_{10}), 71.43 (C_8), 61.21 (C_3), 57.54 (C_5), 47.79 (C_4), 25.85 (C_{11}).

HRMS-Nanospray (*m/z*) $[M-H]^-$ calc. for $C_{11}H_{14}NO_5$, 240.0872; found 240.0879.

[α] $_D^{23}$ = -80 ° (*c* = 0.1 in H_2O)

CD spectra – Positive Cotton effect below 230 nm

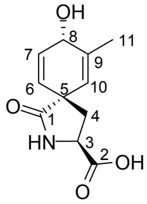

(3*S*,5r,8*S*)-8-hydroxy-7-methyl-1-oxo-2-azaspiro[4.5]deca-6,9-diene-3-carboxylic acid (25)

^{1}H NMR (500 MHz, Deuterium Oxide) δ 5.98 (dd, J = 9.9, 3.3 Hz, 1H, H_7), 5.79 (m, 1H, H_6), 5.48 – 5.41 (m, 1H, H_{10}), 4.34 (d, J = 3.3 Hz, 1H, H_8), 4.17 (dd, J = 8.6, 6.5 Hz, 1H, H_3), 2.43 (dd, J = 13.3, 8.6 Hz, 1H, H_4), 2.01 (dd, J = 13.3, 6.5 Hz, 1H, H_4), 1.82 – 1.69 (m, 3H, H_{11}).

^{13}C NMR (126 MHz, Deuterium Oxide) δ 188.31 ($C_{1/2}$), 187.72 ($C_{1/2}$), 145.06 (C_9), 137.33 (C_7), 136.60 (C_6), 132.74 (C_{10}) 72.24 (C_8), 63.11 (C_3), 56.94 (C_5), 47.79 (C_4), 27.22 (C_{11}).

HRMS-ESI (*m/z*) $[M+Na+H]^+$ calc. for $C_{11}H_{13}NO_4$, 246.0737; found 246.0739.

$[\alpha]_D^{23}$ = 40 ° (*c* = 0.1 in H_2O)

CD spectra – Positive Cotton effect below 230 nm

2.2.2. Grignard addition

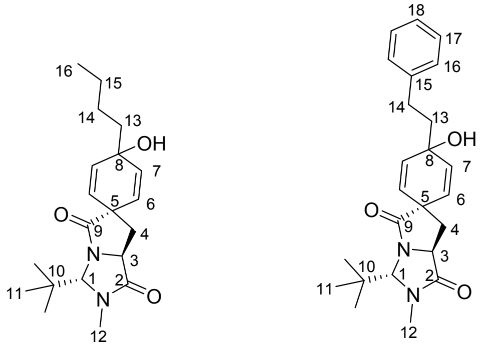

General procedure for Grignard addition

To a solution of **7** (1 eq.) in dry THF (0.1 M) under N_2 at -78 °C, the relevant Grignard reagent (2M in THF, 1.5 eq.) was slowly added and stirred for 2 hours at room temperature. The reaction mixture was then cooled to 0 °C and quenched slowly with water. The mixture was concentrated *in vacuo*, diluted with water and extracted with ether. Purification was carried out by column chromatography on silica gel eluting with 0-100% EtOAc in Hexane to yield the corresponding alkylated product.

(7a'*S*)-3'-(*tert*-butyl)-4-butyl-4-hydroxy-2'-methyl-2',3',7',7a'-tetrahydro-1'H,5'H-spiro[cyclohexane-1,6'-pyrrolo[1,2-c]imidazole]-2,5-diene-1',5'-dione (16)

Only one diastereomer isolated (crude NMR d.r. = >9:1)

Major isolated diastereomer (441 mg, 75 %) as a pale yellow oil

^{1}H NMR (400 MHz, Chloroform-d) δ 6.00 (dd, J = 10.0, 1.5 Hz, 1H, H_6), 5.94 – 5.85 (m, 2H, H_7), 5.62 (dd, J = 10.0, 1.5 Hz, 1H, H_6), 4.79 (d, J = 1.1 Hz, 1H, H_1), 4.35 (t, J = 8.2 Hz, 1H, H_3), 3.00 (s, 3H, H_{12}), 2.42 (dd, J = 13.2, 7.7 Hz, 1H, H_4), 2.25 – 2.13 (m, 2H, H_4 & O-H), 1.66 – 1.56 (m, 2H, H_{13}), 1.36 – 1.24 (m, 2H, H_{15}), 1.15 – 1.05 (m, 2H, H_{14}), 1.02 (s, 9H, H_{11}), 0.87 (t, J = 7.3 Hz, 3H, H_{16})

^{13}C NMR (100 MHz, Chloroform-d) δ 178.21 (C_2), 172.46 (C_9), 135.63 (C_6), 134.79 (C_7), 128.70 (C_6), 127.33 (C_7), 81.90 (C_1), 67.69 (C_8), 56.99 (C_3), 50.86 (C_5), 39.93 (C_{13}), 38.97 (C_4), 38.52 (C_{10}), 31.51 (C_{12}), 26.69 (C_{14}), 25.99 (C_{11}), 23.09 (C_{15}), 14.16 (C_{16})

HRMS-ESI (*m/z*) $[M+Na^+]$ calc. for $C_{20}H_{30}N_2O_3Na$, 369.2149; found 369.2164

IR vmax – 3416 (O-H), 2957, 2931 (C–H), 1697 (C=O)

R_f - 0.47 in 10% MeOH in dichloromethane

$[\alpha]^{25}_{D}$ = 8° (*c* = 1 in $CHCl_3$)

(7a'*S*)-3'-(*tert*-butyl)-4-hydroxy-2'-methyl-4-phenethyl-2',3',7',7a'-tetrahydro-1'H,5'H-spiro[cyclohexane-1,6'-pyrrolo[1,2-c]imidazole]-2,5-diene-1',5'-dione (17)

Only one diastereomer isolated (crude NMR d.r. = >9:1)

Major isolated diastereomer (202 mg, 74 %) as a colourless solid

^{1}H NMR (500 MHz, Chloroform-d) δ 7.31 – 7.23 (m, 2H, H_{17}), 7.20 – 7.13 (m, 3H, $H_{16,18}$), 6.09 (dd, J = 9.9, 1.9 Hz, 1H, H_7), 6.00 (dd, J = 9.8, 1.9 Hz, 1H, H_7), 5.94 (dd, J = 9.8, 2.2 Hz, 1H, H_6), 5.68 (dd, J = 9.9, 2.2 Hz, 1H, H_6), 4.80 (s, 1H, H_1), 4.38 (t, J = 8.2 Hz, 1H, H_3), 3.01 (s, 3H, H_{12}), 2.54 – 2.38 (m, 4H, $H_{4,14}$ & O-H), 2.21 (dd, J = 13.2, 8.7 Hz, 1H, H_4), 1.99 – 1.91 (m, 2H, H_{13}), 1.03 (s, 9H, H_{11})

^{13}C NMR (100 MHz, Chloroform-d) δ 178.01 (C_2), 172.40 (C_9), 141.84 (C_{15}), 135.26 (C_7), 134.37 (C_7), 129.35 (C_6), 128.53 (C_{17}), 128.40 (C_{16}), 127.92 (C_6), 126.02 (C_{18}), 81.97 (C_1), 67.46 (C_8), 57.00 (C_3), 50.99 (C_5), 41.79 (C_{13}), 38.81 (C_4), 38.81 (C_{10}), 31.53 (C_{12}), 30.77 (C_{14}), 26.01 (C_{11})

HRMS-ESI (*m/z*) [M+Na$^+$] calc. for $C_{24}H_{30}N_2O_3Na$, 417.2149; found 417.2152

IR vmax – 3405 (O-H), 2964, 2931 (C–H), 1693 (C=O)

R_f - 0.53 in 10% MeOH in dichloromethane

mp 139-141 °C

$[\alpha]^{25}_{D}$ = 16° (*c* = 1 in $CHCl_3$)

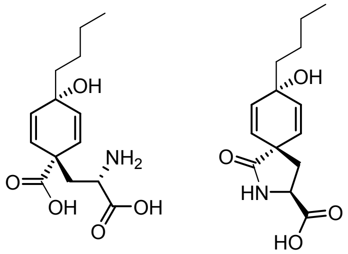

(1s,4S)-1-((R)-2-amino-2-carboxyethyl)-4-hydroxy-4-butylcyclohexa-2,5-diene-1-carboxylic acid (17) & (3S,5s,8R)-8-hydroxy-1-oxo-8-butyl-2-azaspiro[4.5]deca-6,9-diene-3-carboxylic acid (S7)

To a solution of **16** (26.0 mg, 0.075 mmol), sodium hydroxide (18 mg, 0.45 mmol) and sodium carbonate (7.9 mg, 0.075 mmol) were dissolved in ethanol (0.167 mL) and H_2O (0.083 mL). The mixture was stirred at 70 °C for 20 hours and concentrated *in vacuo*. The solid was redissolved in water (1.2 mL), filtered through a Millipore Millex-HN 0.45 µm Nylon syringe filter and purified by Hypercarb HPLC with a gradient of 0-80% acetonitrile in 10 mM sodium carbonate-bicarbonate buffer (pH 9.2) to yield:

18 as a mixture of salts (51 % by quantitative NMR using a stock solution of EtOH) as a white powder.

S7 as the sodium salt (26 % by quantitative NMR using a stock solution of EtOH) as a white powder.

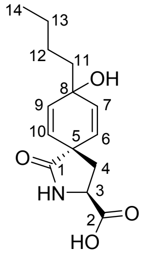

(3S,5s,8R)-8-hydroxy-1-oxo-8-butyl-2-azaspiro[4.5]deca-6,9-diene-3-carboxylic acid (S7)

^{1}H NMR (500 MHz, Deuterium Oxide) δ 5.78 – 5.70 (m, 3H, $H_{6/10}$), 5.67 (dd, J = 10.2, 2.1 Hz, 1H), 4.13 (dd, J = 8.7, 6.2 Hz, 1H), 2.39 (dd, J = 13.3, 8.7 Hz, 1H), 1.99 (dd, J = 13.3, 6.2 Hz, 1H), 1.48 – 1.40 (m, 2H), 1.13 (h, J = 7.4 Hz, 2H), 0.98 – 0.88 (m, 2H), 0.70 (t, J = 7.4 Hz, 3H).

^{13}C NMR (126 MHz, Deuterium Oxide) δ 188.47 ($C_{1/2}$), 188.08 ($C_{1/2}$), 141.17 ($C_{6/10}$), 141.09 ($C_{6/10}$), 136.91 ($C_{7/9}$), 136.33 ($C_{7/9}$), 76.49 (C_8), 63.55 (C_3), 56.48 (C_5), 47.87 (C_{11}), 47.66 (C_4), 34.34 (C_{12}), 30.59 (C_{12}), 21.56 (C_{13}).

HRMS-Nanospray (*m/z*) $[M-H]^-$ calc. for $C_{14}H_{18}NO_4$, 264.1236; found 264.1227

[α] $^{25}_{D}$ = 80 ° (*c* = 0.1 in H_2O)

CD spectra – Positive Cotton effect below 230 nm

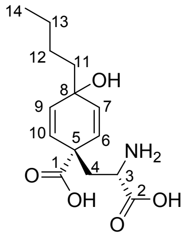

(1s,4S)-1-((R)-2-amino-2-carboxyethyl)-4-hydroxy-4-butylcyclohexa-2,5-diene-1-carboxylic acid (17)

^{1}H NMR (500 MHz, Deuterium Oxide) δ 6.09 (dd, *J* = 10.4, 2.3 Hz, 0.4H, $H_{7/9}$), 6.04 (dd, *J* = 10.2, 2.3 Hz, 0.6H, $H_{7/9}$), 5.98 – 5.92 (m, 1H, $H_{7/9}$), 5.91 – 5.76 (m, 2H, $H_{6\&10}$), 3.78 (dd, *J* = 10.9, 3.1 Hz, 0.6H, H_3), 3.30 (dd, *J* = 8.7, 2.8 Hz, 0.4H, H_3), 2.19 (dd, *J* = 14.6, 2.8 Hz, 0.4H, H_4), 2.09 – 1.94 (m, 1.6H, H_4), 1.65 – 1.55 (m, 2H, H_{11}), 1.29 (h, *J* = 7.0 Hz, 2H, H_{13}), 1.25 – 1.14 (m, 2H, H_{12}), 0.86 (td, *J* = 7.2, 1.5 Hz, 3H, H_{14}).

^{13}C NMR (126 MHz, Deuterium Oxide) δ 182.6(C_2), 181.1(C_1), 180.6(C_1), 179.5(C_2), 163.3(CO_3^{2-}), 162.7(CO), 131.9 ($C_{6/10}$), 131.8 ($C_{6/10}$), 131.5 ($C_{6/10}$), 131.4 ($C_{6/10}$), 131.3 ($C_{7/9}$), 131.1 ($C_{7/9}$), 130.6 ($C_{7/9}$), 130.0 ($C_{7/9}$), 68.4 (C_8), 68.1 (C_8), 55.0 (C_3), 53.1 (C_3), 50.9 (C_5), 50.5 (C_5), 42.8 (C_4), 41.2 (C_4), 40.3 ($2 \times C_{11}$), 25.9 (C_{12}), 25.5 (C_{12}), 22.4 (C_{13}), 22.4 (C_{13}), 13.2 (C_{14}), 13.2 (C_{14}).

HRMS-Nanospray (*m/z*) $[M-H]^-$ calc. for $C_{14}H_{20}NO_5$, 282.1341; found 282.1336

$[\alpha]_D^{24}$ = 80 ° (*c* = 0.1 in H_2O)

CD spectra – Positive Cotton effect below 230 nm

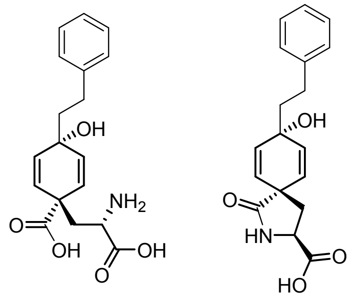

(1s,4S)-1-((R)-2-amino-2-carboxyethyl)-4-hydroxy-4-phenethylcyclohexa-2,5-diene-1-carboxylic acid (18) & (3S,5s,8R)-8-hydroxy-1-oxo-8-phenethyl-2-azaspiro[4.5]deca-6,9-diene-3-carboxylic acid (S8)

To a solution of **16** (26.0 mg, 0.075 mmol), sodium hydroxide (18 mg, 0.45 mmol) and sodium carbonate (7.9 mg, 0.075 mmol) were dissolved in ethanol (0.167 mL) and H_2O (0.083 mL). The mixture was stirred at 70 °C for 20 hours and concentrated *in vacuo*. The solid was redissolved in water (1.2 mL), filtered through a Millipore Millex-HN 0.45 µm Nylon syringe filter and purified by Hypercarb HPLC with a gradient of 0-80% acetonitrile in 10 mM sodium carbonate-bicarbonate buffer (pH 9.2) to yield:

19 as a mixture of salts (57 % by quantitative NMR using a stock solution of EtOH) as a white powder.

S8 as the sodium salt (19 % by quantitative NMR using a stock solution of EtOH) as a white powder.

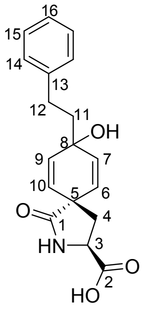

(3S,5s,8R)-8-hydroxy-1-oxo-8-phenethyl-2-azaspiro[4.5]deca-6,9-diene-3-carboxylic acid (S8)

^{1}H NMR (500 MHz, Deuterium Oxide) δ 7.38 – 7.31 (m, 2H, H_{15}), 7.28 – 7.20 (m, 3H, $H_{14\&16}$), 5.99 – 5.88 (m, 3H, $H_{9,7\&6/10}$), 5.84 (dd, J = 10.0, 2.3 Hz, 1H, $H_{6/10}$), 4.27 (dd, J = 8.7, 6.1 Hz, 1H, H_3), 2.56 (dd, J = 13.4, 8.7 Hz, 1H, H_4), 2.51 – 2.39 (m, 2H, H_{12}), 2.16 (dd, J = 13.4, 6.1 Hz, 1H, H_4), 1.95 – 1.85 (m, 2H, H_{11}).

^{13}C NMR (126 MHz, Deuterium Oxide) δ 188.06 (C_1), 187.79 (C_2), 150.38 (C_{13}), 140.47 ($C_{7/9}$), 140.40 ($C_{7/9}$), 137.19 ($C_{6/10}$), 136.74 (C_{15}), 136.62 ($C_{6/10}$), 136.57 (C_{14}), 134.12 (C_{16}), 75.99 (C_8), 63.31 (C_3), 56.28 (C_5), 49.74 (C_{11}), 47.35 (C_4), 38.37 (C_{12}).

HRMS-Nanospray (*m/z*) $[M-H]^-$ calc. for $C_{18}H_{18}NO_4$, 312.1236; found 312.1227

[α] $^{22}_{D}$ = 40 ° (*c* = 0.1 in H_2O)

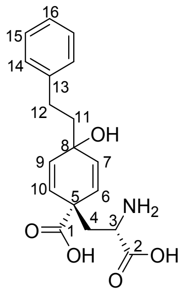

(1s,4S)-1-((R)-2-amino-2-carboxyethyl)-4-hydroxy-4-phenethylcyclohexa-2,5-diene-1-carboxylic acid (18)

^{1}H NMR (500 MHz, Deuterium Oxide) δ 7.40 – 7.22 (m, 5H, $H_{14,15\&16}$), 6.20 – 6.09 (m, 1H, $H_{7/9}$), 6.07 – 5.99 (m, 1H $H_{7/9}$), 5.98 – 5.82 (m, 2H $H_{6\&10}$), 3.83 (dd, *J* = 10.9, 3.1 Hz, 0.6H, H_3), 3.27 (dd, *J* = 8.8, 2.9 Hz, 0.4H, H_3), 2.64 – 2.50 (m, 2H H_{12}), 2.21 (dd, *J* = 14.3, 3.1 Hz, 0.4H, H_4), 2.14 – 1.96 (m, 1.6H, H_4), 1.94 – 1.82 (m, 2H, H_{11}).

^{13}C NMR (126 MHz, Deuterium Oxide) δ 182.5(C_2), 181.0(C_1), 180.6(C_2), 180.5(C_1), 163.8 (CO_3^{2-}), 163.3(CO), 142.8(C_{13}), 142.5(C_{13}), 132.6($C_{7/9}$), 132.5($C_{7/9}$), 132.3($C_{7/9}$), 132.1($C_{7/9}$), 130.6($C_{6/10}$), 130.5($C_{6/10}$), 130.0($C_{6/10}$), 129.5($C_{6/10}$), 128.7(C_{Ar}), 128.6(C_{Ar}), 128.5(C_{Ar}), 128.4(C_{Ar}), 126.0(C_{16}), 125.9(C_{16}), 68.2(C_8), 67.9(C_8), 55.0(C_3), 53.4(C_3), 51.0(C_5), 50.5(C_5), 43.4(C_4), 42.8(C_{11}), 42.7(C_{11}), 41.2(C_4), 30.1(C_{12}), 30.0(C_{12}).

HRMS-Nanospray (*m/z*) $[M-H]^-$ calc. for $C_{18}H_{20}NO_5$, 330.1341; found 330.1348

[α] $^{22}_{D}$ = 8 ° (*c* = 1 in H_2O)

2.2.3. Hydrogenation

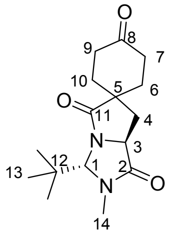

(7a'S)-3'-(tert-butyl)-2'-methyltetrahydro-1'H,5'H-spiro[cyclohexane-1,6'-pyrrolo[1,2-c]imidazole]-1',4,5'-trione (12)

To a solution of **7** (300 g, 1.04 mmol) in (10 mL) under N_2, palladium on active carbon (10 wt. %) (60 mg, 20 % wt.) was added. The mixture was then stirred at room temperature overnight under hydrogen atmosphere. The reaction mixture was filtered over a pad of celite and concentrated in *vacuo* to yield **12** (303 mg, Quant.) as a white powder.

^{1}H NMR (400 MHz, Chloroform-*d*) δ 4.82 (d, *J* = 1.1 Hz, 1H, H_1), 4.30 (t, *J* = 8.1 Hz, 1H, H_3), 3.03 (s, 3H, H_{12}), 2.96 – 2.85 (m, 1H, $H_{7/9}$), 2.61 – 2.48 (m, 2H, $H_{4\&7/9}$), 2.48 – 2.35 (m, 1H, $H_{7/9}$), 2.35 – 2.15 (m, 3H, $H_{6/10\&7/9}$), 2.10 – 1.93 (m, 2H, $H_{4\&6/10}$), 1.82 – 1.72 (m, 1H, $H_{6/10}$), 1.03 (s, 9H, H_{13})
^{13}C NMR (100 MHz, Chloroform-d) δ 209.60 (C_8), 181.50 (C_2), 172.69 (C_{11}), 81.49 (C_1), 56.68 (C_3), 45.85 (C_5), 38.37 (C_{12}), 37.71 ($C_{7/9}$), 37.06 (C_4), 33.56 ($C_{6/10}$), 33.29 ($C_{6/10}$), 31.48 (C_{12}), 25.97 (C_{14})
HRMS-ESI (*m/z*) [M+Na$^+$] calc. for $C_{16}H_{24}N_2O_3Na$, 315.1679; found 315.1689
IR vmax – 2962, 2871 (C–H), 1697 (C=O)
R_f - 0.35 in 100% EtOAc
mp 177-178 °C
[α] $^{26}_{D}$ = 4 ° (*c* = 1 in $CHCl_3$)

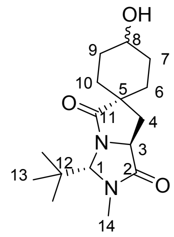

(3'R,7a'S)-3'-(tert-butyl)-4-hydroxy-2'-methyltetrahydro-1'H,5'H-spiro[cyclohexane-1,6'-pyrrolo[1,2-c]imidazole]-1',5'-dione (13)

Method 1:

To a solution of **12** (1.082 g, 3.7 mmol) in dry THF (55 mL) under N_2 at -78 °C, K-selectride (1M solution in THF) (5.92 mL, 5.92 mmol) was slowly added. The reaction mixture was stirred at -78 °C for one hour. To the reaction mixture was added H_2O (5 mL), MeOH (5 mL), NaOH (1M aq. solution, 5 mL) and H_2O_2 (30 % solution, 5 mL). The aqueous phase was extracted with dichloromethane (3 x 30 mL). The combined organic phase was dried over $MgSO_4$, filtered and concentrated *in vacuo*. Purification was carried out by column chromatography on silica gel eluting with 0-10% MeOH in dichloromethane to yield **13a** (1.01 g, 93 %) as a colourless solid.

Method 2:

To a solution of **12** (200 mg, 0.68 mmol) in dry methanol (7 mL) under N_2 at 0 °C, sodium borohydride (45 mg, 1.20 mmol) was slowly added. The resultant mixture was left to stir overnight and then quenched with water (20 mL). The mixture was then concentrated *in vacuo*, diluted with water (50 mL). The aqueous phase was extracted with dichloromethane (3 x 30 mL). The combined organic phase was dried over $MgSO_4$, filtered and concentrated *in vacuo*. Purification was carried out by column chromatography on silica gel eluting with 0-80% Acetone in dichloromethane to yield **13** (179 mg, 89 %, d.r. = 1:1.1 (**13a:13b**)) as white powders.

13a (*cis* – desired)

^{1}H NMR (500 MHz, Chloroform-d) δ 4.77 (d, J = 1.1 Hz, 1H, H_1), 4.21 (t, J = 8.2 Hz, 1H, H_3), 3.83 (dh, J = 7.1, 3.6 Hz, 1H, H_8), 2.96 (s, 3H, H_{14}), 2.40 (dd, J = 13.0, 7.8 Hz, 1H, H_4), 2.11 – 1.95 (m, 3H, $H_{6/10\&7/9}$), 1.87 – 1.74 (m, 2H, $H_{4\&7/9}$), 1.71 – 1.60 (m, 2H, $H_{7/9}$), 1.54-1.44 (m, 2H, $H_{6/10}$ & O-H), 1.32 – 1.23 (m, 1H, $H_{6/10}$), 1.00 (s, 9H, H_{13})

^{13}C NMR (125 MHz, Chloroform-d) δ 182.66 (C_2), 173.29 (C_{11}), 81.31 (C_1), 67.48 (C_8), 56.77 (C_3), 46.18 (C_5), 38.39 (C_{12}), 37.59 (C_4), 31.45 (C_{14}), 30.40 ($C_{7/9}$), 30.40 ($C_{7/9}$), 30.08 ($C_{6/10}$), 29.32 ($C_{6/10}$), 25.97 (C_{13})

HRMS-ESI (*m/z*) [M+Na$^+$] calc. for $C_{16}H_{26}N_2O_3Na$, 317.1836; found 317.1842

IR vmax – 3423 (O-H), 2955, 2929, 2870 (C–H), 1688 (C=O)

R_f - 0.35 in 10% MeOH in dichloromethane

mp 173-174 °C

[α] $^{26}_{D}$ = -20 ° (*c* = 1 in $CHCl_3$)

13b (*trans*)

^{1}H NMR (500 MHz, Chloroform-d) δ 4.76 (d, J = 1.2 Hz, 1H, H_1), 4.21 (t, J = 8.1 Hz, 1H, H_3), 3.67 (ddt, J = 14.3, 9.9, 4.1 Hz, H_8), 2.96 (s, 3H, H_{14}), 2.59 (dd, J = 13.2, 8. Hz, 1H, H_4), 2.06 - 1.92 (m, 2H, $H_{6/10\&7/9}$), 1.91 – 1.70 (m, 3H, $H_{4,6/10\&7/9}$), 1.70-1.59 (m, 2H, $H_{6/10}$ & O-H), 1.50 – 1.40 (m, 2H, $H_{7/9}$), 1.33 – 1.19 (m, 1H, $H_{6/10}$), 1.00 (s, 9H, H_{13})

^{13}C NMR (125 MHz, Chloroform-d) δ 182.97 (C_2), 173.21 (C_{11}), 81.59 (C_1), 69.39 (C_8), 56.85 (C_3), 46.62 (C_5), 38.37 (C_{12}), 35.77 (C_4), 31.57 ($C_{7/9}$), 31.45 (C_{14}), 31.36 ($C_{7/9}$), 31.32 ($C_{6/10}$), 30.78 ($C_{6/10}$), 26.00 (C_{13})

HRMS-ESI (*m/z*) [M+Na$^+$] calc. for $C_{16}H_{26}N_2O_3Na$, 317.1836; found 317.1828

IR vmax – 3405 (O-H), 2951, 2926, 2859 (C–H), 1685 (C=O)

R_f - 0.32 in 10% MeOH in dichloromethane

mp 197-198 °C

[α] $^{26}_{D}$ = -4 ° (*c* = 1 in $CHCl_3$)

X-Ray data - shows *trans* OH group at C8 to carbonyl at C11.

Table 4 Crystal data and structure refinement for 13b.

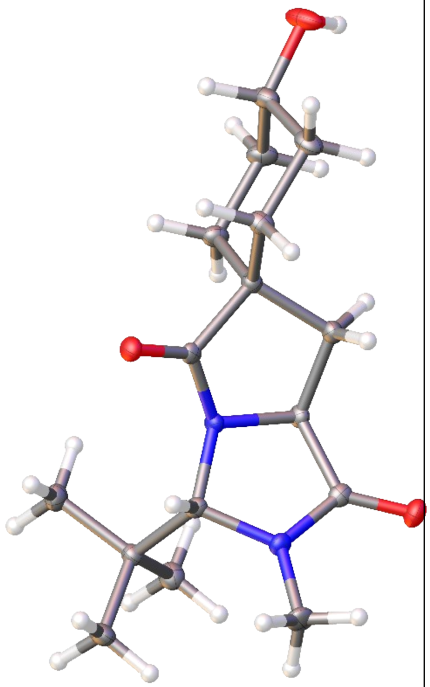

CCDC deposition number	2070023
Empirical formula	$C_{16}H_{26}N_2O_3$
Formula weight	294.39
Temperature/K	99.97
Crystal system	monoclinic
Space group	$P2_1$
a/Å	5.94680(10)
b/Å	13.2423(3)
c/Å	10.0750(3)
α/°	90
β/°	97.9895(15)
γ/°	90
Volume/Å^3	785.70(3)
Z	2
ρ_{calc}g/cm^3	1.244
μ/mm^{-1}	0.086
F(000)	320.0
Crystal size/mm^3	0.424 × 0.266 × 0.074
Radiation	MoKα (λ = 0.71073)
2Θ range for data collection/°	4.082 to 55.932
Index ranges	-7 ≤ h ≤ 7, -17 ≤ k ≤ 17, -13 ≤ l ≤ 13
Reflections collected	18866
Independent reflections	3773 [R_{int} = 0.0301, R_{sigma} = 0.0227]
Data/restraints/parameters	3773/1/198
Goodness-of-fit on F^2	1.051
Final R indexes [I>=2σ (I)]	R_1 = 0.0318, wR_2 = 0.0801
Final R indexes [all data]	R_1 = 0.0339, wR_2 = 0.0812
Largest diff. peak/hole / e Å^{-3}	0.25/-0.17

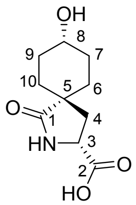

(3R,5s,8S)-8-hydroxy-1-oxo-2-azaspiro[4.5]decane-3-carboxylic acid (14)

To a solution of **13a** (30 mg, 0.10 mmol) in water (0.5 mL), sodium hydroxide (12 mg, 0.30 mmol) in a sealed tube. The mixture was stirred at 90 °C for 16 hours. The resulting mixture was then neutralised (approx. pH = 8) and lyophilized. The mixture was redissolved in ethanol and filtered to remove excess salt. The product was purified by Hypercarb HPLC with a gradient of 0-100% acetonitrile in 100 mM ammonium bicarbonate buffer to yield **14** (19.3 mg, 80%) as a white powder.

^{1}H NMR (500 MHz, Deuterium Oxide) δ 4.16 (t, J = 7.5 Hz, 1H, H_3), 3.99 (s, 1H, H_8), 2.50 (t, J = 11.1 Hz, 1H, H_4), 2.01 (d, J = 7.2 Hz, 1H, H_4), 1.96 – 1.81 (m, 2H, $H_{6\&10}$), 1.81 – 1.59 (m, 4H, $H_{7\&9}$), 1.43 – 1.24 (m, 2H, $H_{6\&10}$).

^{13}C NMR (125 MHz, Deuterium Oxide) δ 184.73 (C_1), 179.73 (C_2), 65.56 (C_8), 54.62 (C_3), 44.32 (C_5), 36.01 (C_4), 27.77 ($C_{7/9}$), 27.71 ($C_{7/9}$), 27.23 ($C_{6/10}$), 26.56 ($C_{6/10}$).

HRMS-Nanospray (*m/z*) $[M-H]^-$ calc. for $C_{10}H_{14}NO_4$, 212.0923; found 212.0928.

CD spectra - showed end absorption only.

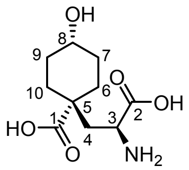

(1s,4*R*)-1-((*S*)-2-amino-2-carboxyethyl)-4-hydroxycyclohexane-1-carboxylic acid (15)

To a solution of **13a** (236 mg, 0.8 mmol) in water (2 mL), barium hydroxide (1.645 g, 9.6 mmol) and barium carbonate (158 mg, 0.8 mmol) was added. The mixture was stirred at 80 °C for 20 hours. The mixture was diluted with H_2O (5 mL) and dry ice was added to precipitate barium hydroxide as barium carbonate. Any solids were removed by filtration and the solution was concentrated *in vacuo*. Purification was carried out by reversed phase column chromatography on C18 silica gel eluting with H_2O + 0.1% TFA to yield **15** as the TFA salt (207 mg, 75 %) as a colourless solid.

^{1}H NMR (400 MHz, Deuterium Oxide) δ 4.04 (dd, *J* = 7.1, 5.1 Hz, 1H, H_3), 3.61 (tt, *J* = 9.9, 4.2 Hz, 1H, H_8), 2.30 (dd, *J* = 15.1, 7.2 Hz, 1H, H_4), 2.23 – 2.02 (m, 2H, $H_{6\&10}$), 1.87 (dd, *J* = 15.1, 5.2 Hz, 1H, H_4), 1.87 – 1.76 (m, 2H, $H_{7\&9}$), 1.45 – 1.18 (m, 4H, $H_{6,7,9\&10}$).

^{13}C NMR (100 MHz, Deuterium Oxide) δ 179.2 (C_1), 171.7 (C_2), 162.9 (q, *J* = 35.7 Hz, CF_3COO^-), 116.2 (q, *J* = 292.0 Hz, $\mathit{C}F_3COO^-$), 117.7 (C_1), 114.8 (C_1), 111.9 (C_1), 68.8 (C_8), 49.5 (C_3), 44.4 (C_5), 39.5 (C_4), 32.0 ($C_{6/10}$), 30.5 ($C_{7\&9 \text{ and } 6/10}$).

HRMS-Nanospray (*m/z*) $[M+H]^+$ calc. for $C_{10}H_{18}NO_5$, 232.1185; found 232.1176

IR νmax – 2941(C-H), 1665 (C=O)

[α] $^{22}_{D}$ = 8 ° (*c* = 1 in H_2O)

3. Additional data

3.1 Hydrolysis optimization

Table 2 - Exploring various bases for the hydrolysis of 8a.

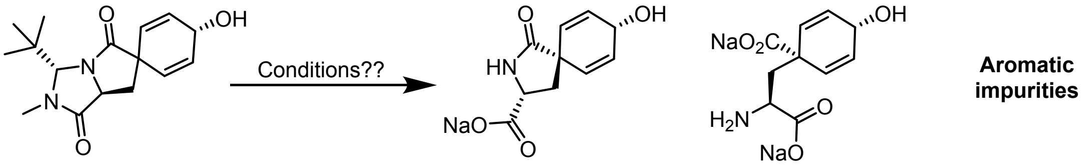

SM **8a** — **A** **9** — **B** **1** — **C**

	Base (eq.)	Eq.	Solvent	Conc. (M)	Ratio SM	Ratio A	Ratio B	Ratio C	Comment
**a**	$Ba(OH)_2$	3	D_2O	0.2	52	39	-	9	Base added last
**b**	BaO	3	H_2O	0.2	63	31	-	6	Sonicated & Concentrated
**c**	NaOH	3	H_2O	0.2	27	40	-	33	Sonicated & Concentrated
**d**	LiOH	3	D_2O	0.2	-	-	-	-	Sonicated. Complex product mixture
**e**	KOH	3	D_2O	0.2	50	23	-	27	Sonicated
**f**	$Ca(OH)_2$	3	D_2O	0.2	94	-	-	6	Sonicated
**g**	$NaOH/NaCO_3$	3	D_2O	0.2	42	22	-	36	Sonicated

All reactions performed at 30 ^{o}C , overnight.

Table 3 - Exploring various solvents, equivalents & concentrations for the barium hydroxide hydrolysis of 8a.

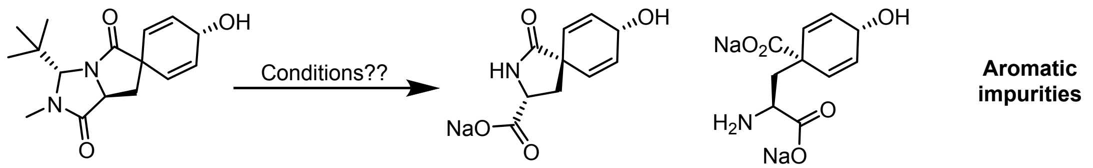

SM 8a; A 9; B 1; C

	Base (eq.)	Eq.	Solvent	Conc. (M)	Ratio				Comment
					SM	A	B	C	
**a**	$Ba(OH)_2$	3	D_2O	0.2	52	39	-	9	Base added last.
**b**	$Ba(OH)_2$	3	D_2O:dioxane	0.2	99	t	-	t	Base added last.
**c**	$Ba(OH)_2$	3	D_2O:MeOD	0.2	83	14	-	3	Base added last.
**d**	$Ba(OH)_2$	3	D_2O	0.4	62	32	-	6	Base added last.
**e**	$Ba(OH)_2$	3	H_2O	0.1	40	47	-	13	Sonicated & Concentrated.
**f**	$Ba(OH)_2$	12	H_2O	0.1	22	57	t	21	Sonicated & Concentrated.
**g**	$Ba(OH)_2$	3	H_2O	0.05	61	32	-	7	Sonicated & Concentrated.
**h**	$Ba(OH)_2$	12	D_2O	0.05	44	40	t	16	Sonicated & Concentrated.

All reactions performed at 30 ^{o}C, overnight. t – trace (<1%).

3.2 Spirocyclisation optimization

Table 1 - Conditions for transferring the spirocyclisation reaction to the microwave.

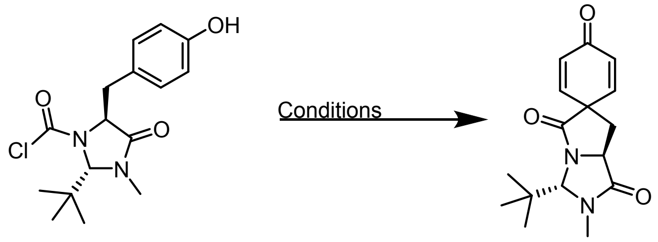

6 7

	Solvent	Time	Temp (^oC)	Base	KI (eq.)	Yield (%)	Comments
**a**	DCM:toluene (4:1)[a]	72 h	60	TEA	1.1	82	Conventional heating
**b**	Acetonitrile[b]	5 min	150	Lutidine	1.1	NMR - full conversion	Microwave test
**c**	Acetonitrile[b]	5 min	150	TEA	1.1	NMR - full conversion	Microwave test
**d**	Acetonitrile[b]	5 min	150	TEA	0	NMR - full conversion	Microwave test

[a] 0.5M [b] 0.15M

4. NMR Spectra

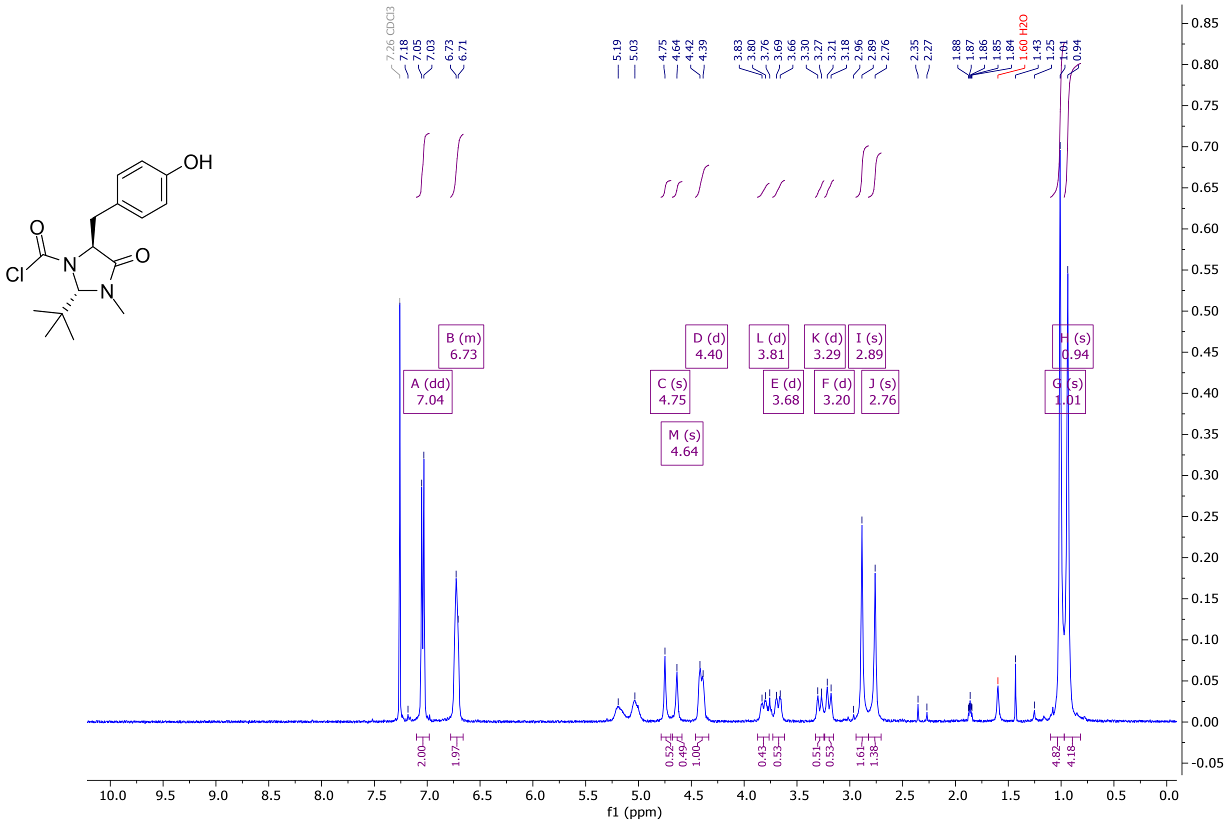

^{1}H NMR of 6

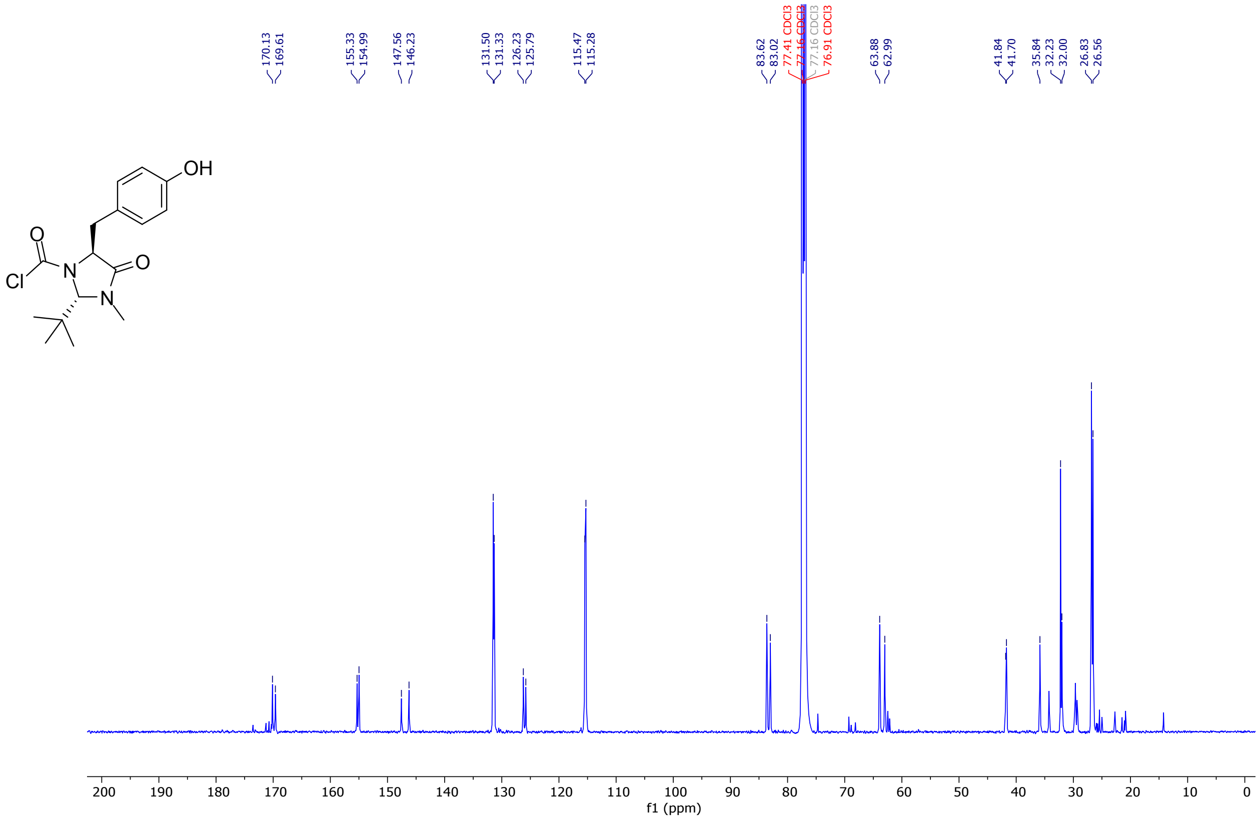

^{13}C NMR of 6

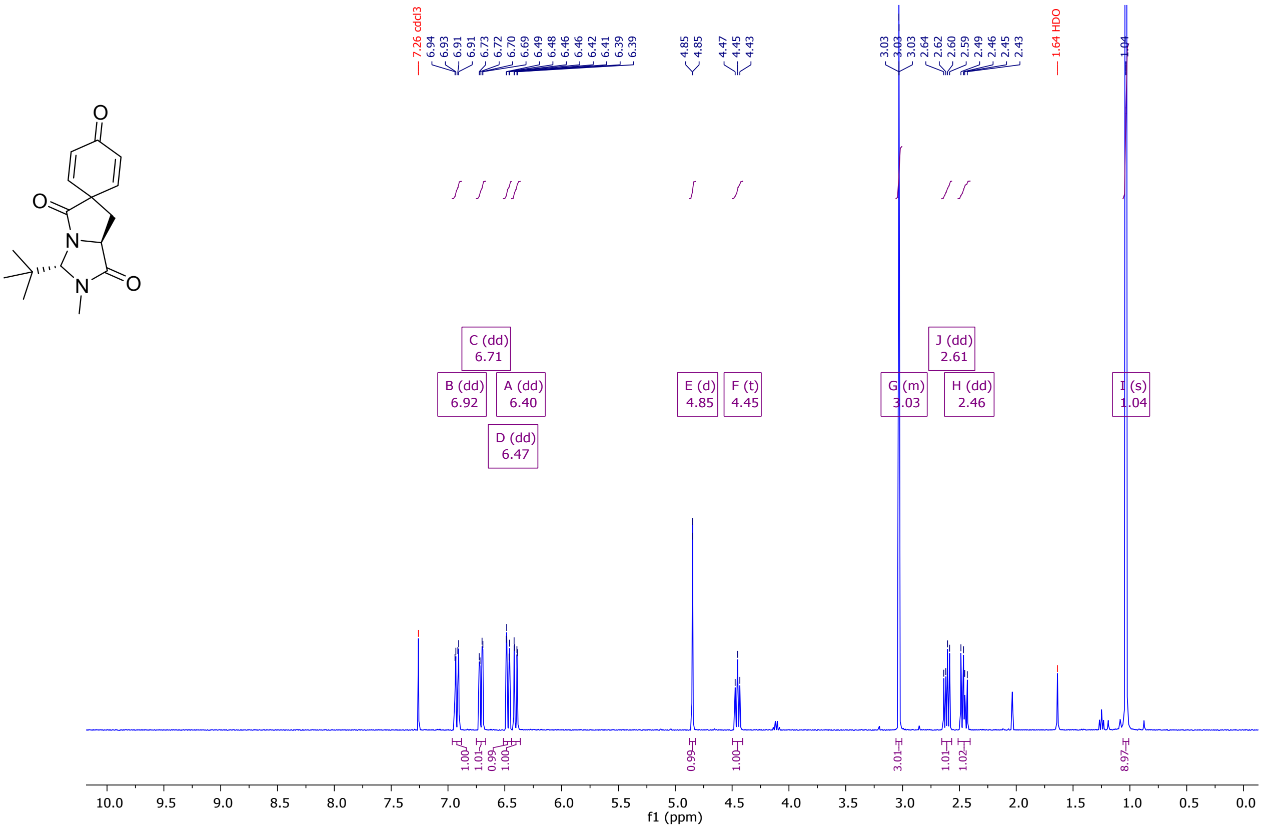

^{1}H NMR of 7

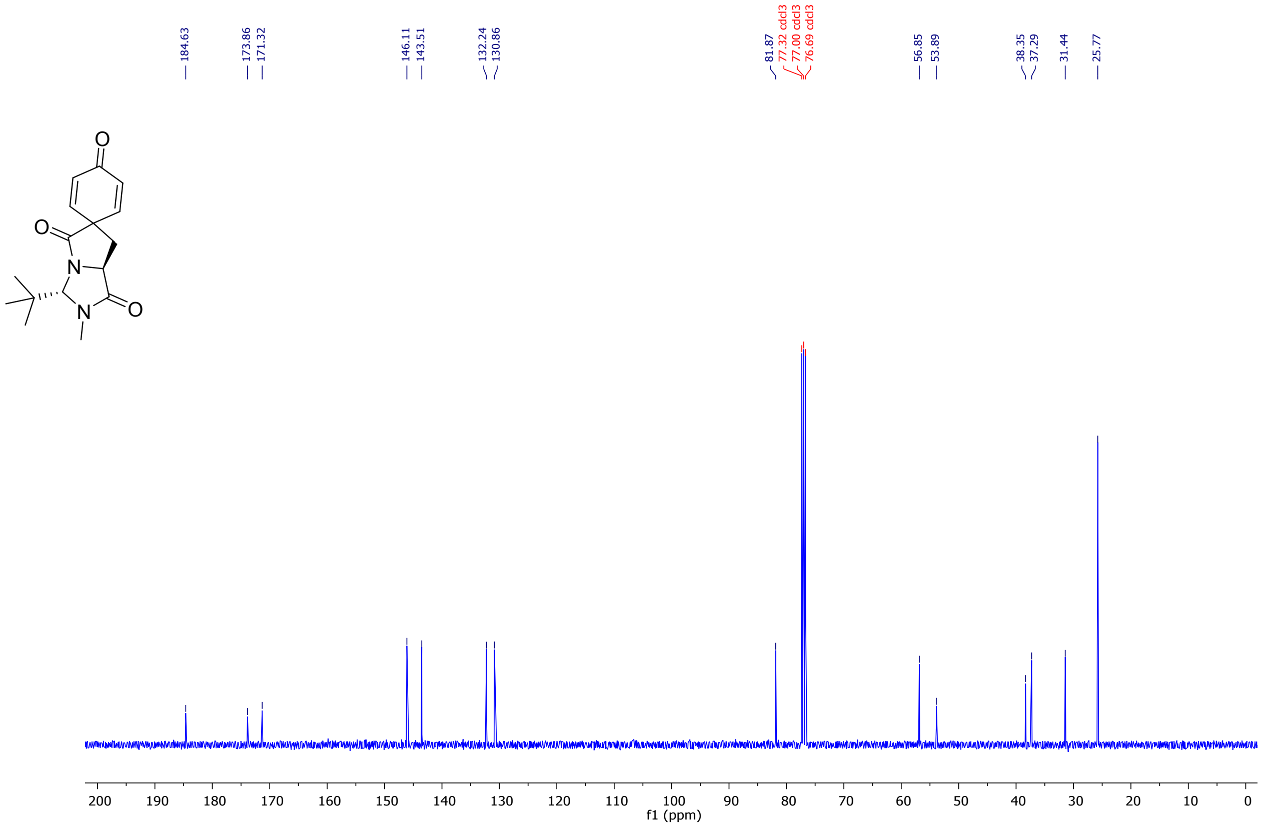

^{13}C NMR of 7

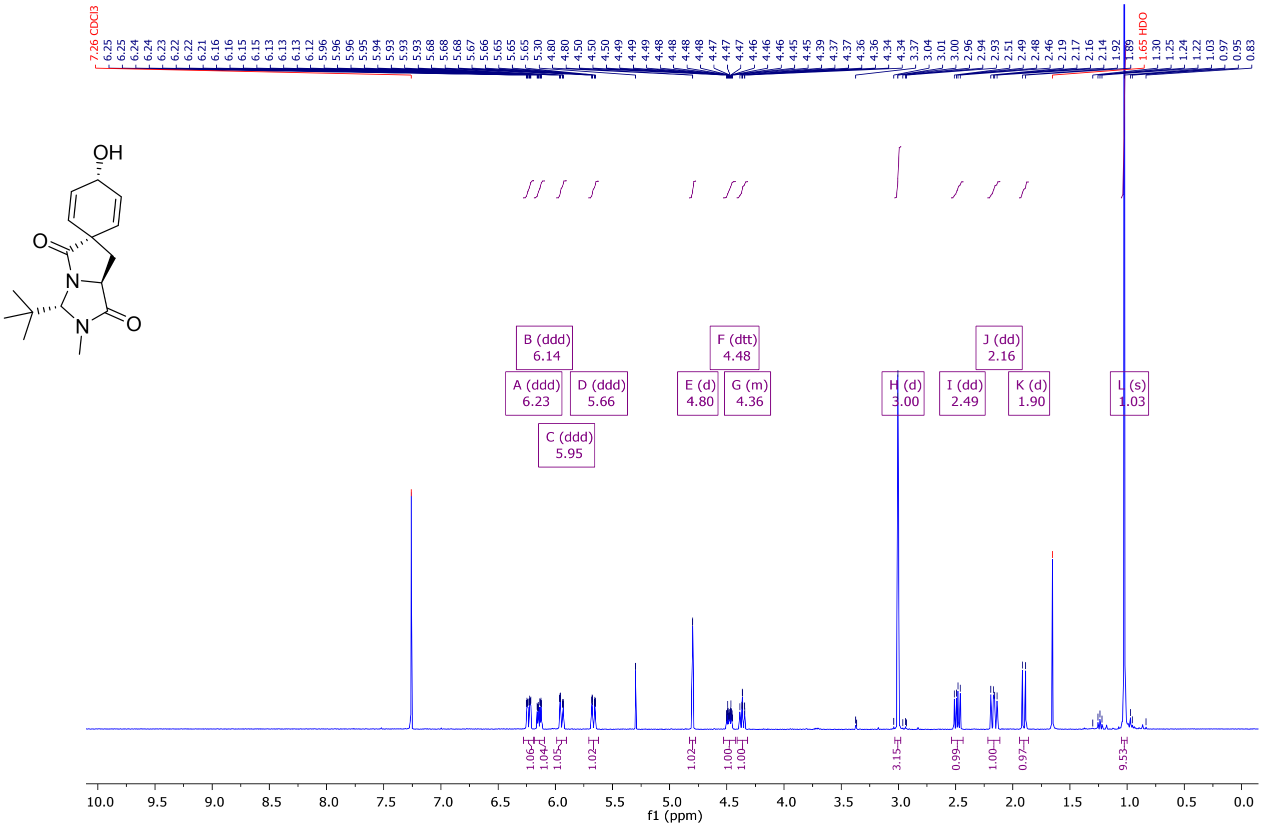

^{1}H NMR of 8a

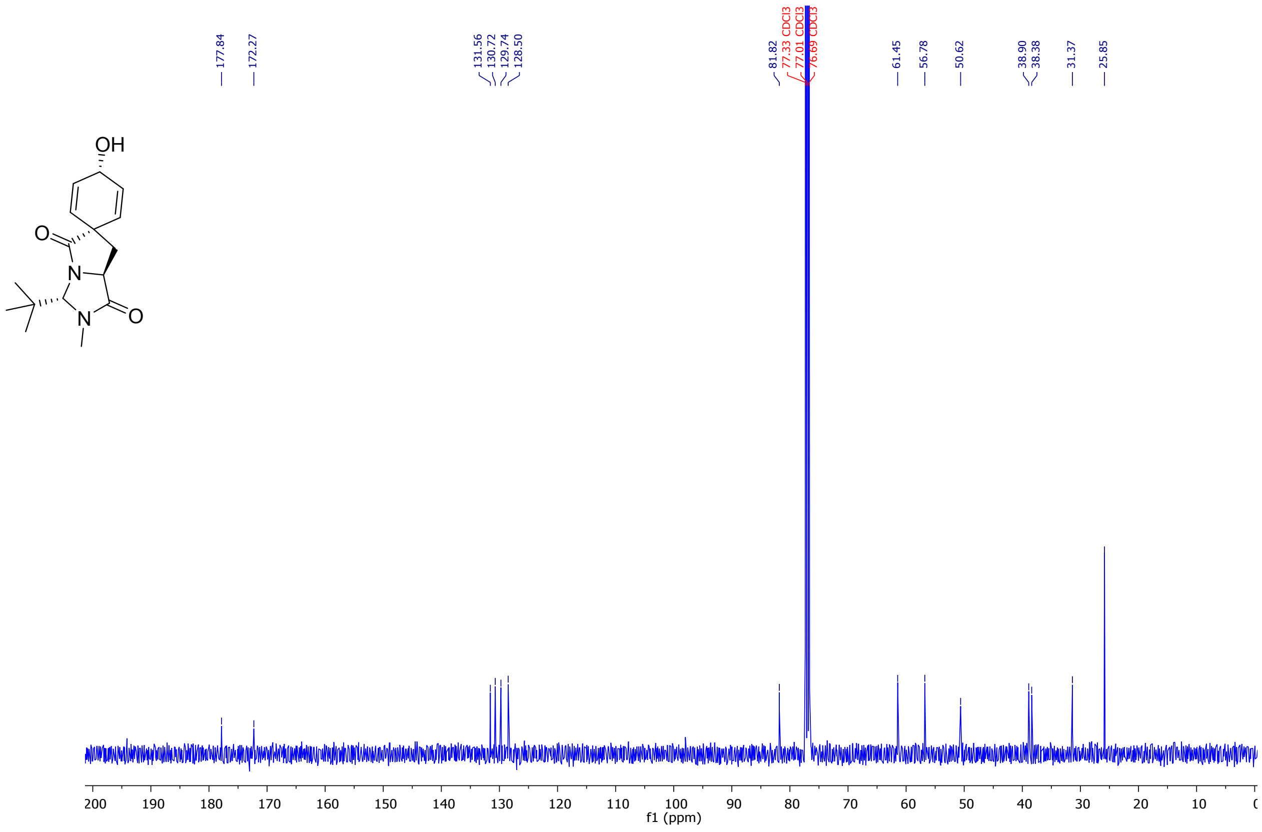

^{13}C NMR of 8a

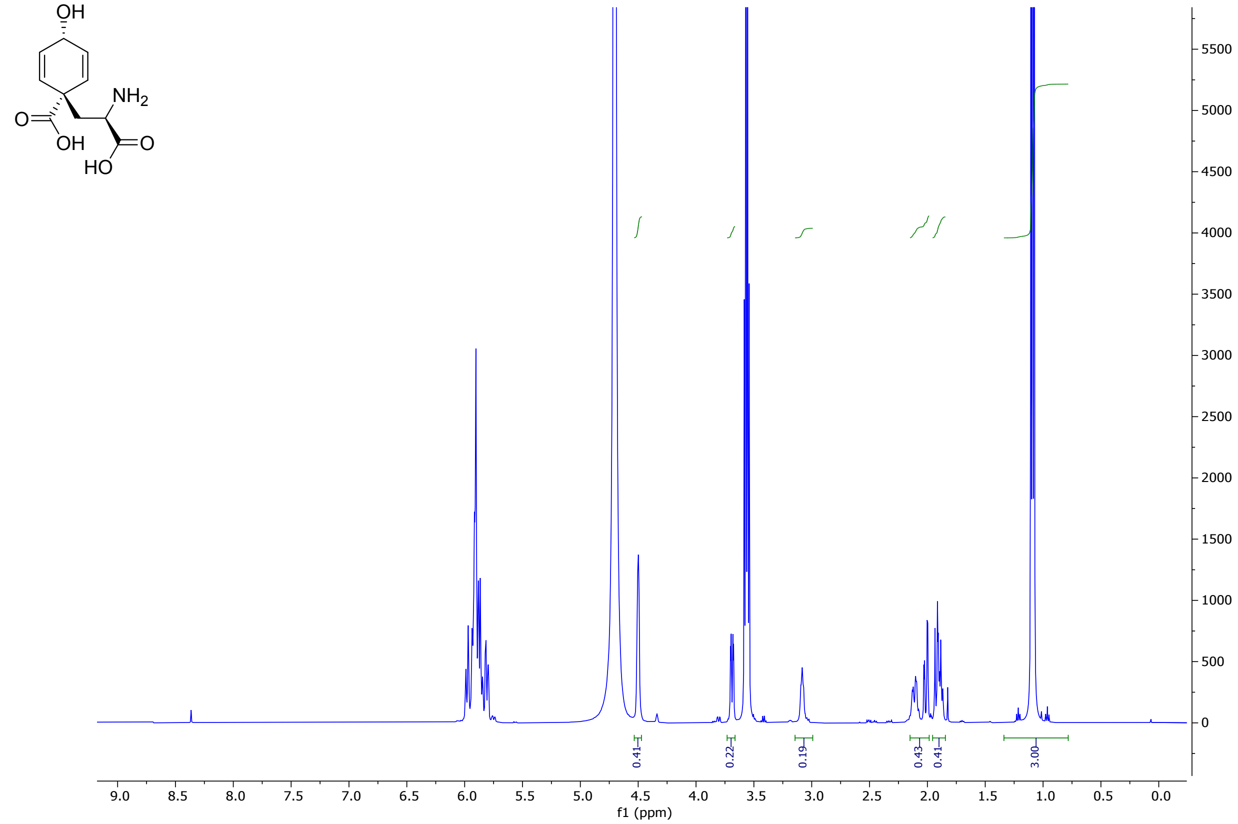

Quantitative ^{1}H NMR of 1 (mixture of salts)

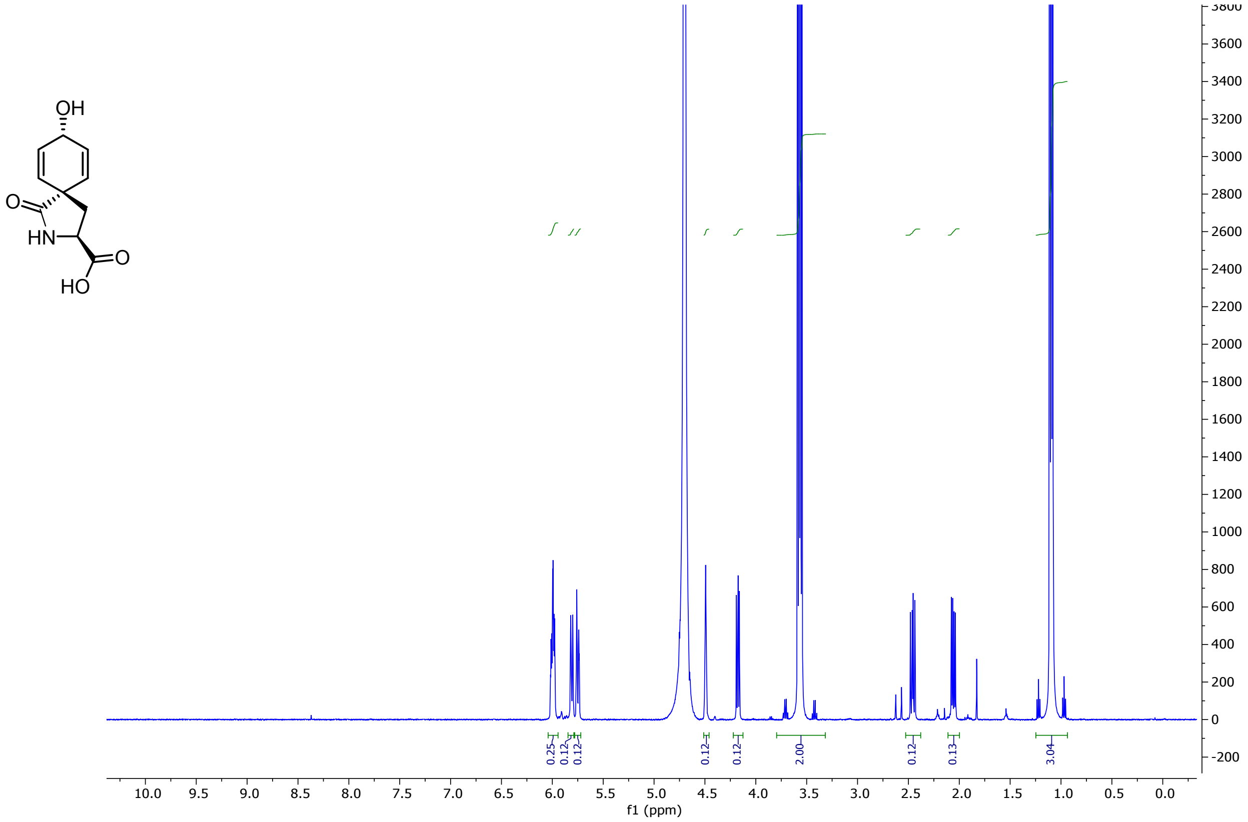

Quantitative ^{1}H NMR of 9

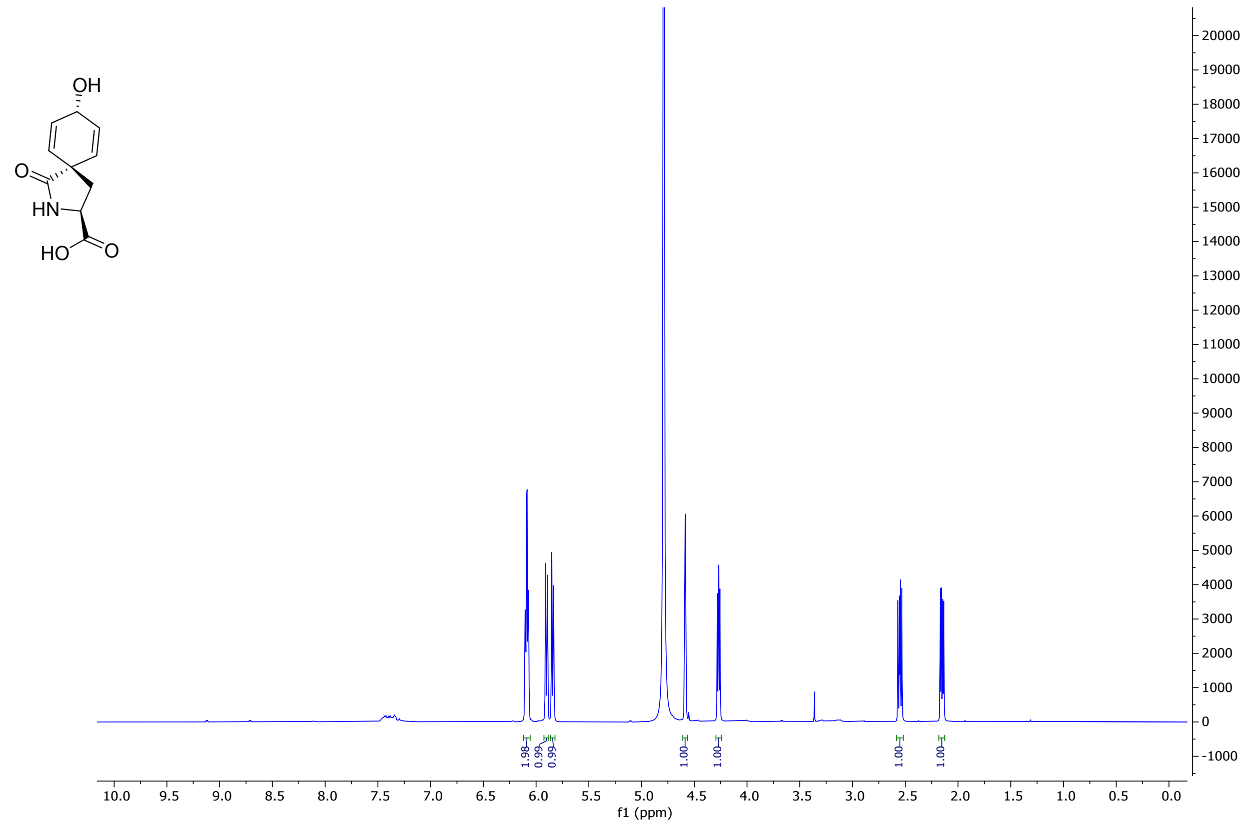

^{1}H NMR of 9

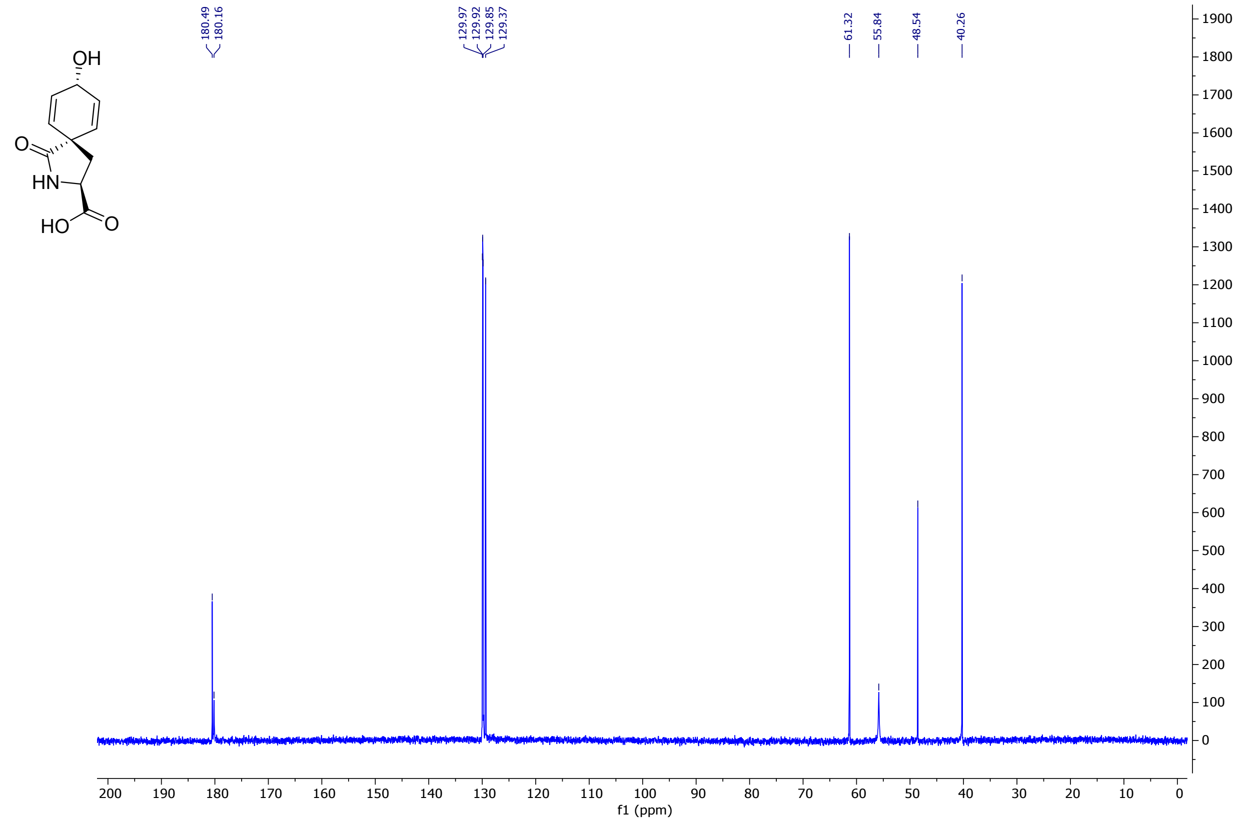

^{13}C NMR of 9

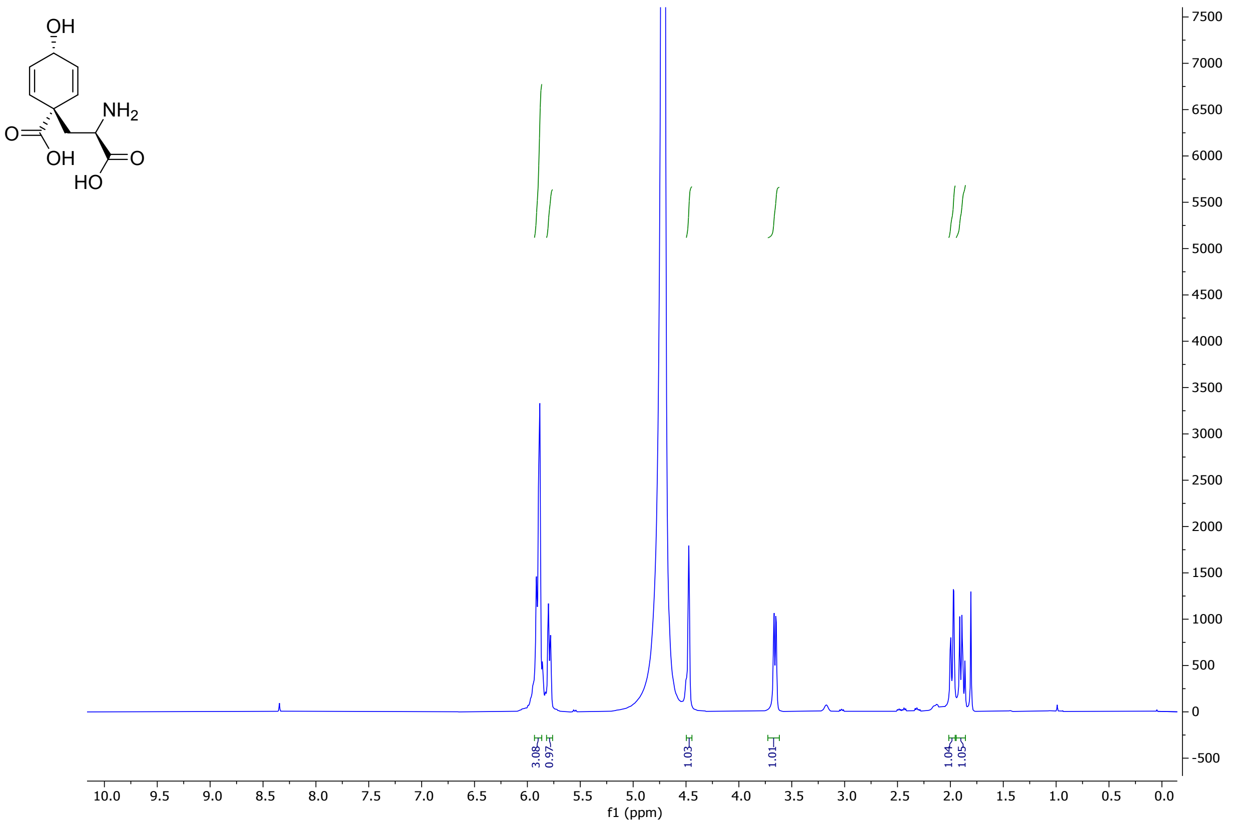

^{1}H NMR of 1

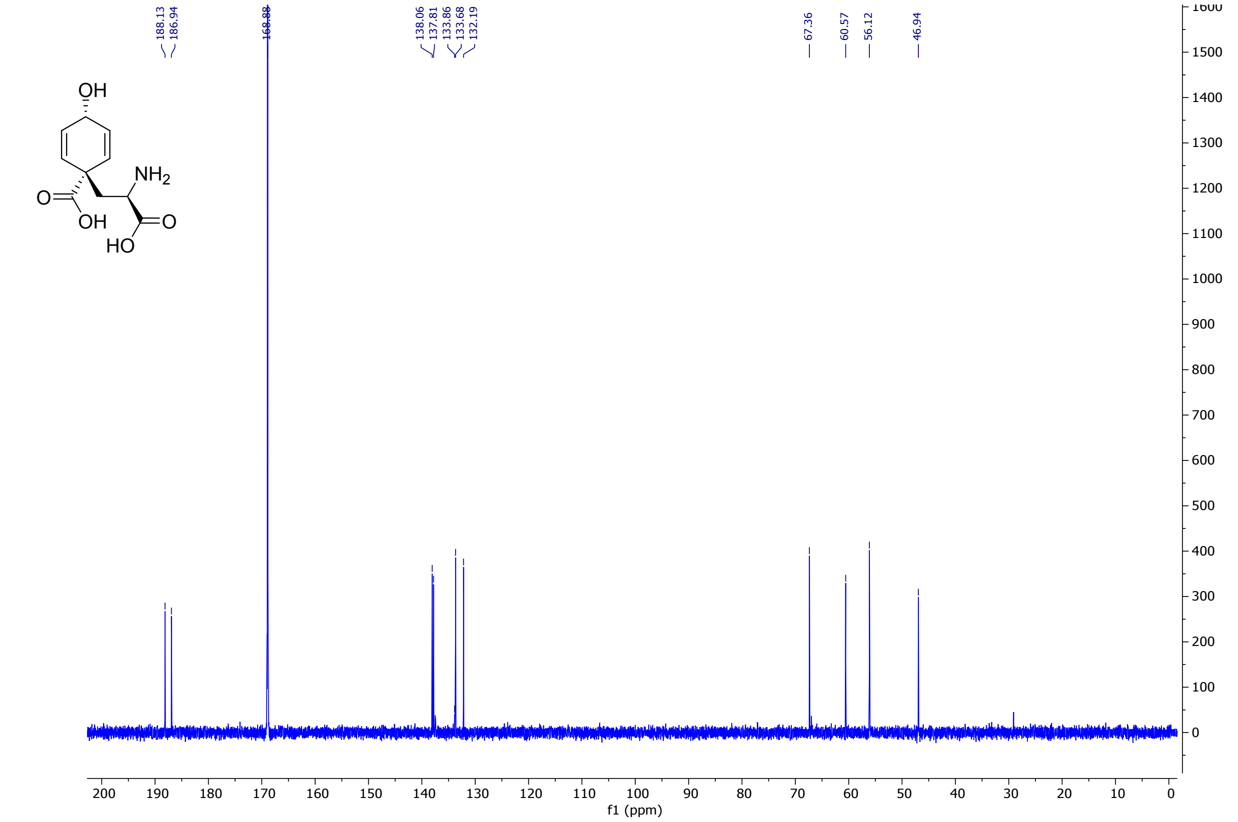

^{13}C NMR of 1 (referenced to sodium carbonate)[4]

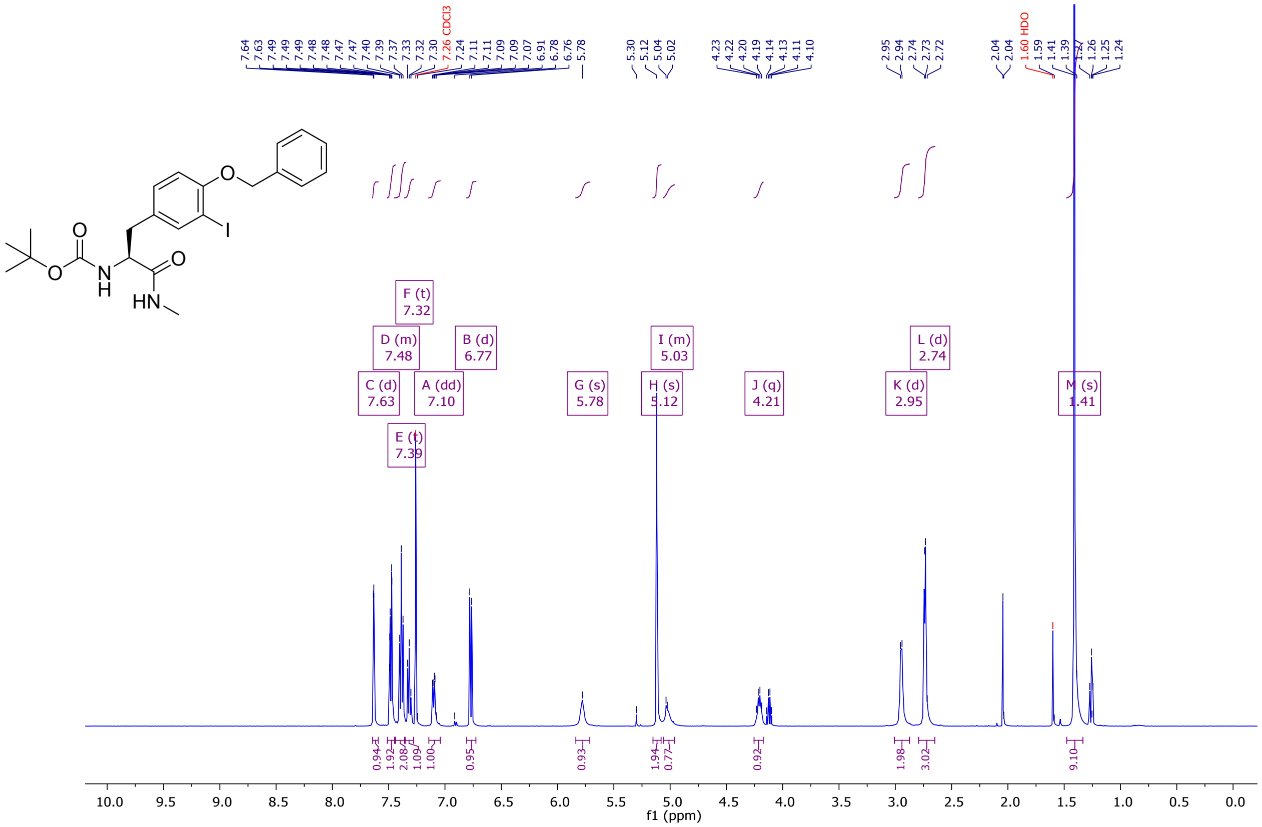

^{1}H NMR of S2

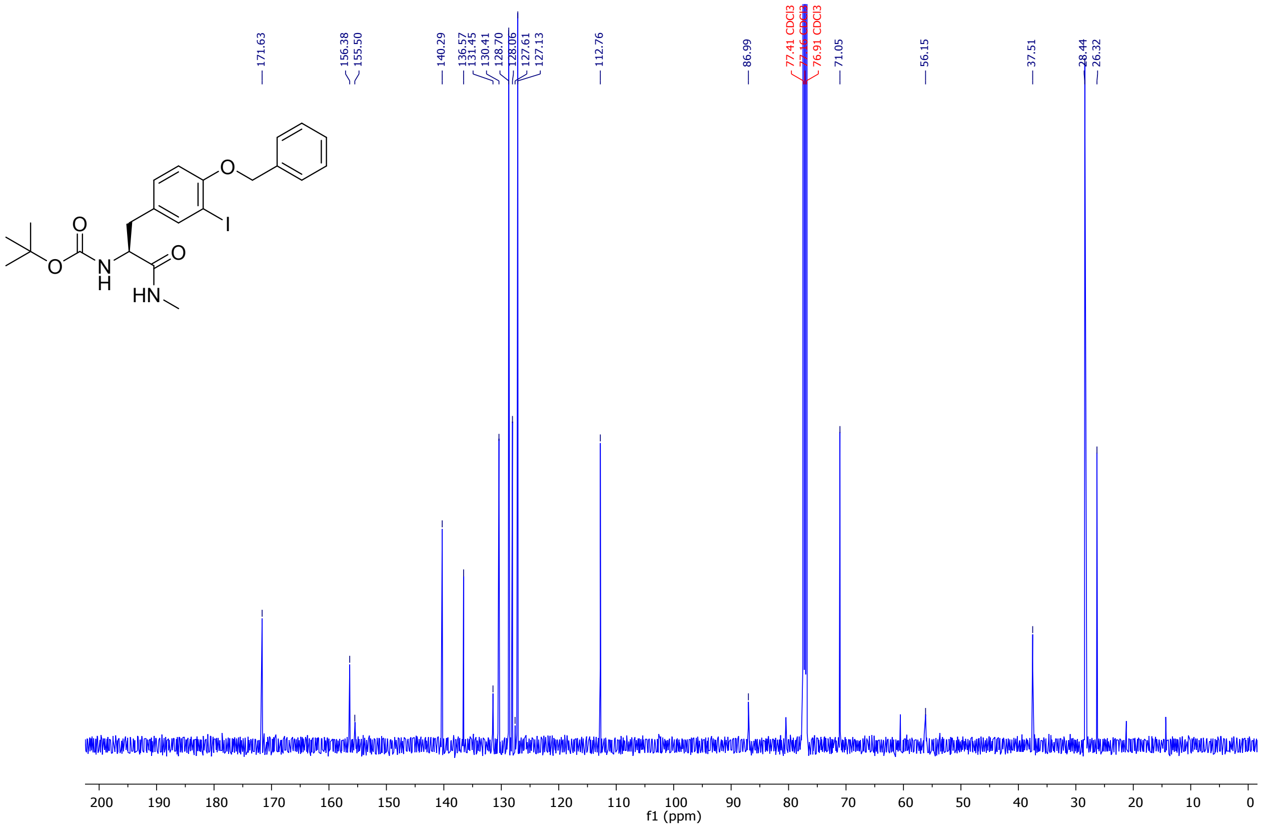

^{13}C NMR of S2

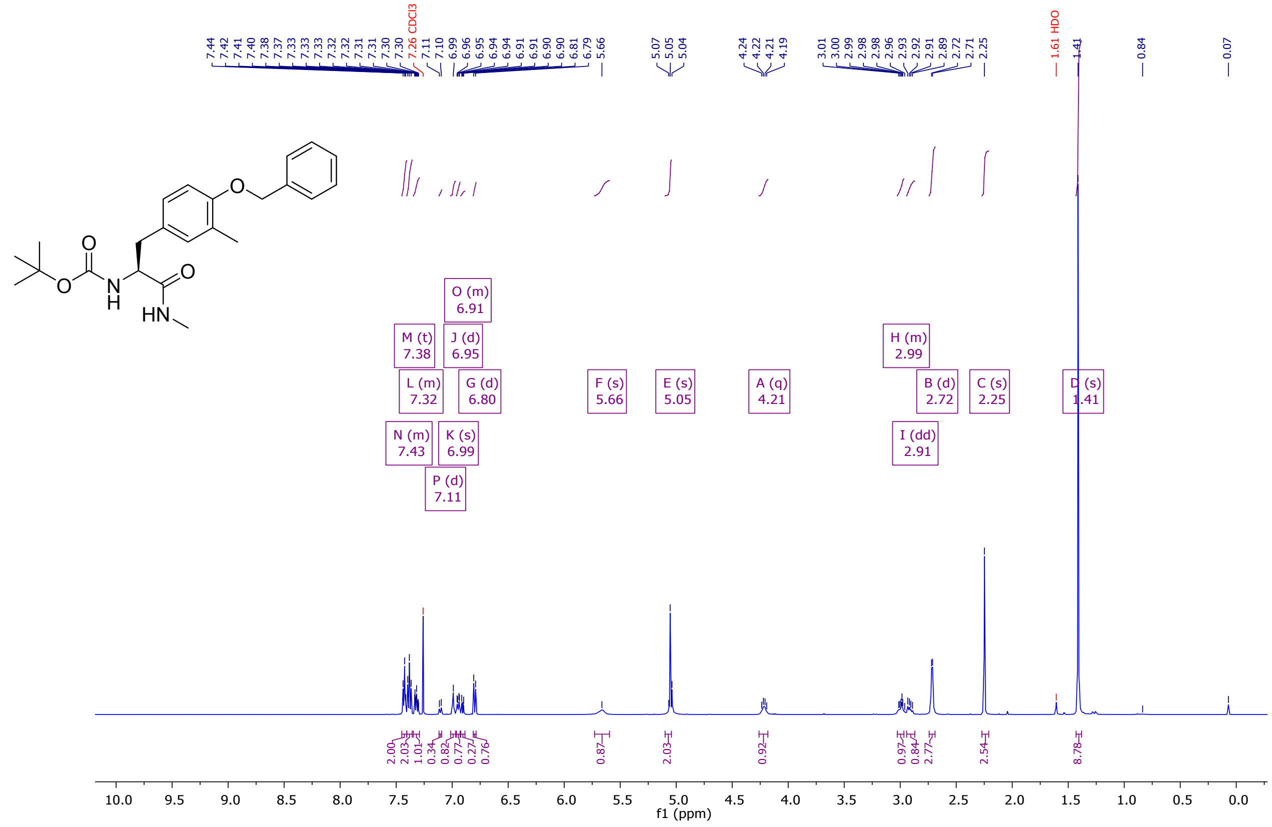

^{1}H NMR of S3

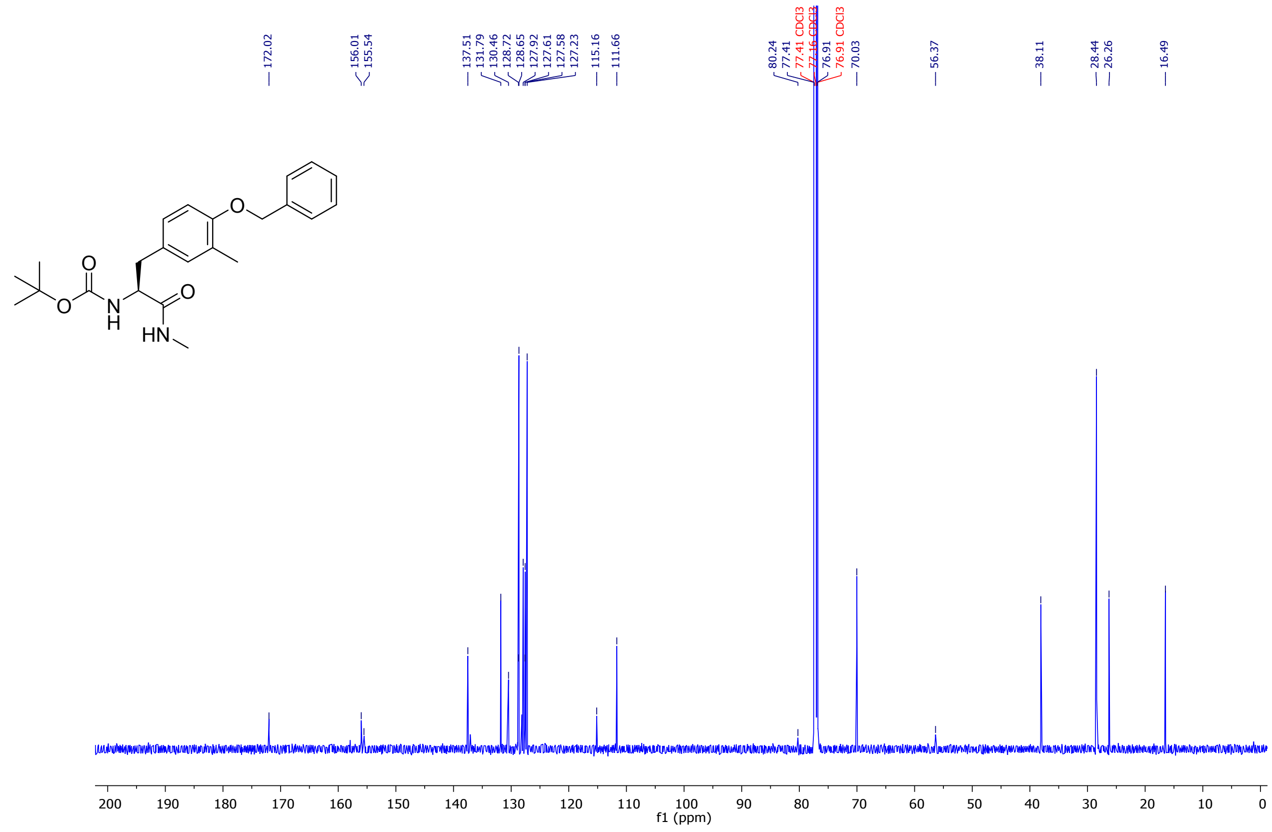

^{13}C NMR of S3

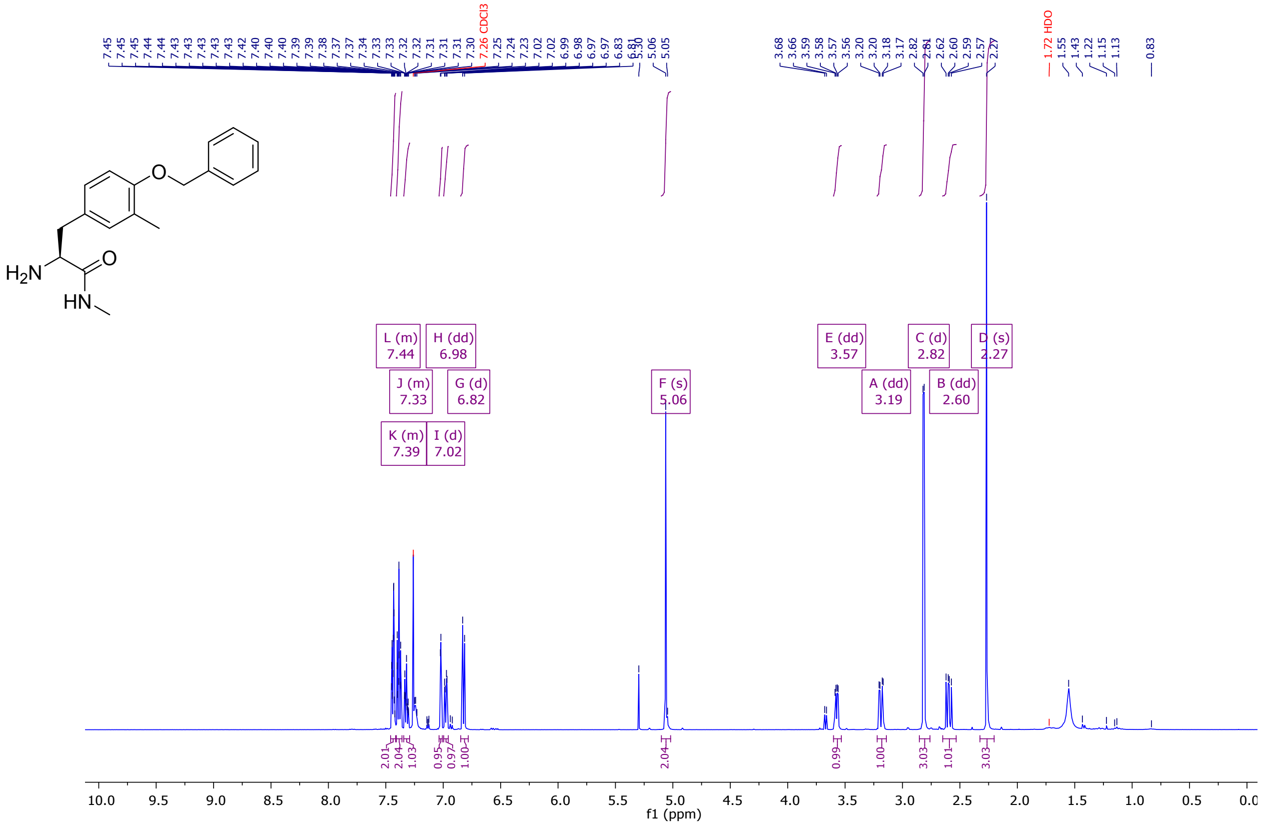

^{1}H NMR of S4

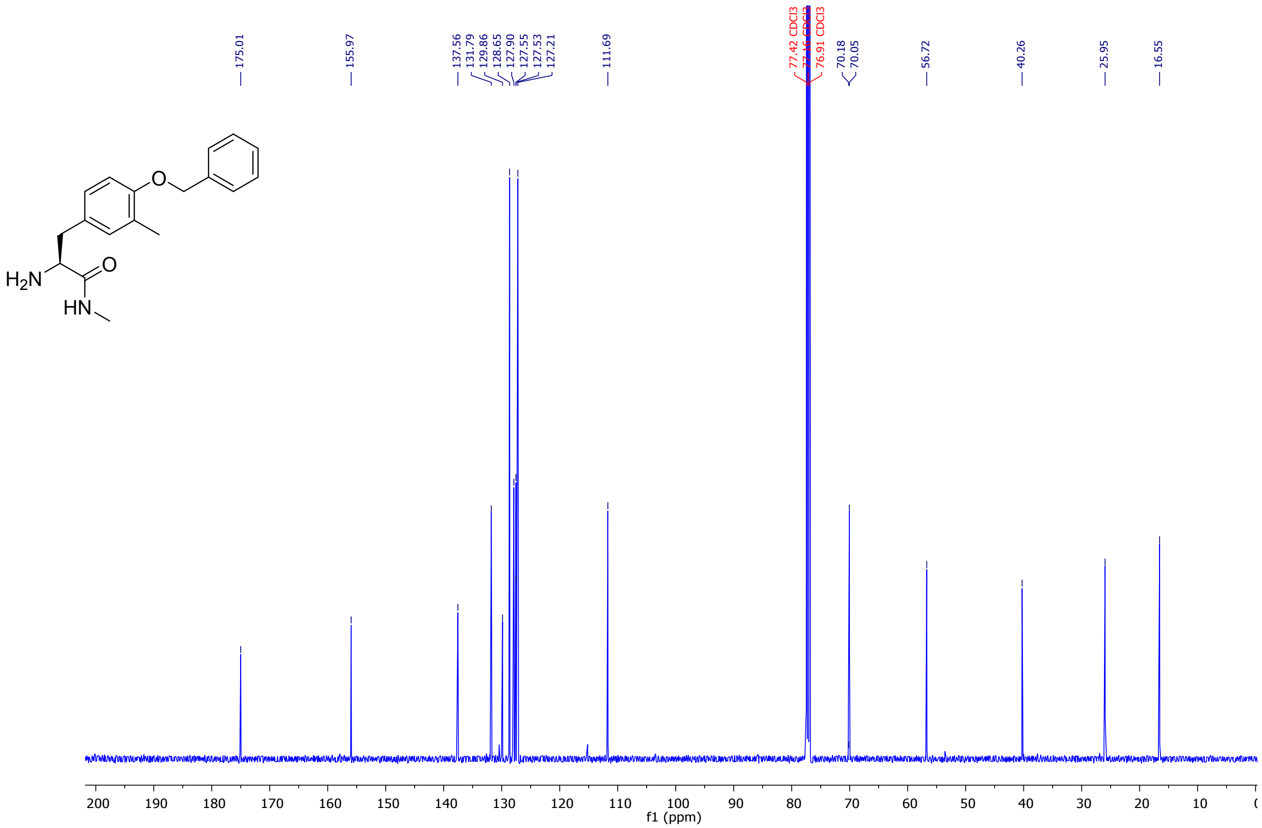

^{13}C NMR of S4

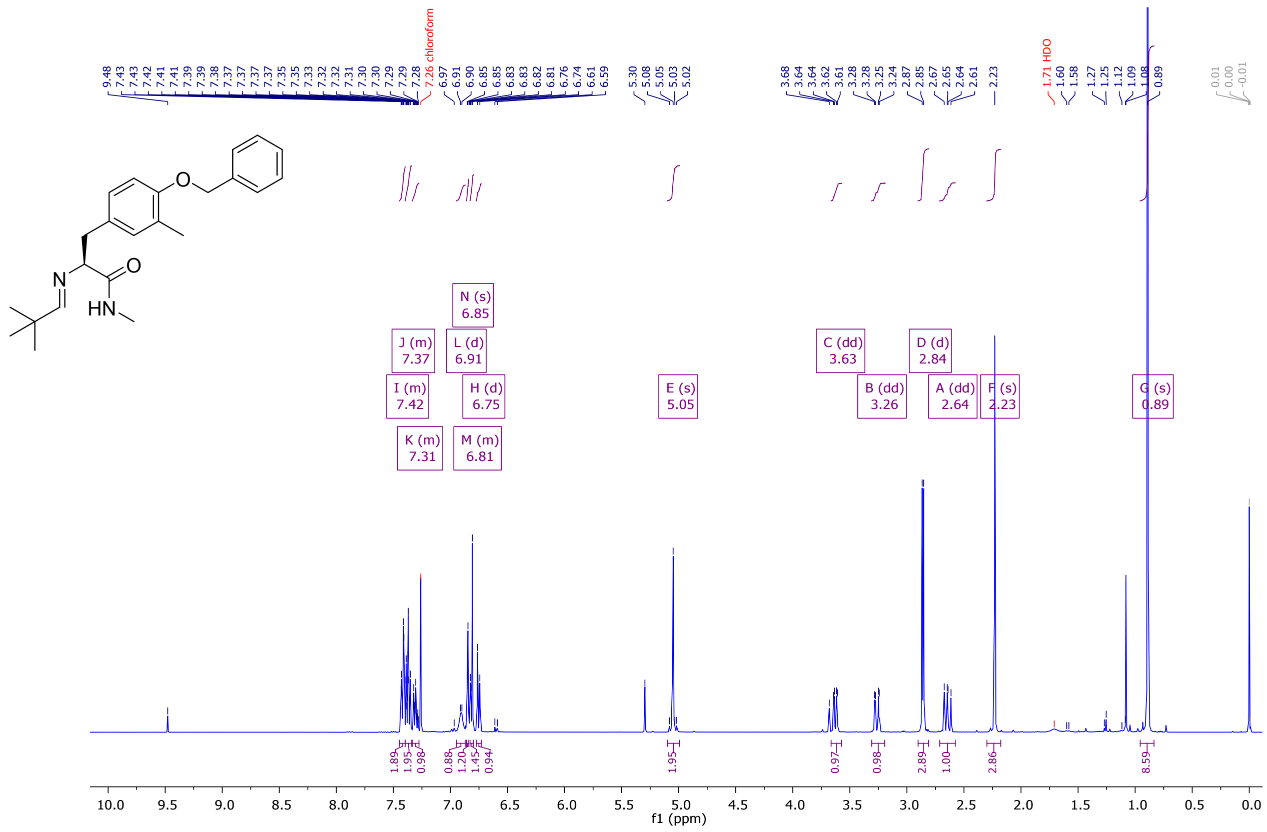

^{1}H NMR of S5

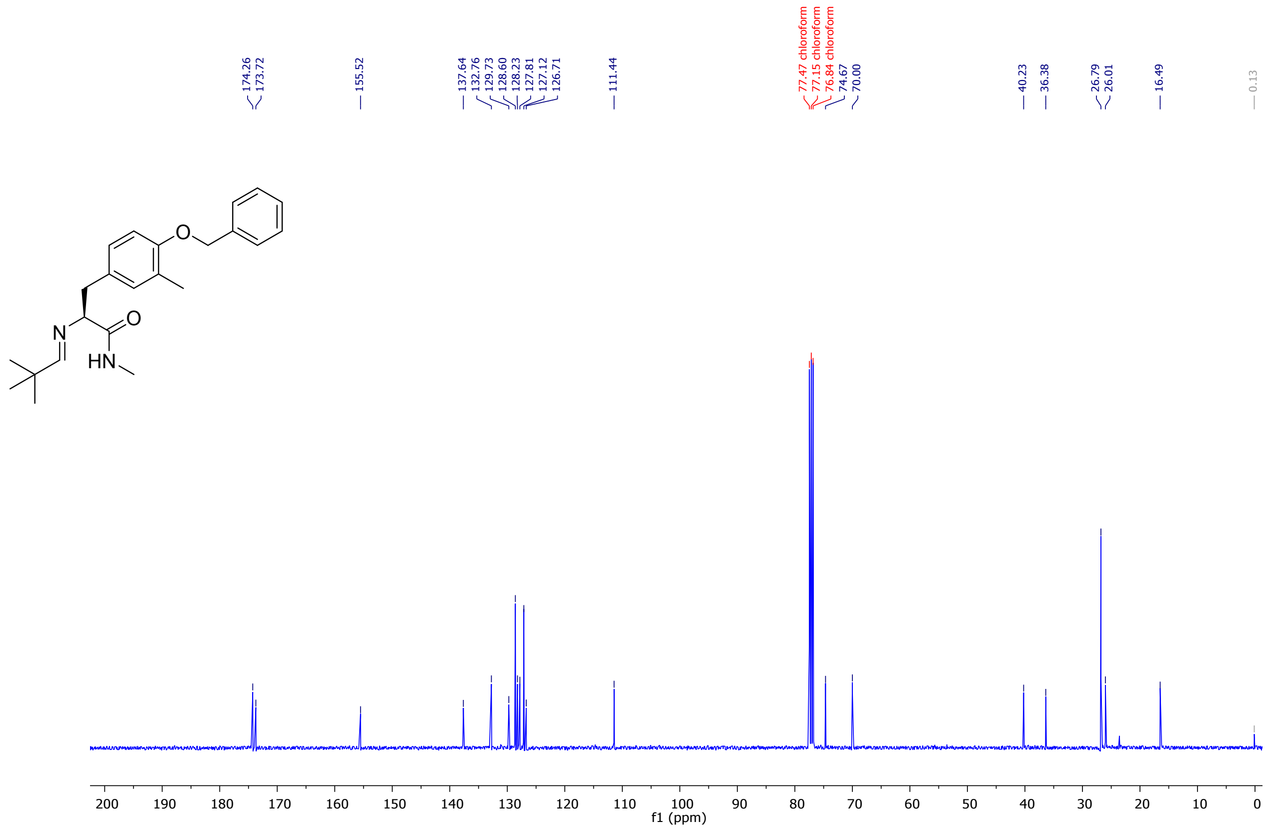

^{13}C NMR of S5

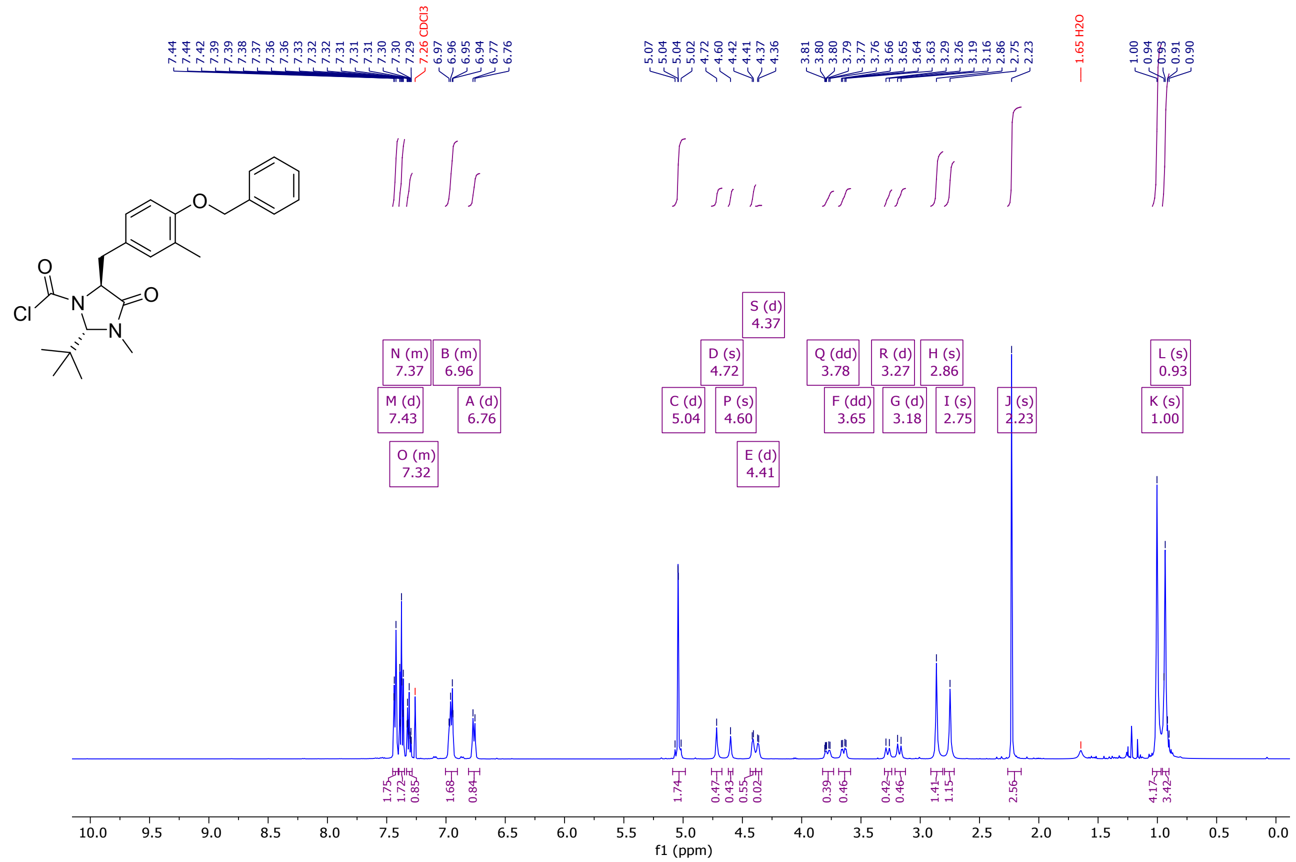

^{1}H NMR of S6

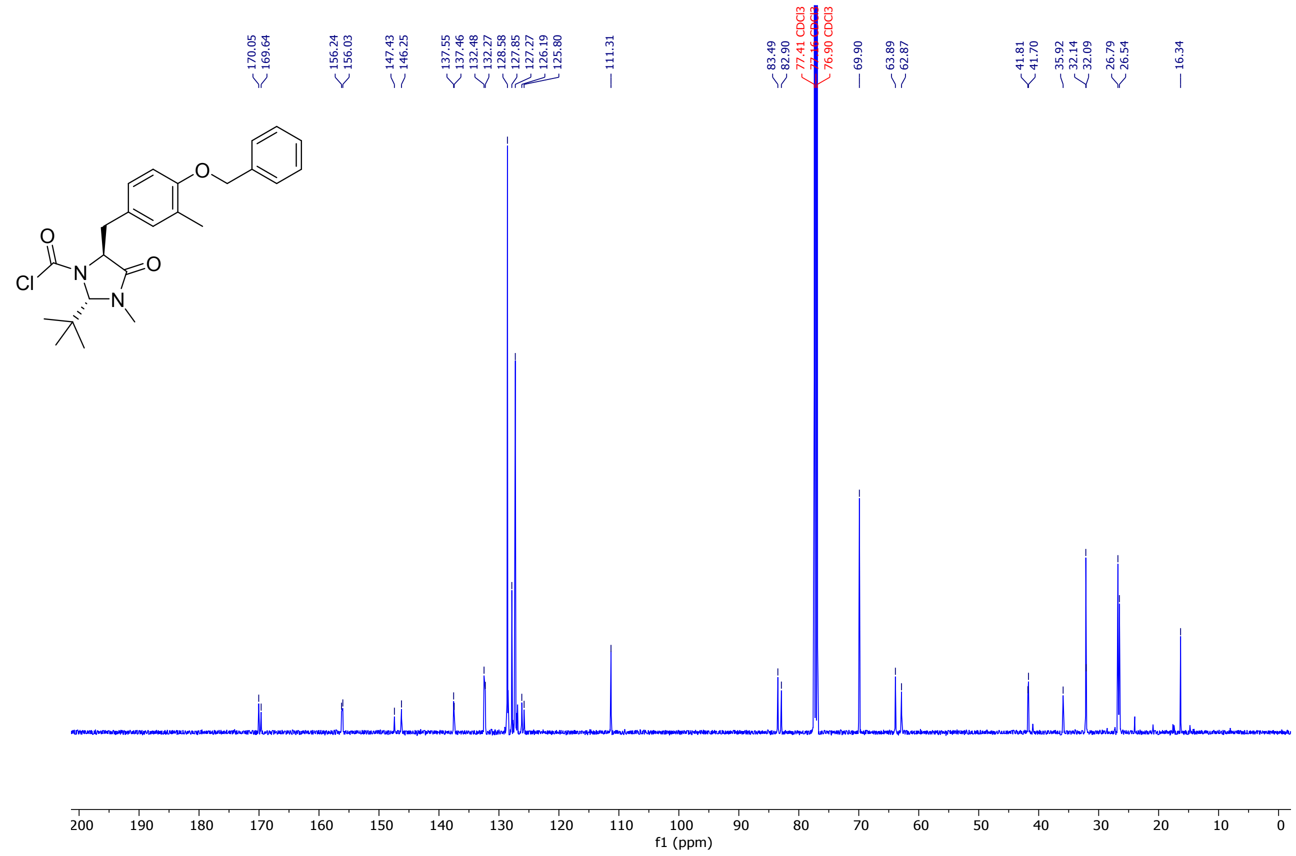

^{13}C NMR of S6

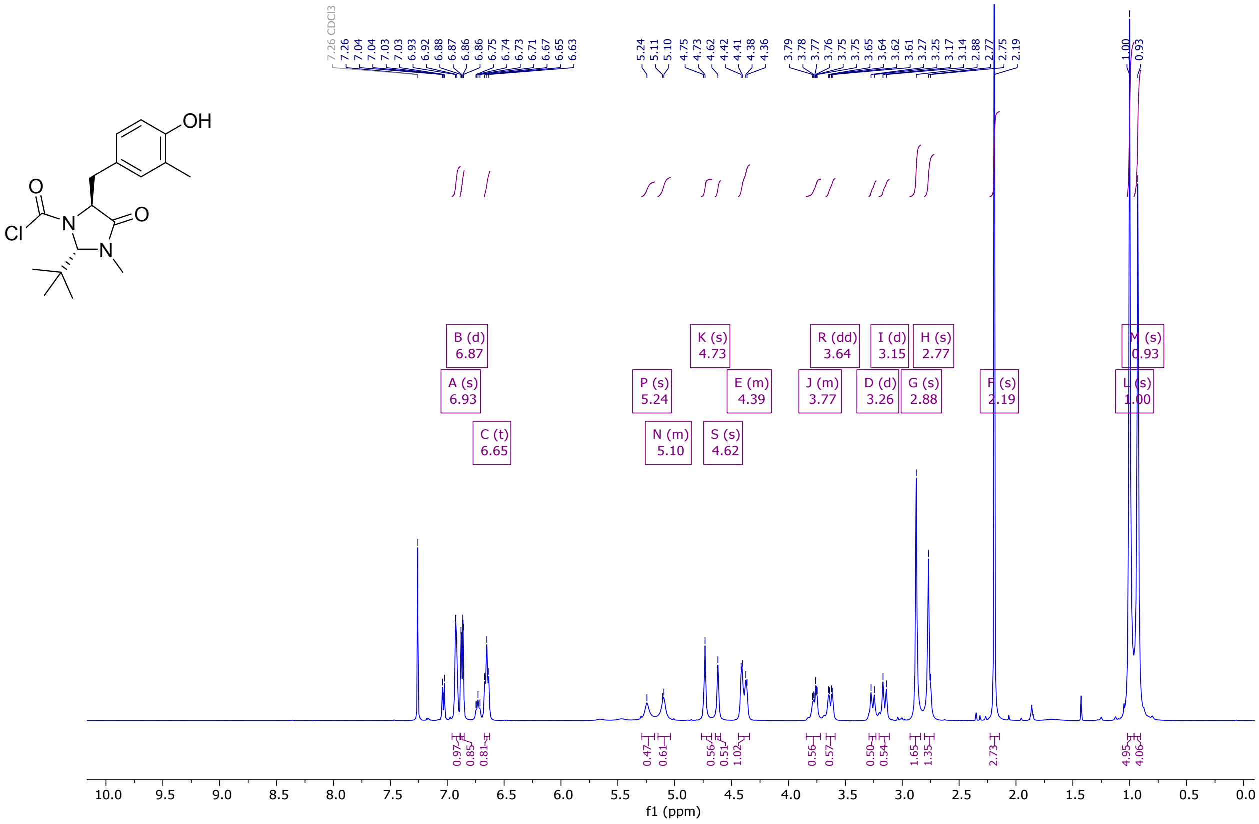

^{1}H NMR of 22

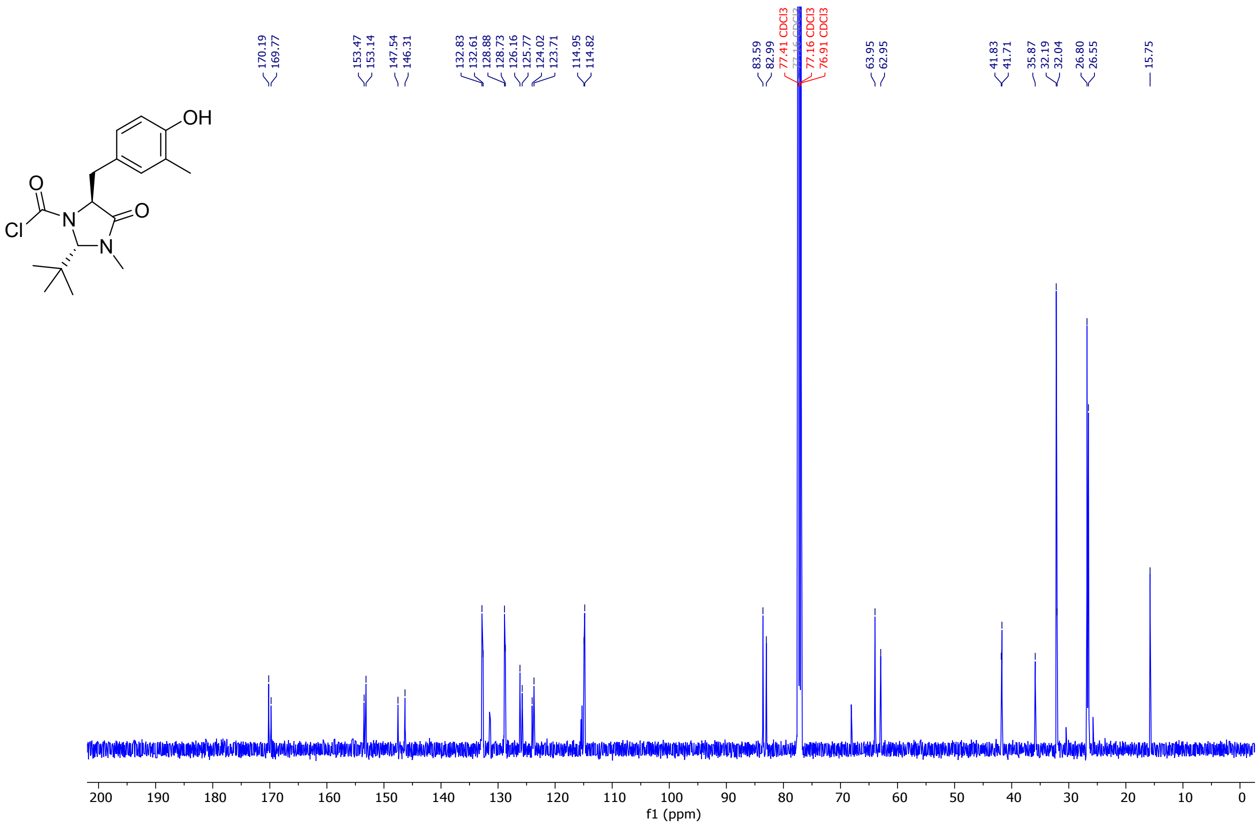

^{13}C NMR of 22

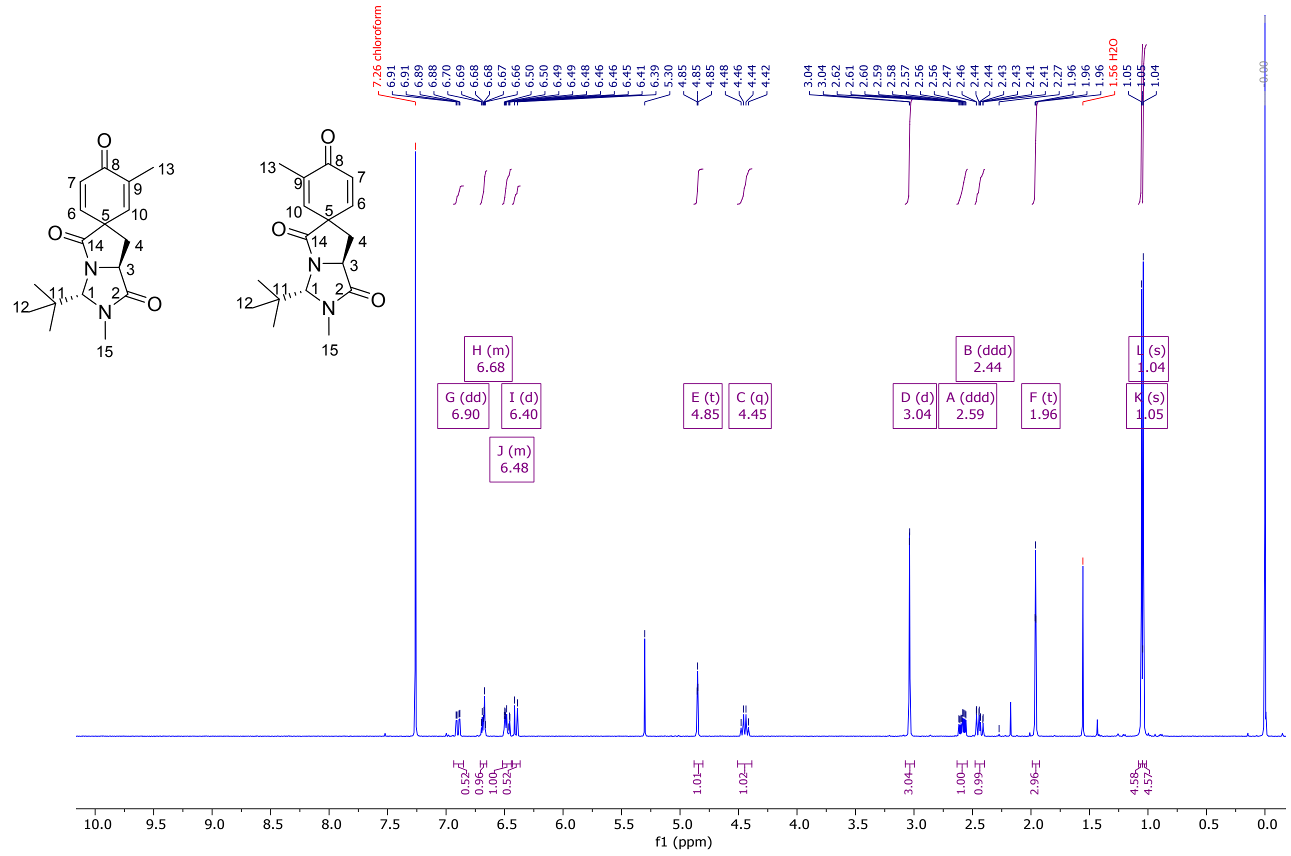

^{1}H NMR of 23

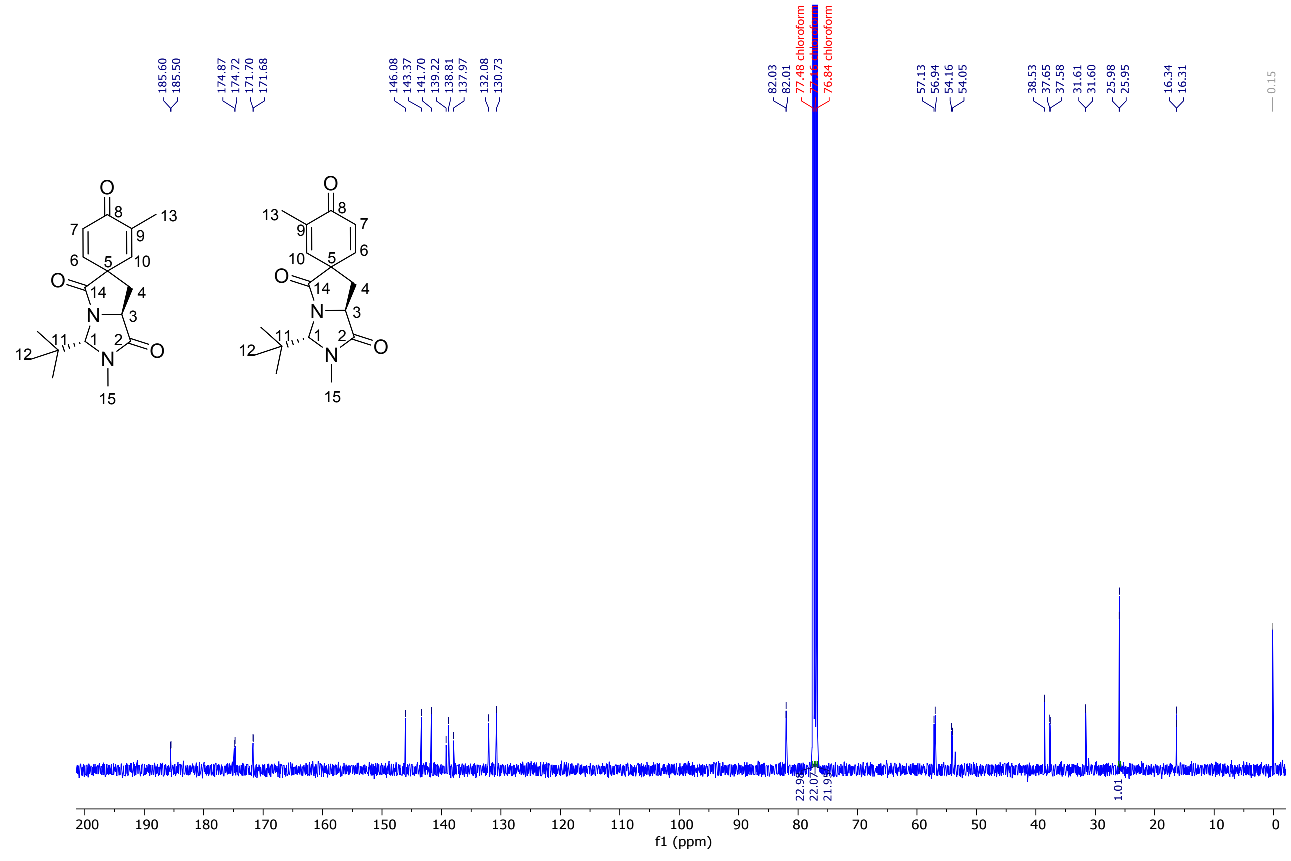

^{13}C NMR of 23

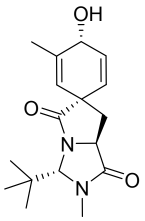

a

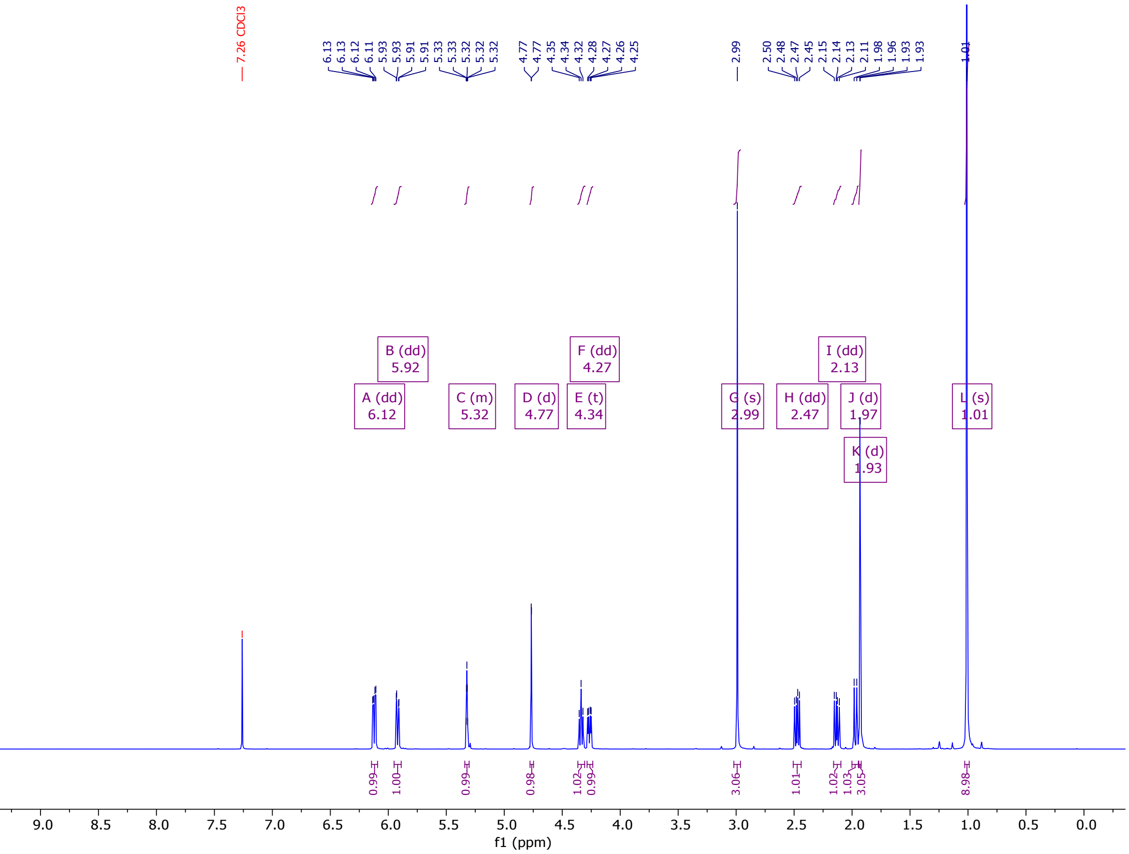

^{1}H NMR of 24a

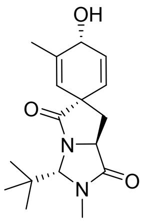

a

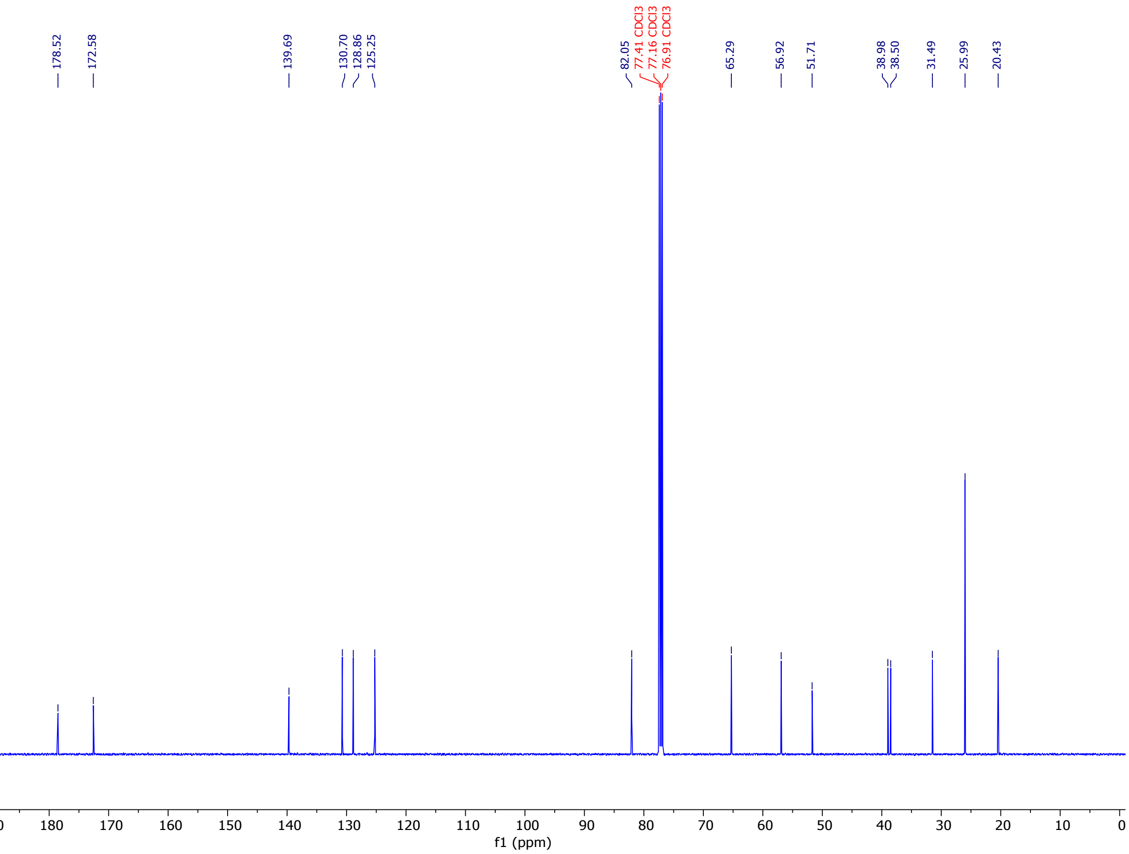

^{13}C NMR of 24a

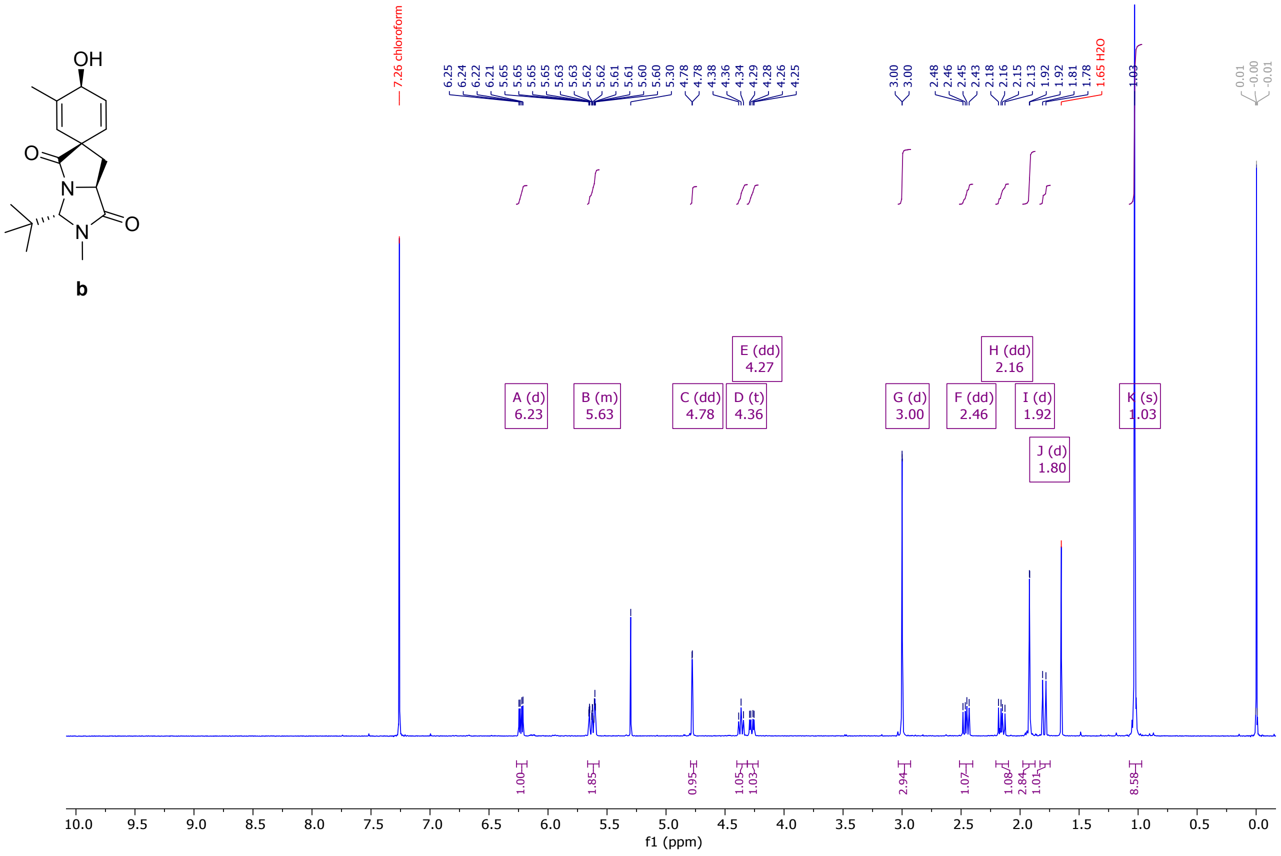

^{1}H NMR of 24b

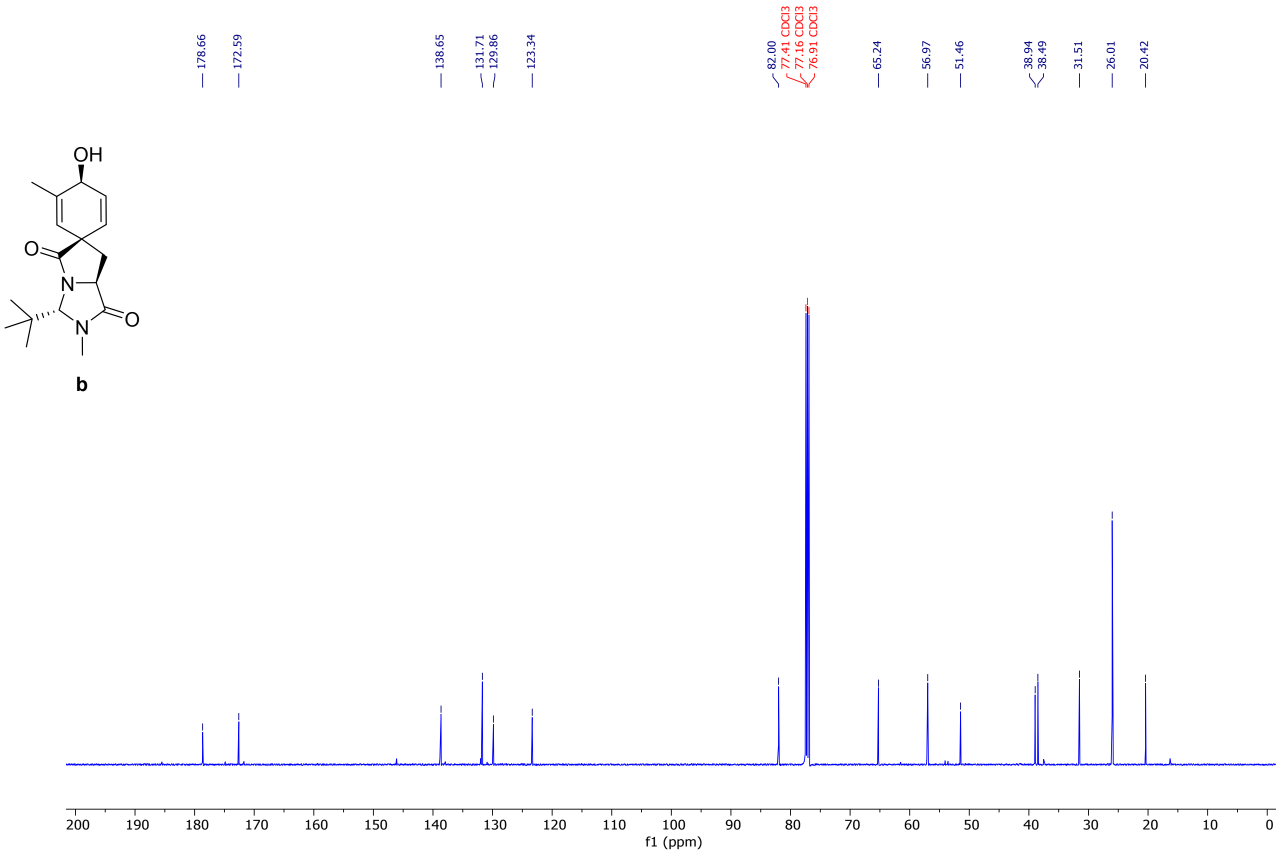

^{13}C NMR of 24b

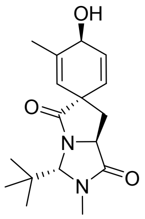

c

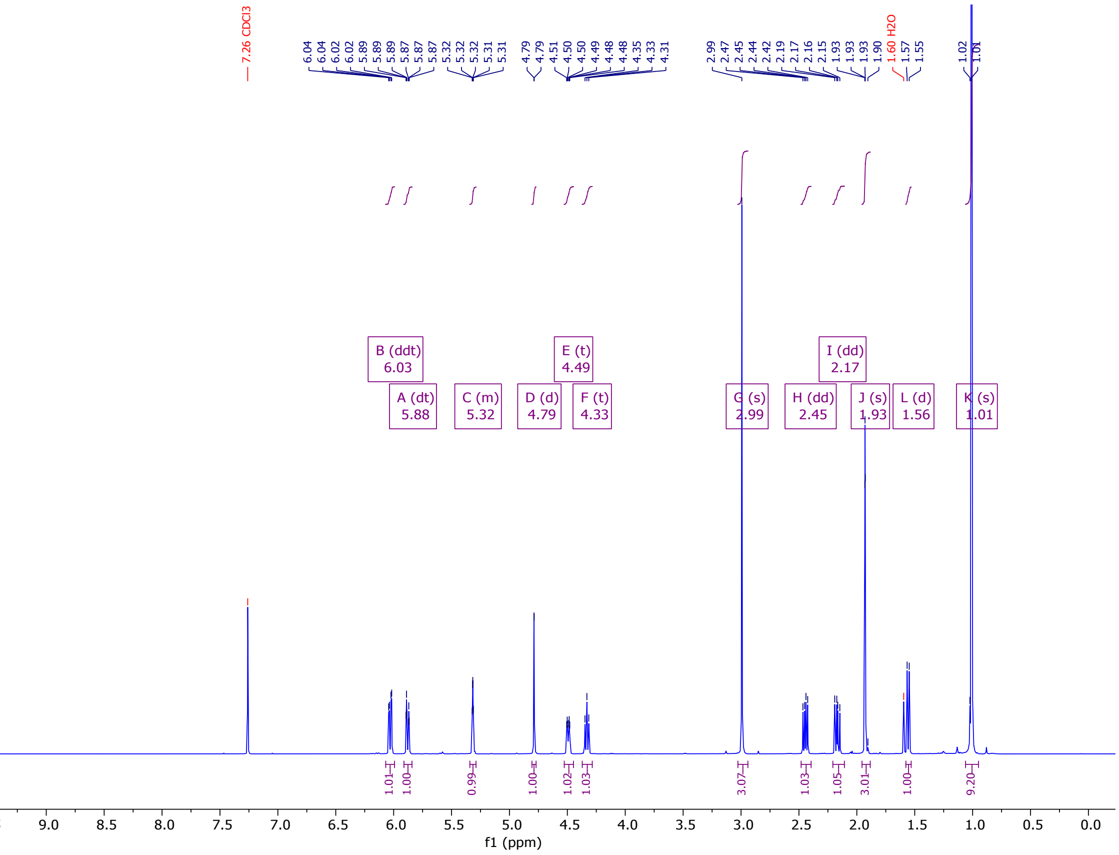

^{1}H NMR of 24c

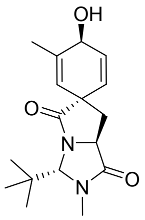

c

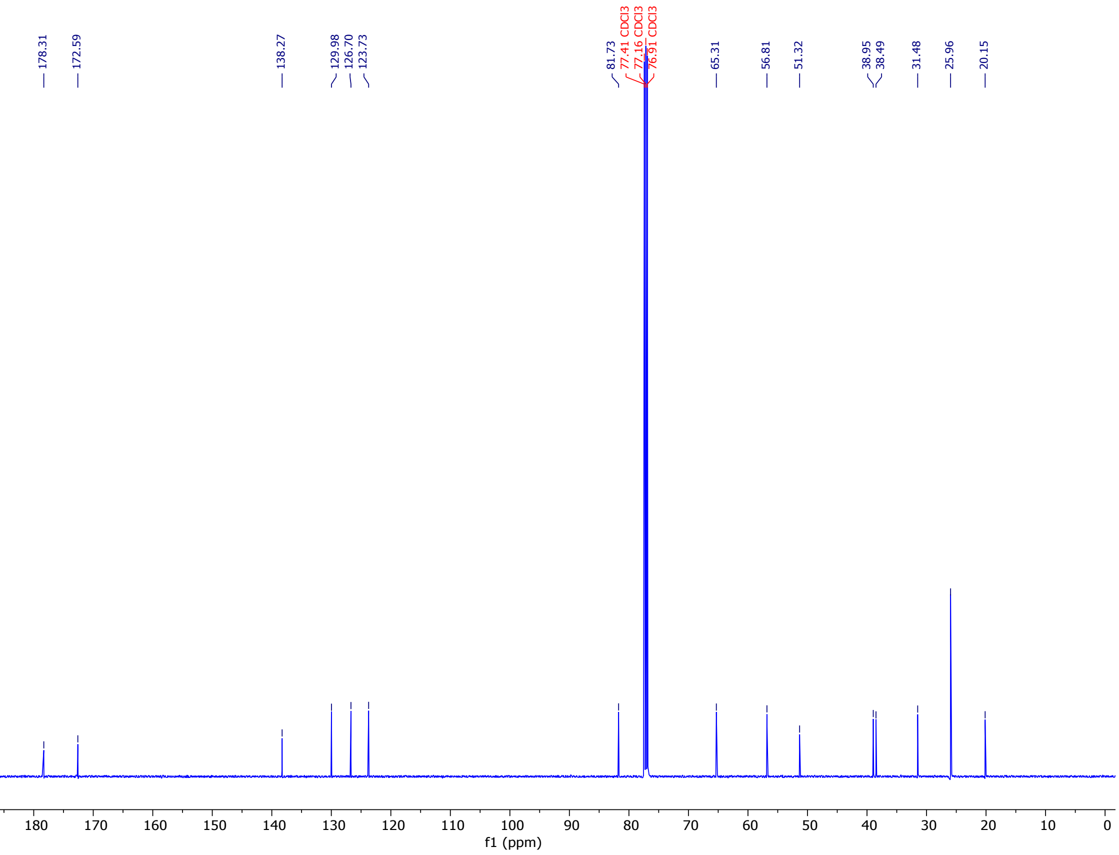

^{13}C NMR of 24c

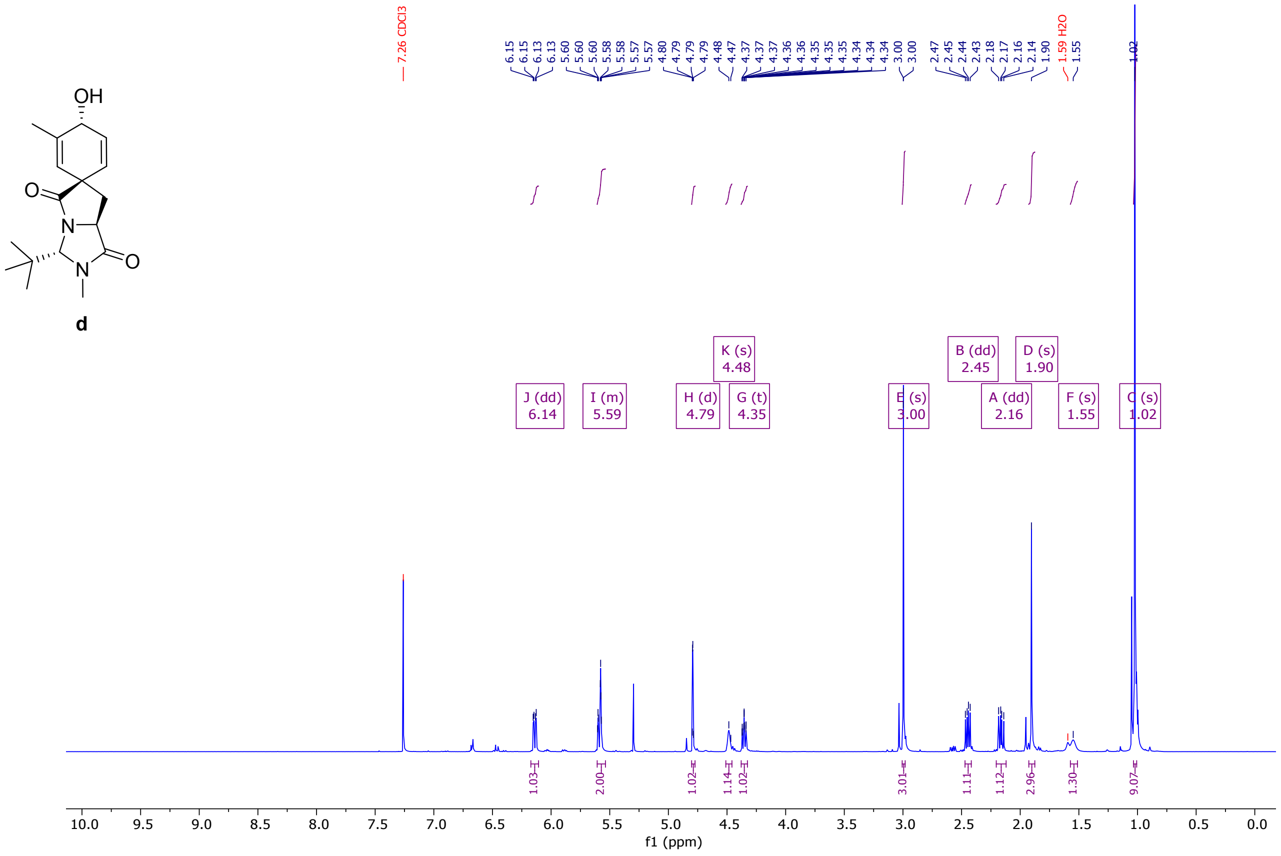

^{1}H NMR of 24d

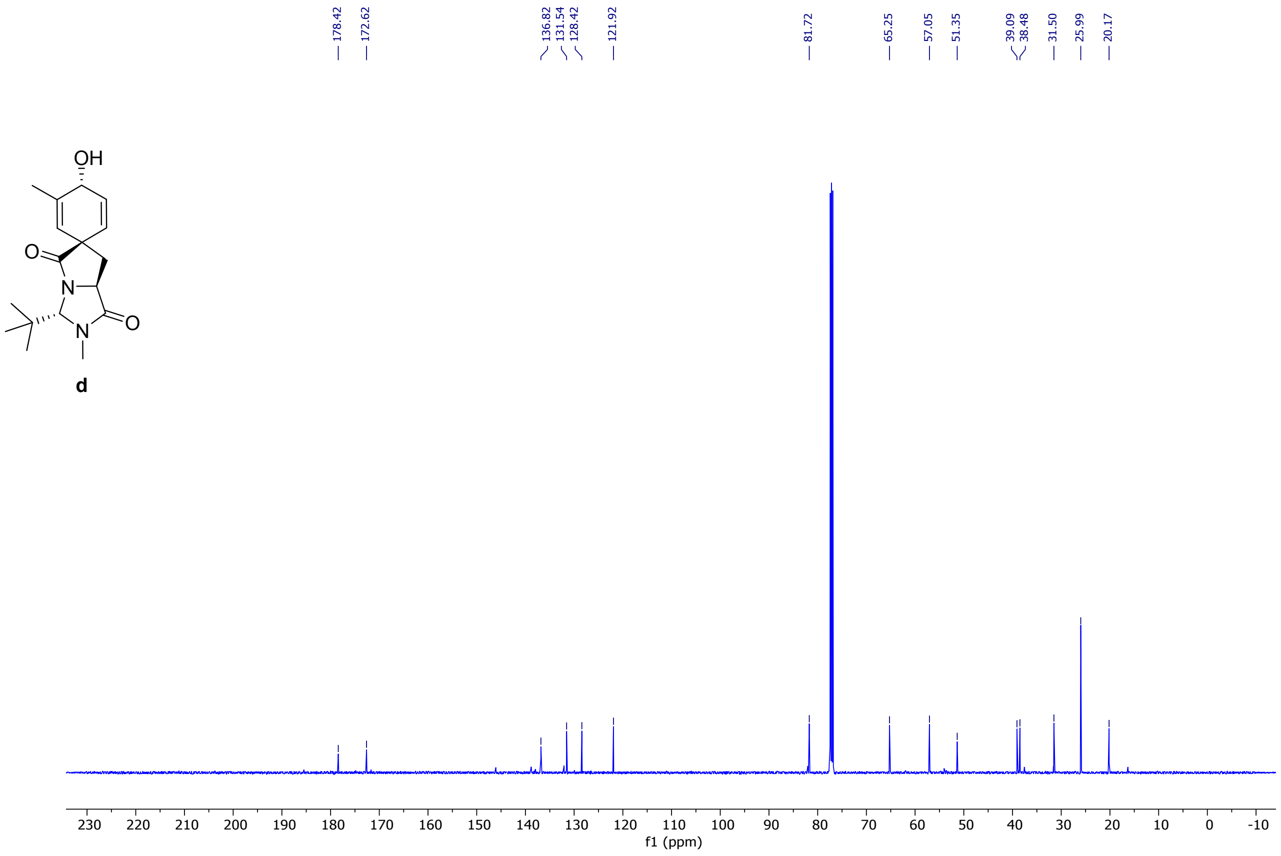

^{13}C NMR of 24d

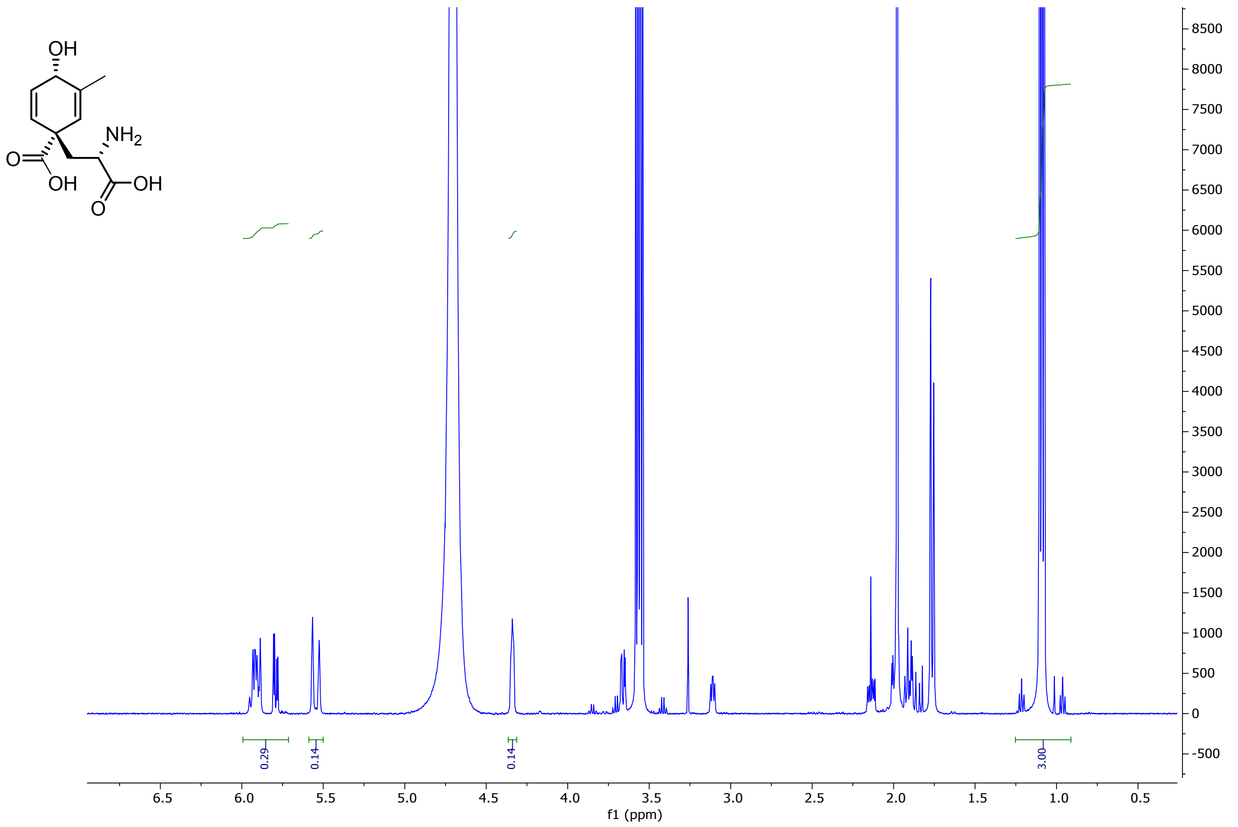

Quantitative ^{1}H NMR of 26

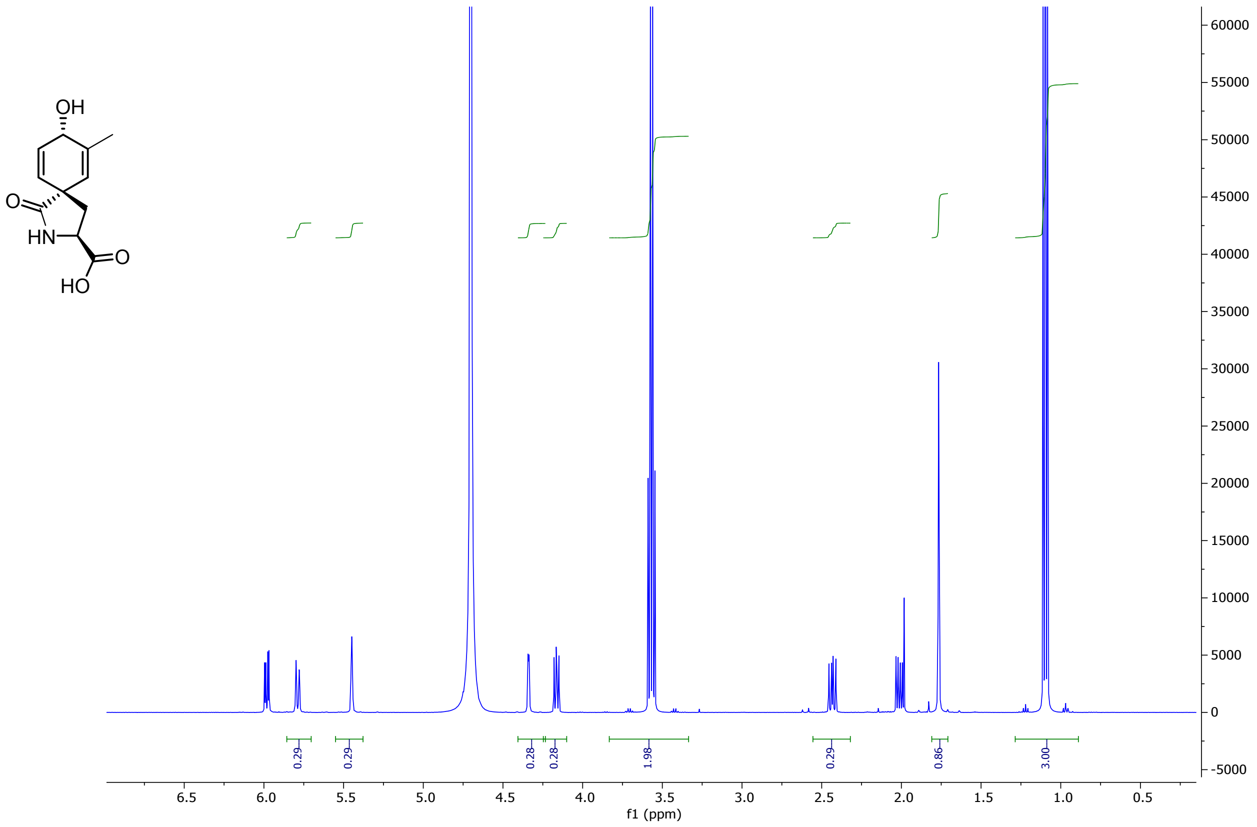

Quantitative ^{1}H NMR of 25

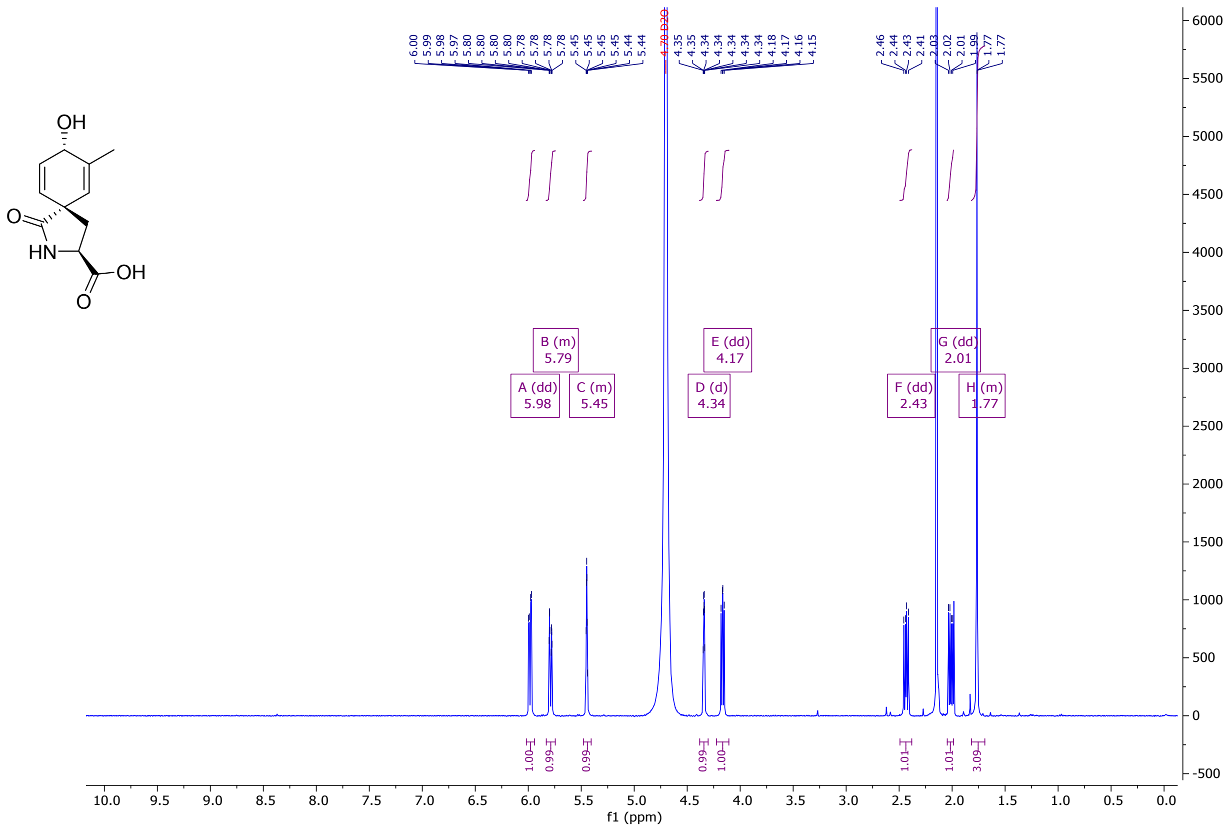

^{1}H NMR of 25

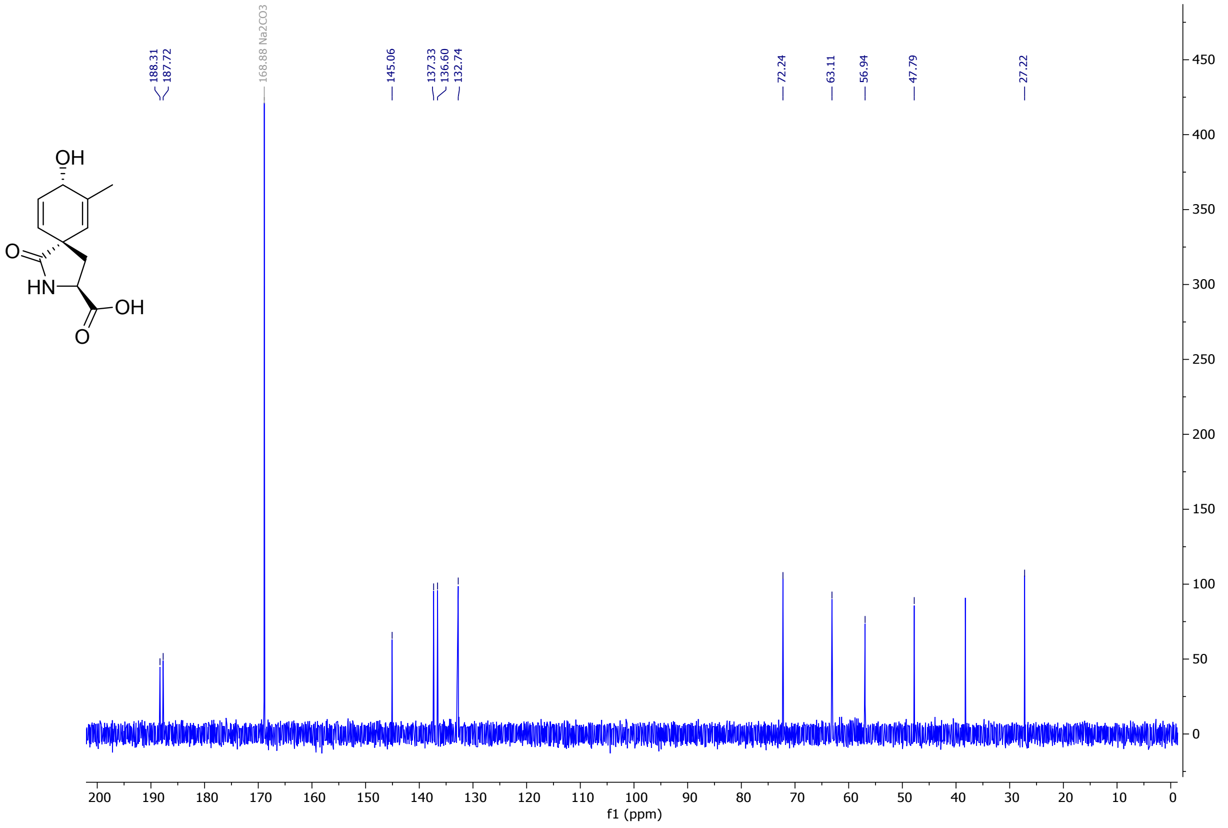

^{13}C NMR of 25 (referenced to sodium carbonate)[4]

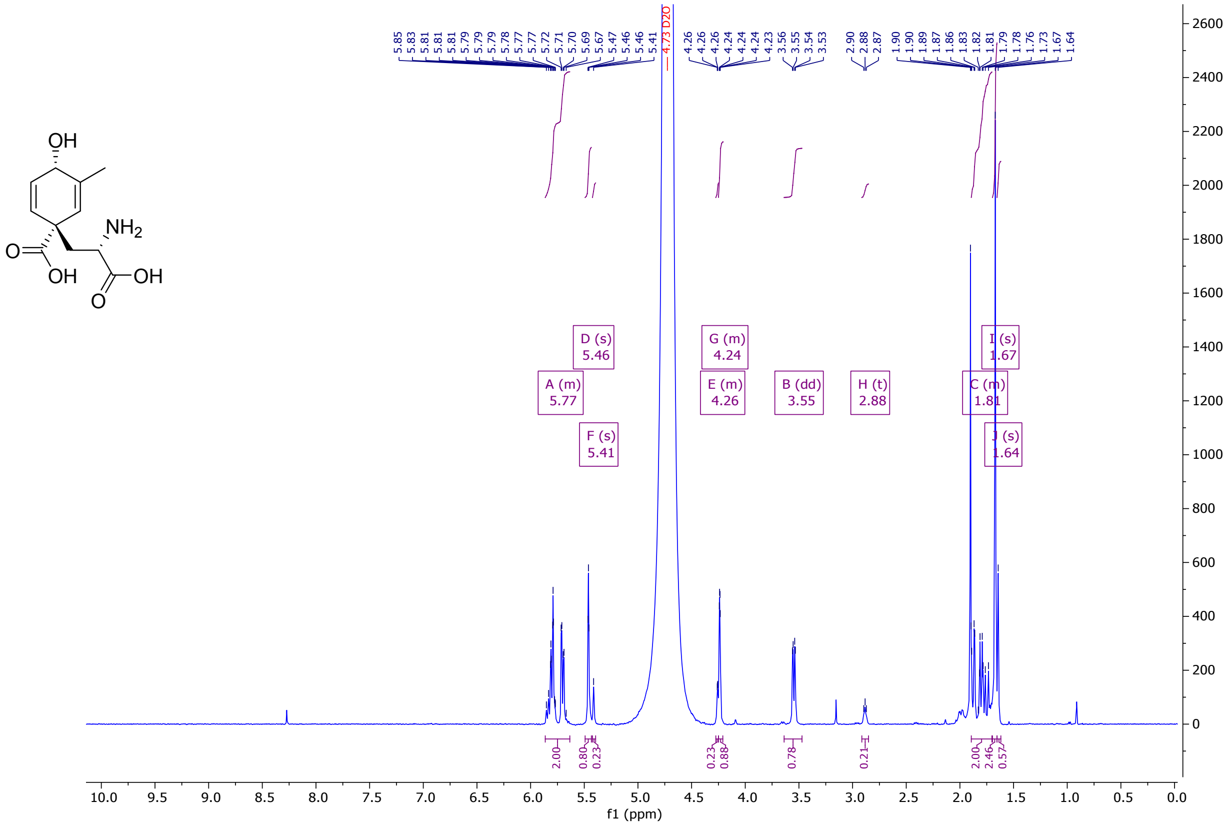

^{1}H NMR of 26

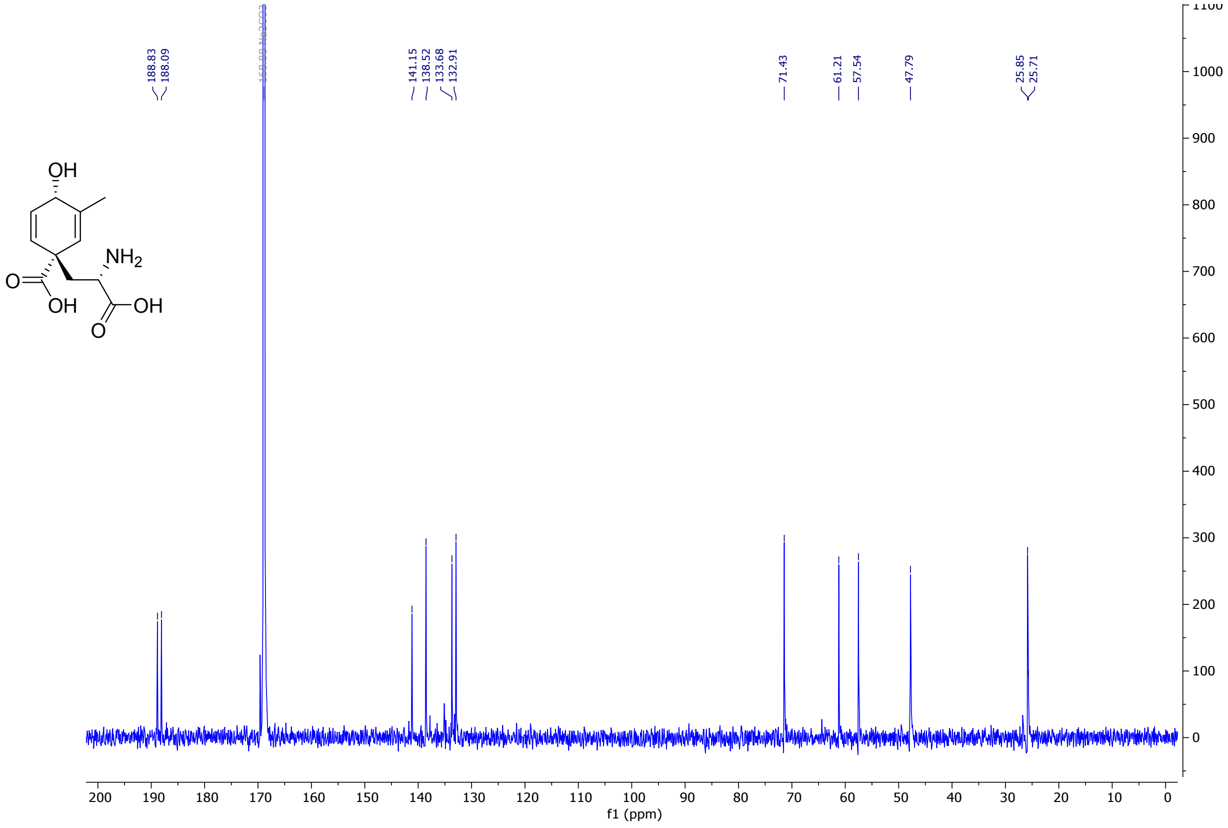

^{13}C NMR of 26 (referenced to sodium carbonate)[4]

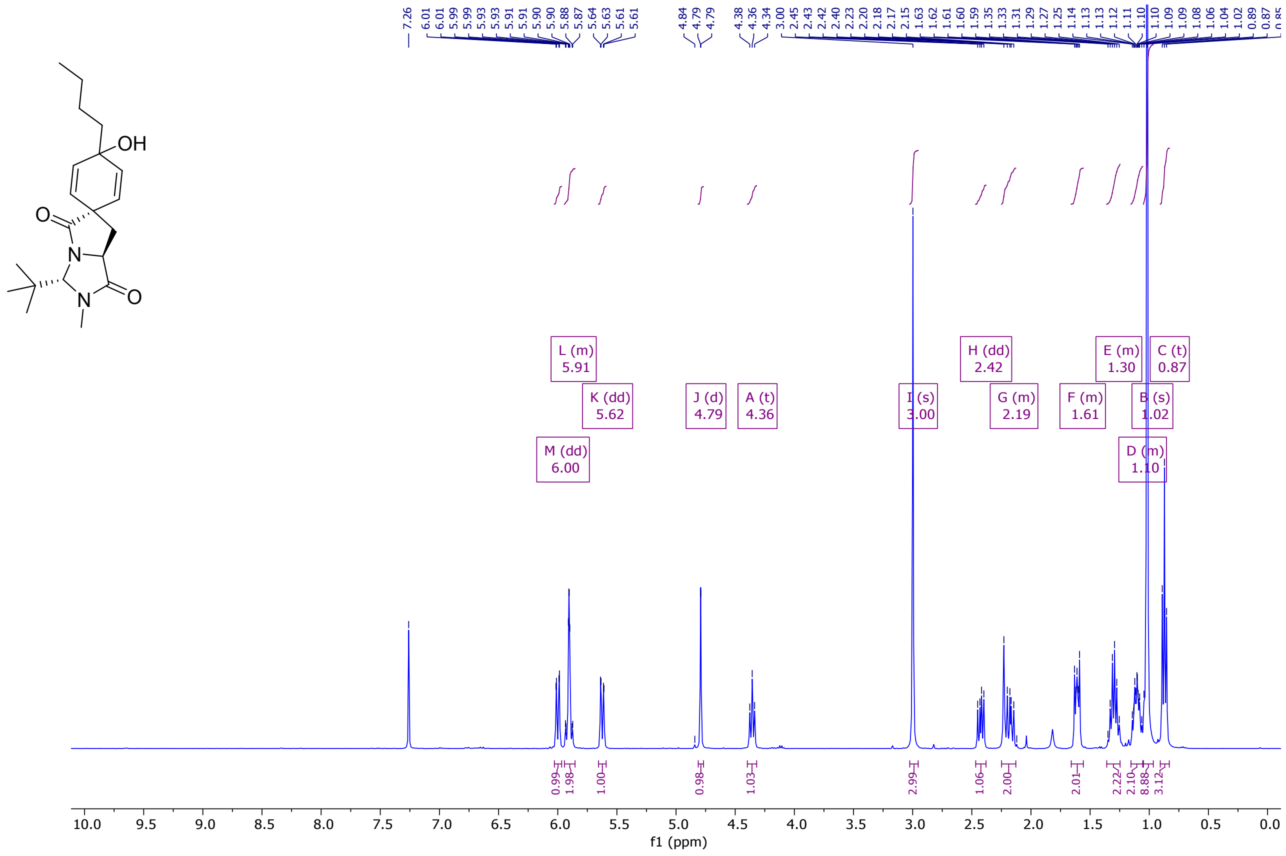

^{1}H NMR of 16

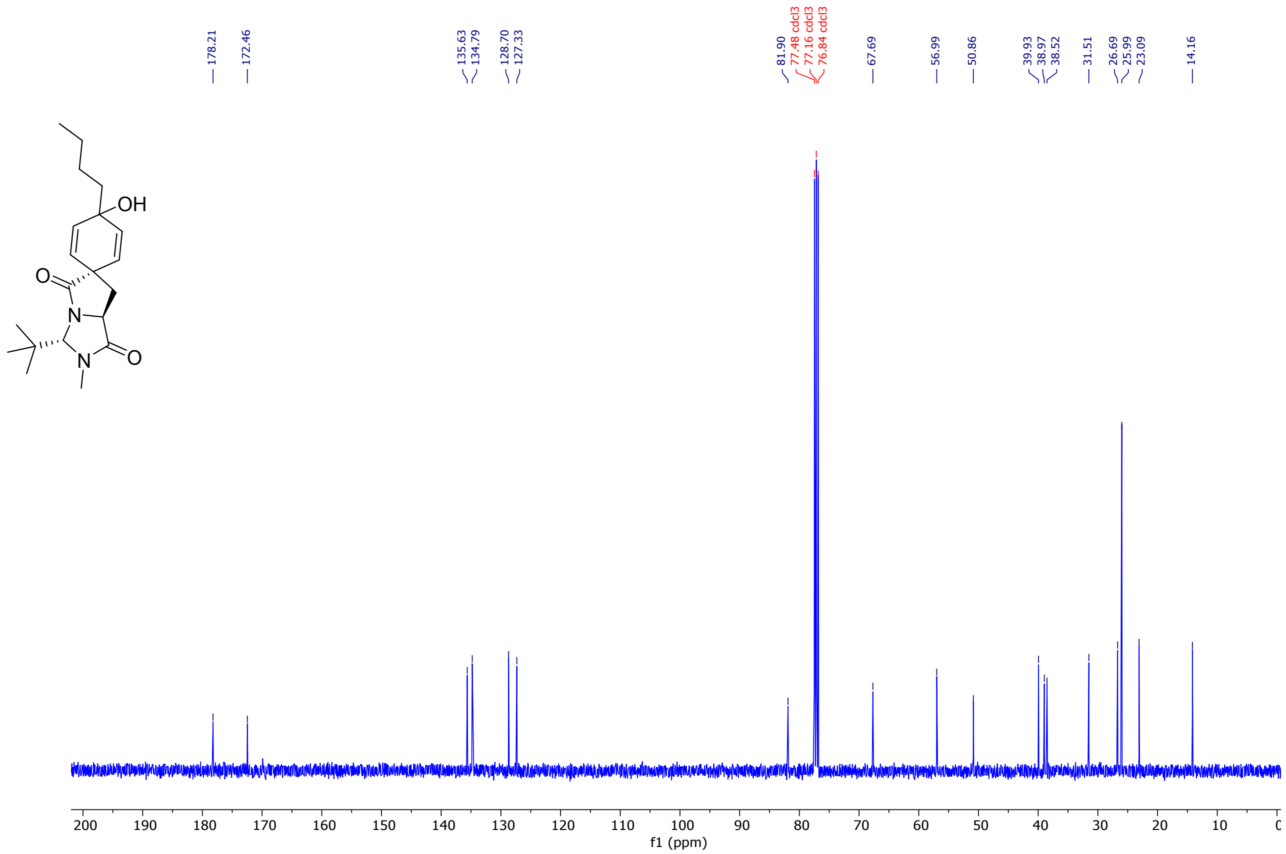

^{13}C NMR of 16

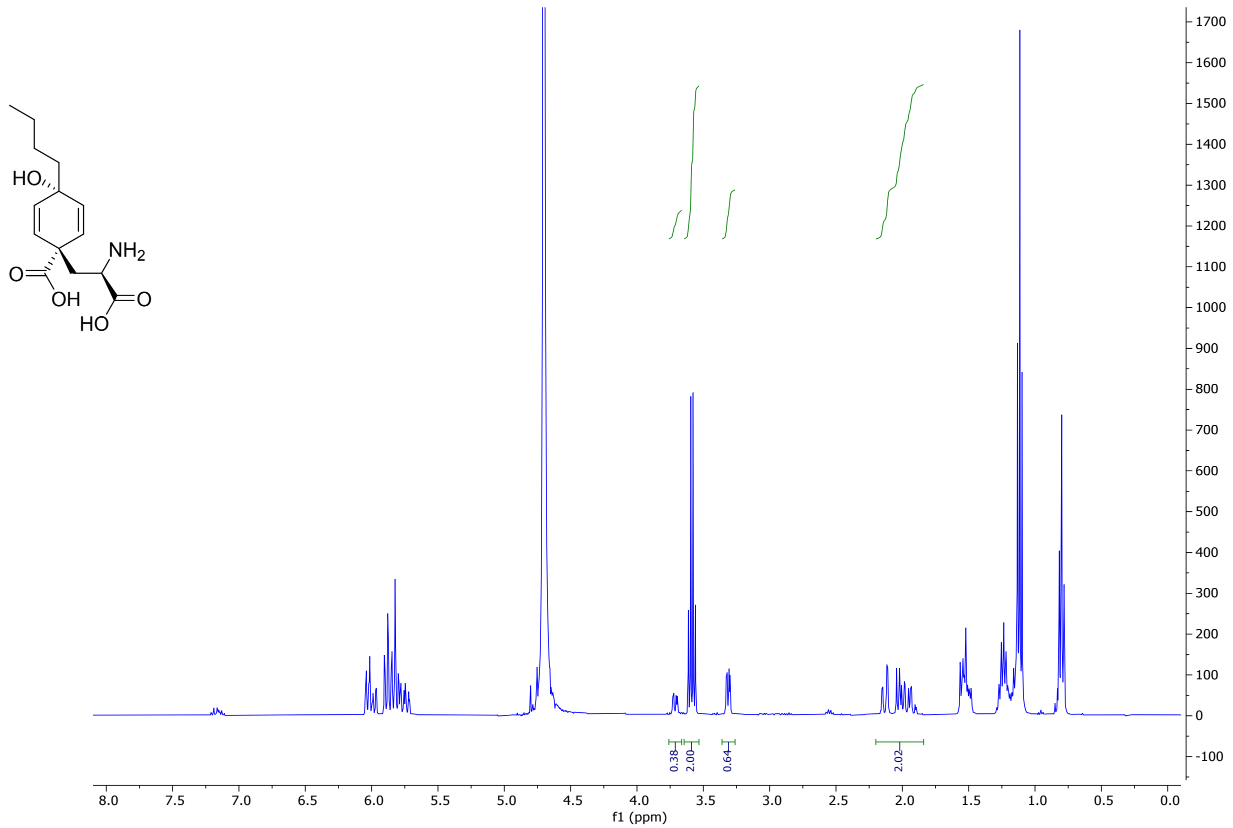

Quantitative ^{1}H NMR of 18

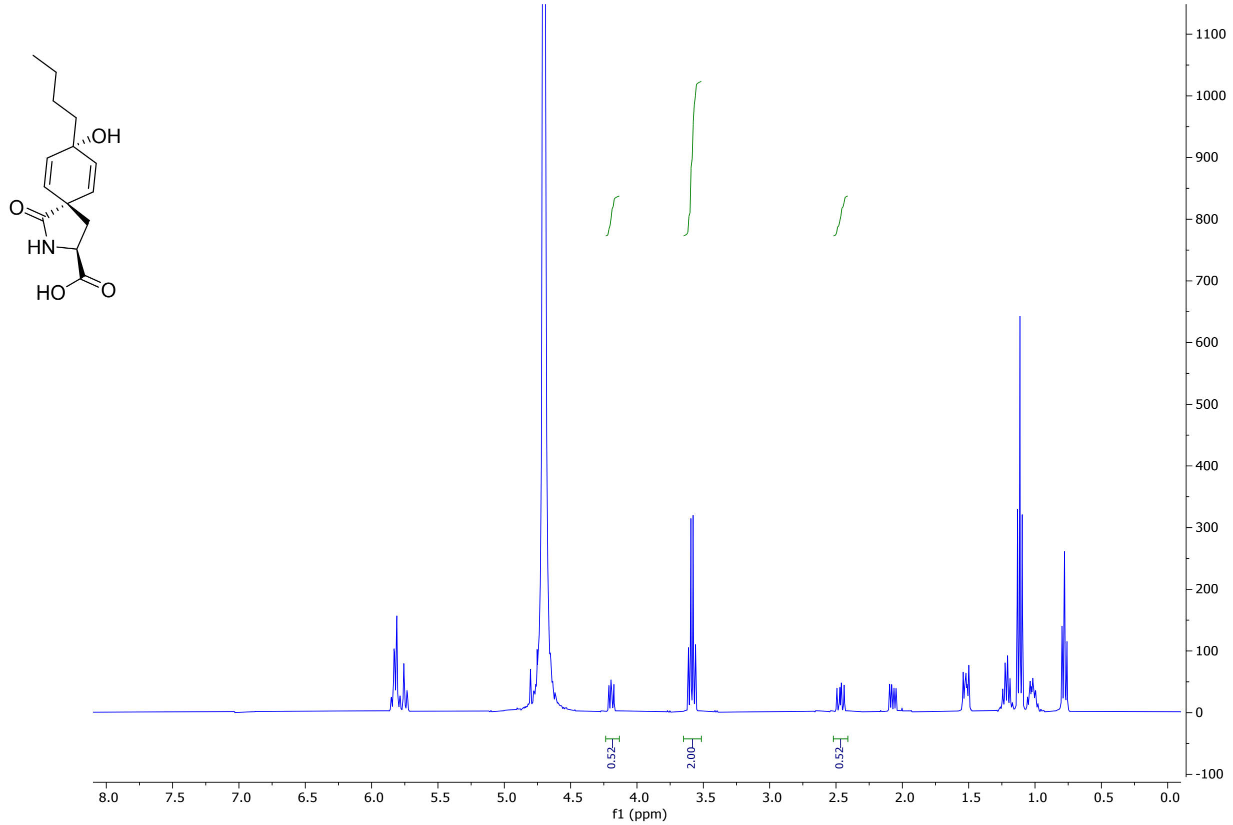

Quantitative ^{1}H NMR of S7

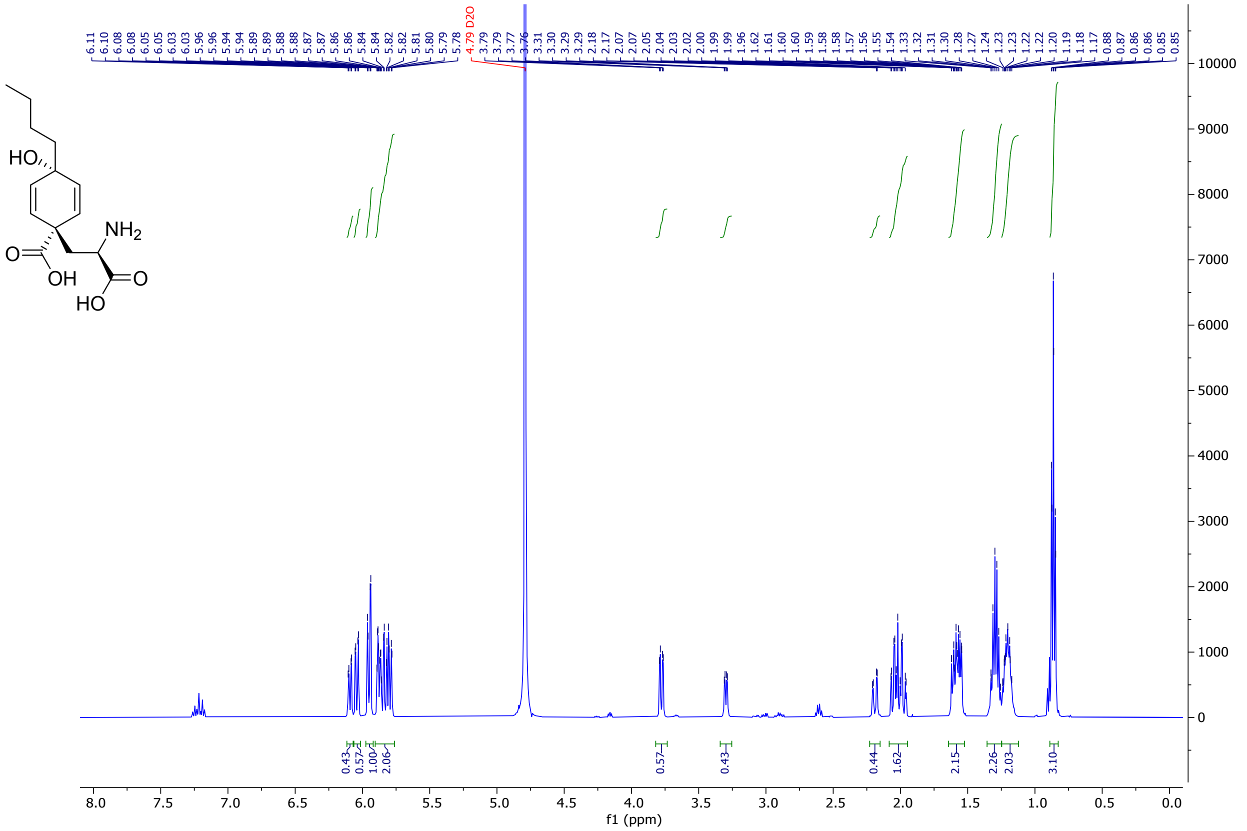

¹H NMR of 18

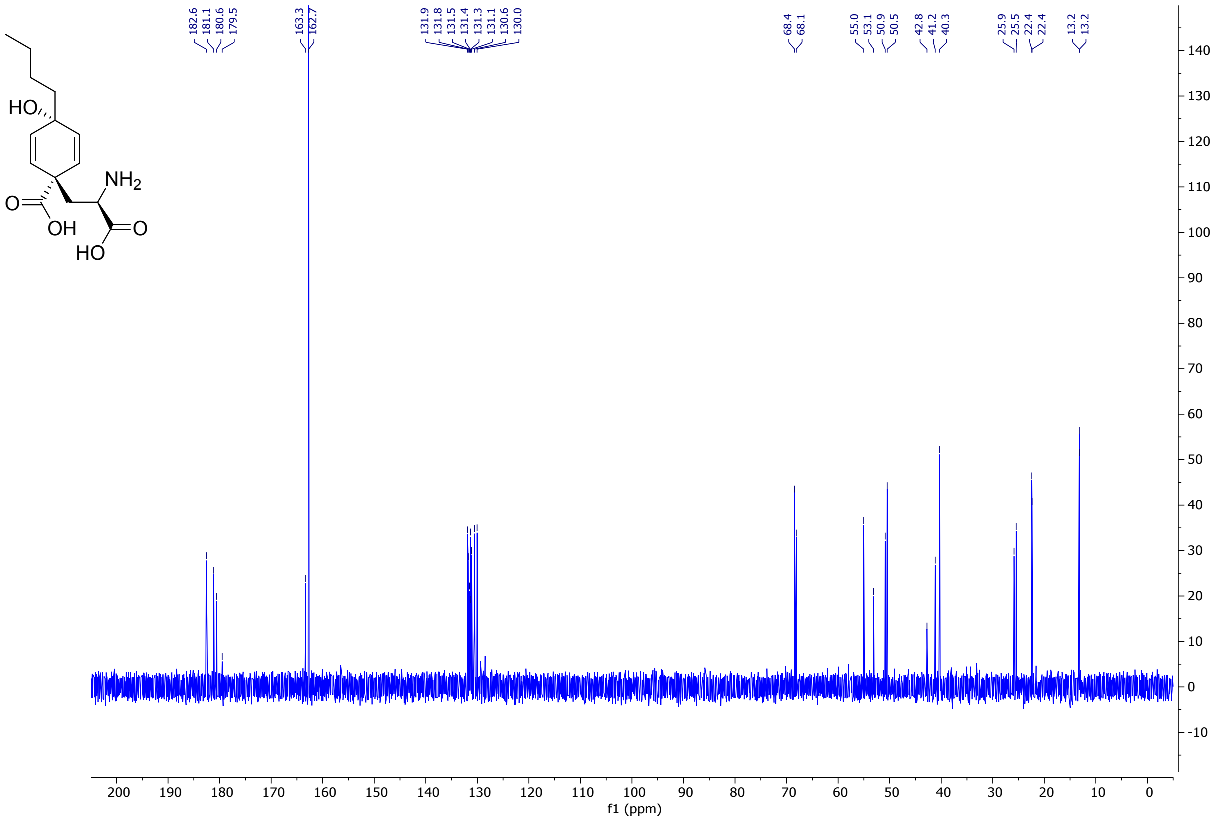

¹³C NMR of 18

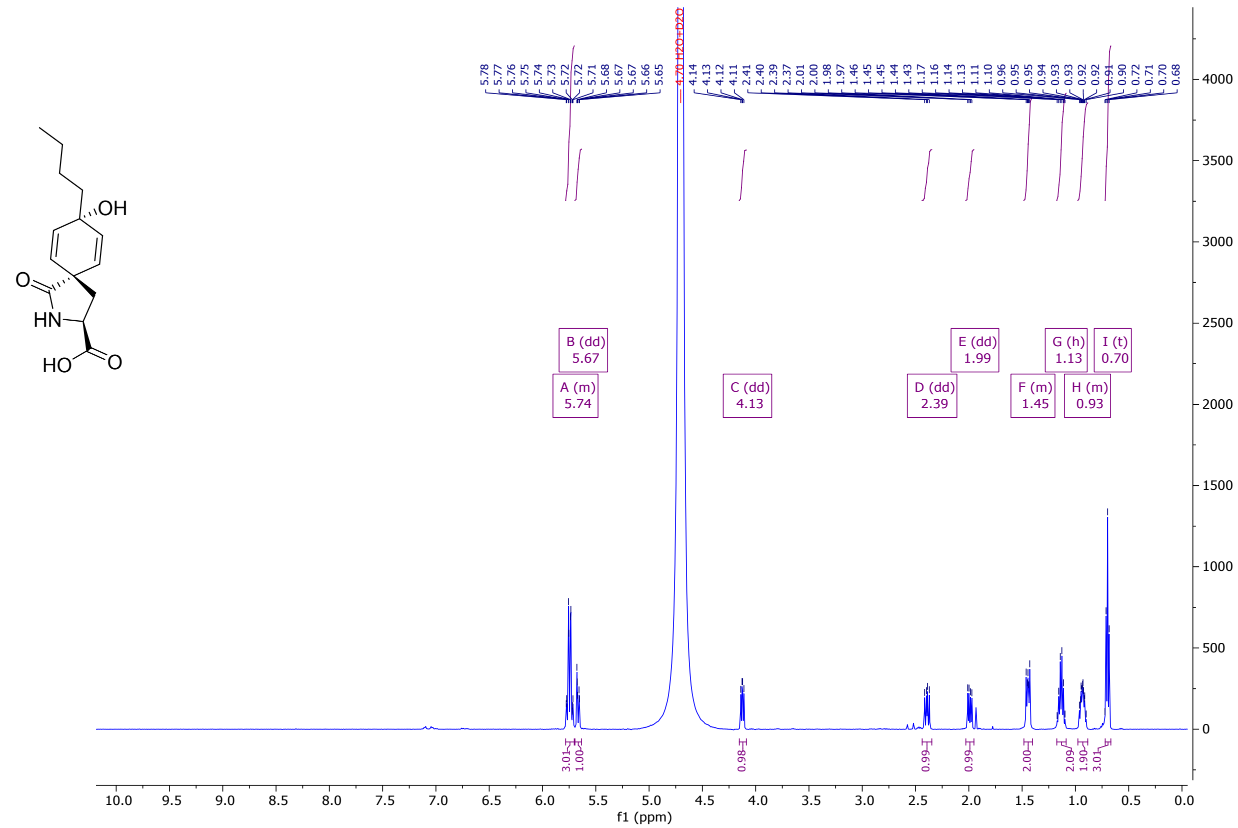

^{1}H NMR of S7

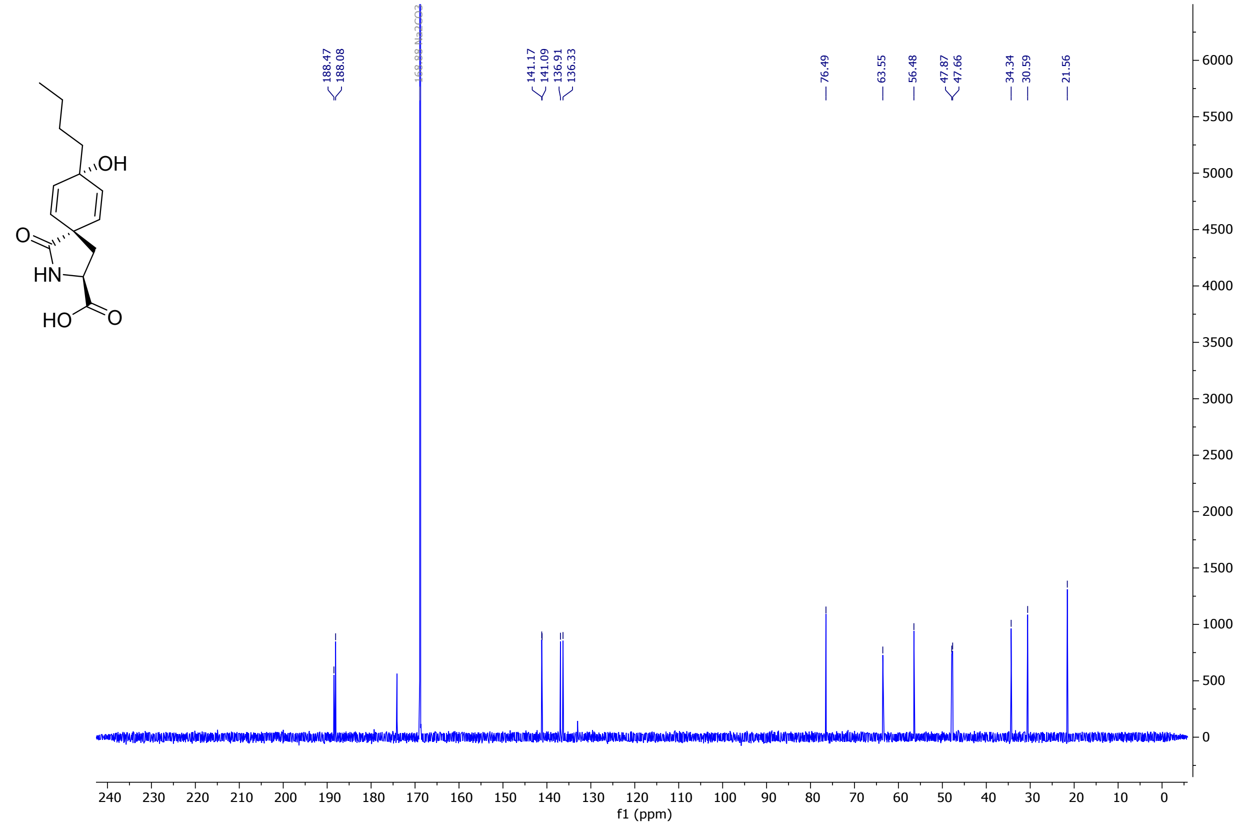

^{13}C NMR of S7 (referenced to sodium carbonate)[4]

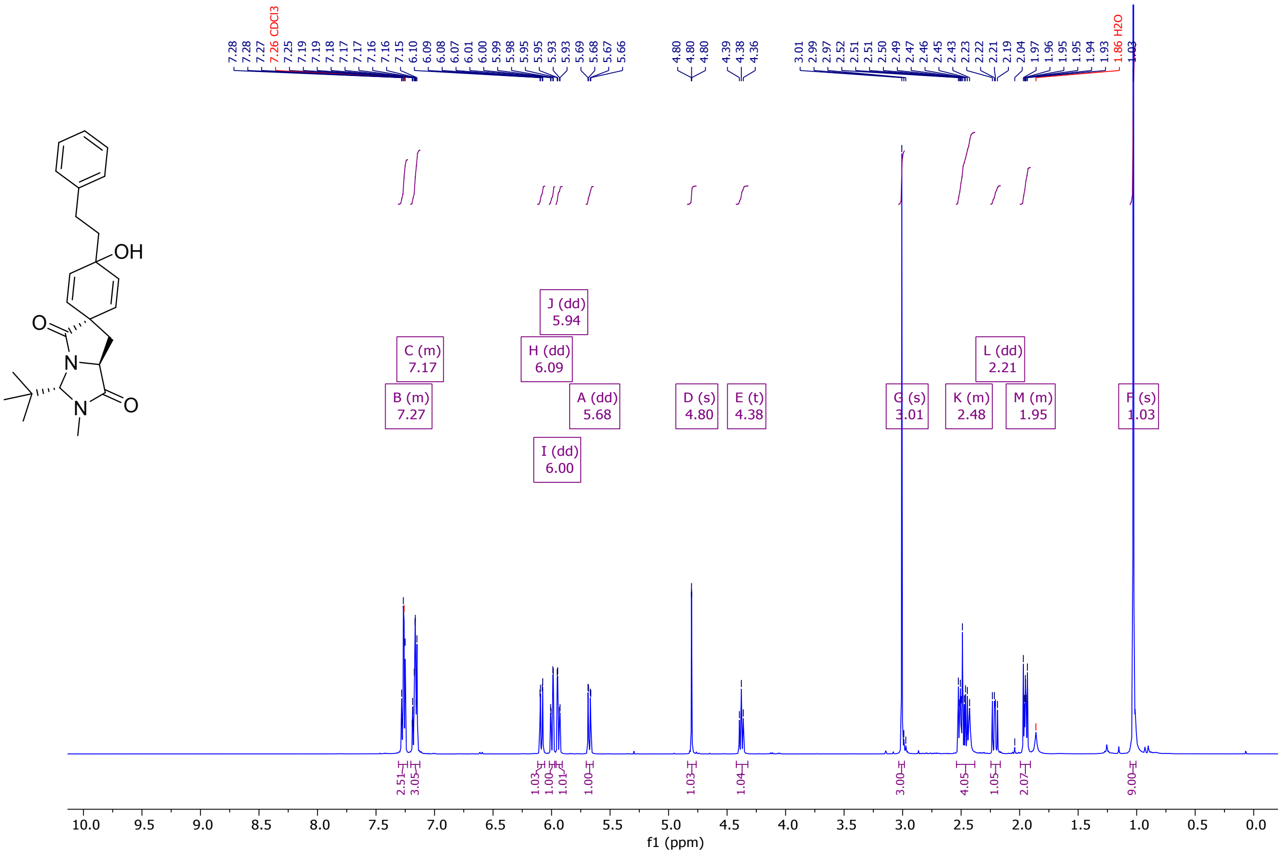

^{1}H NMR of 17

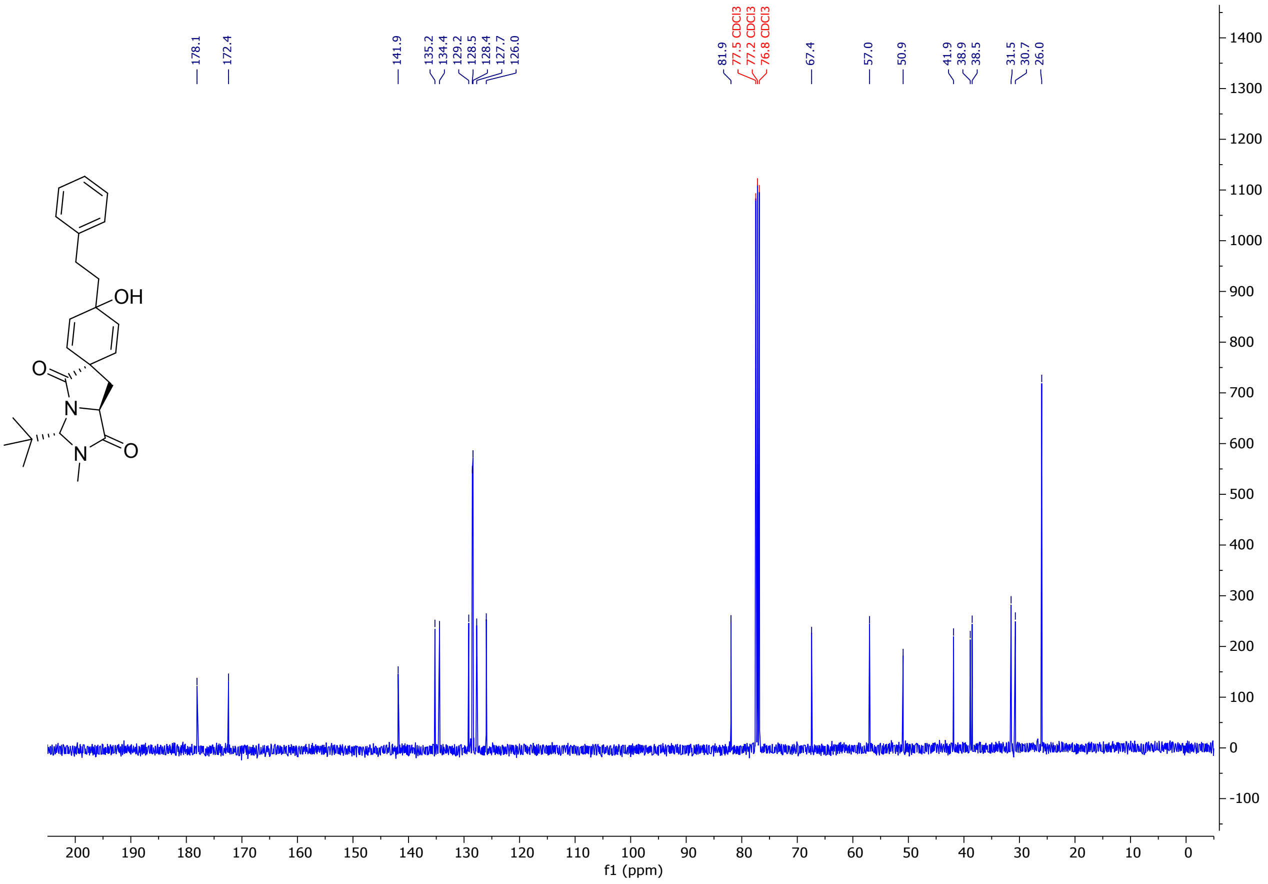

^{13}C NMR of 17

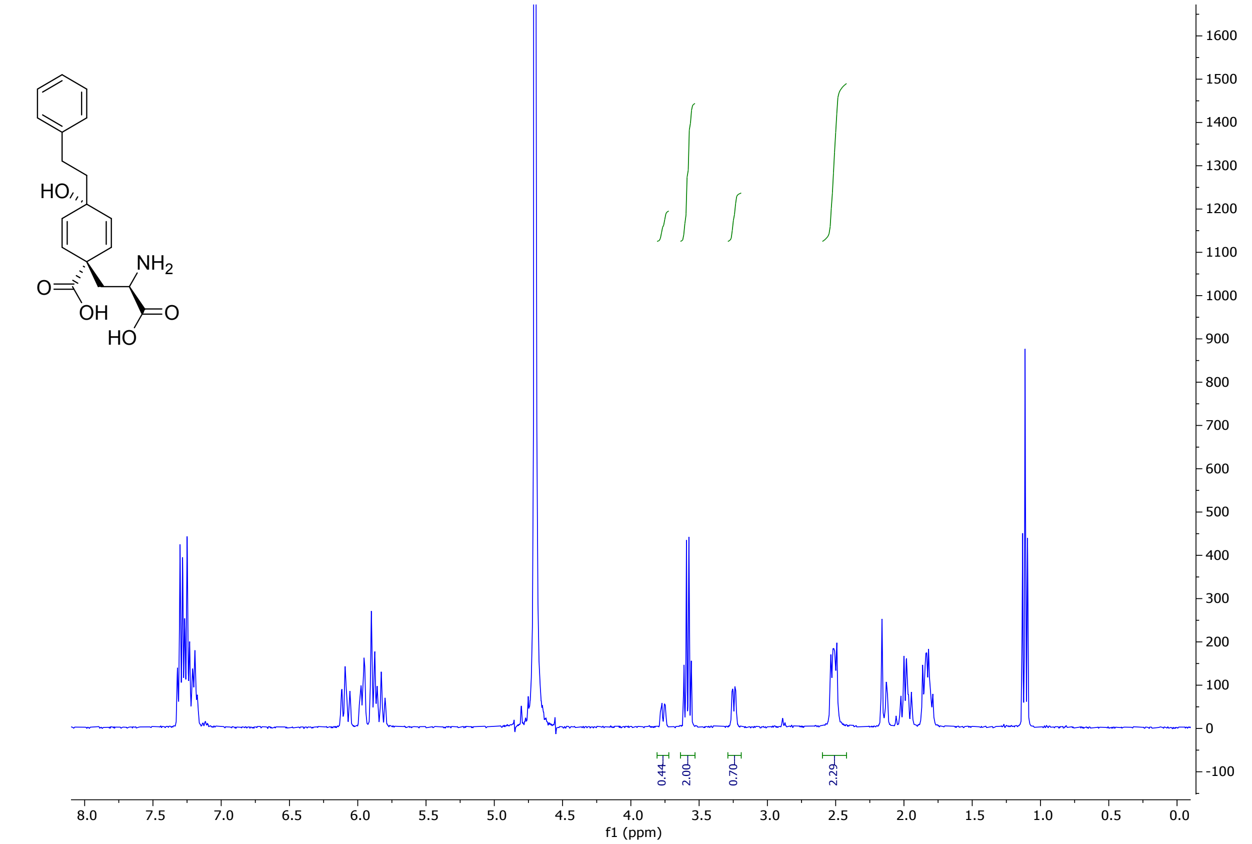

Quantitative ^{1}H NMR of 19

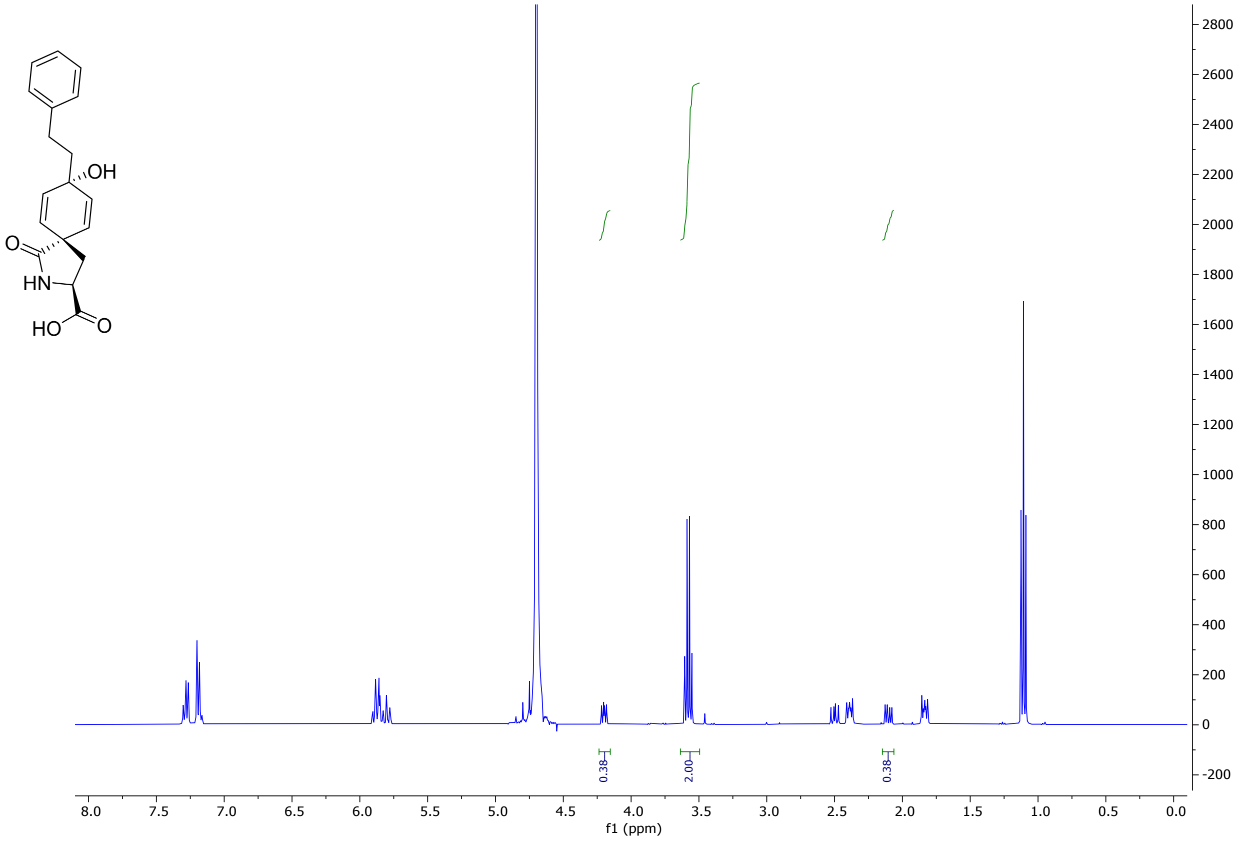

Quantitative ^{1}H NMR of S8

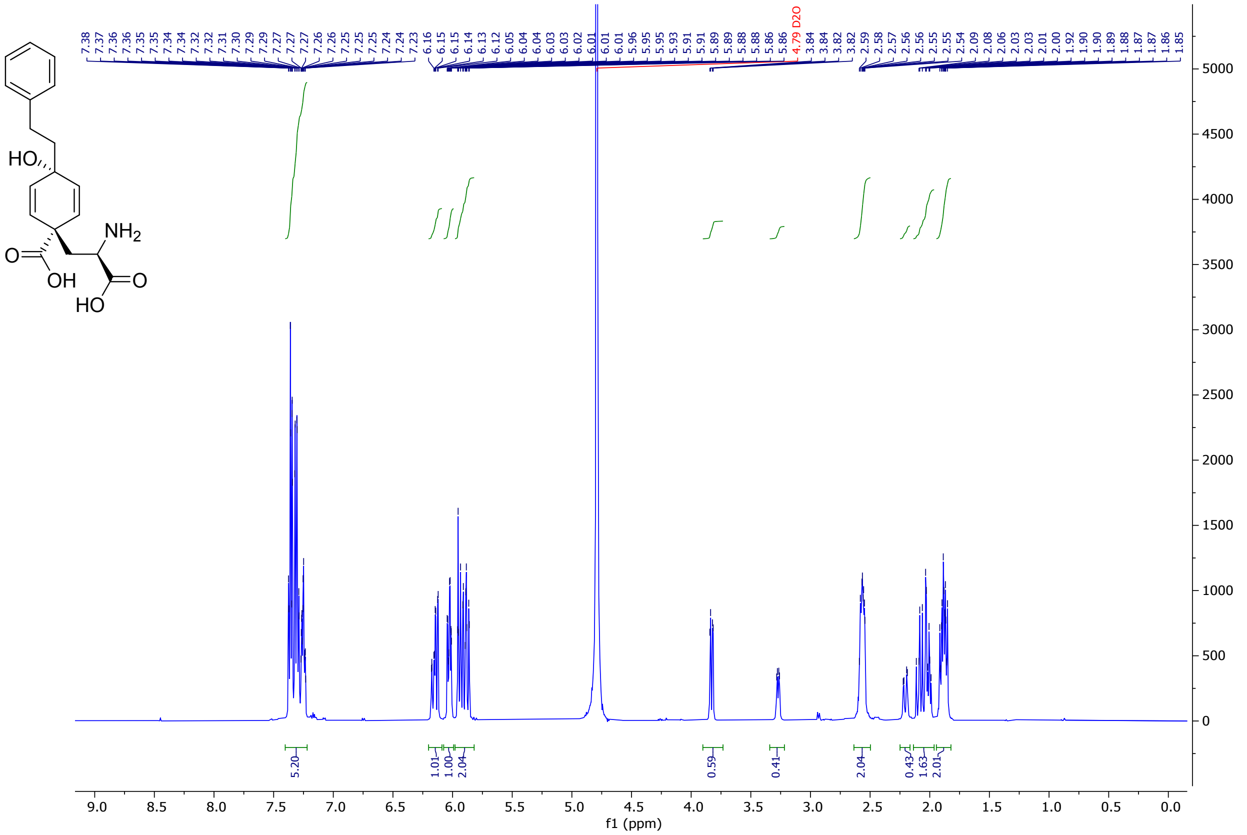

^{1}H NMR of 19

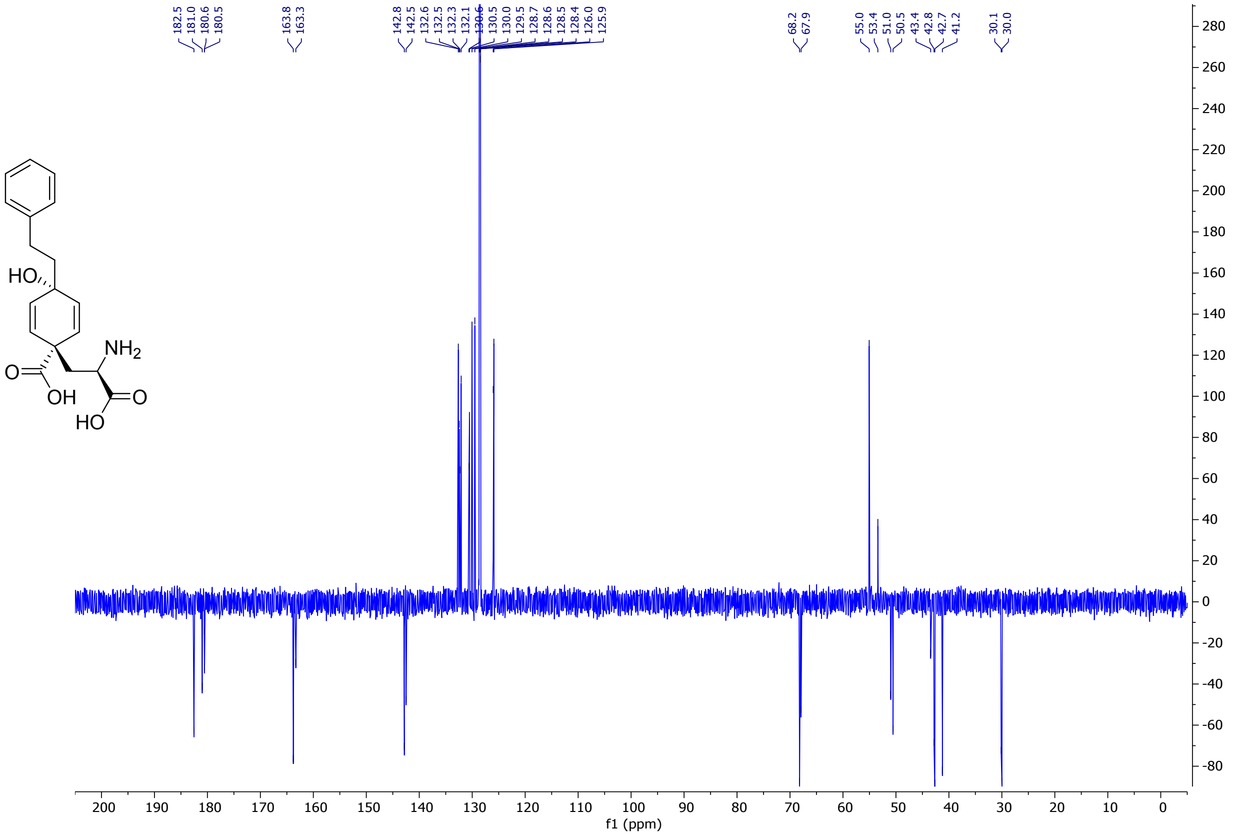

^{13}C NMR of 19

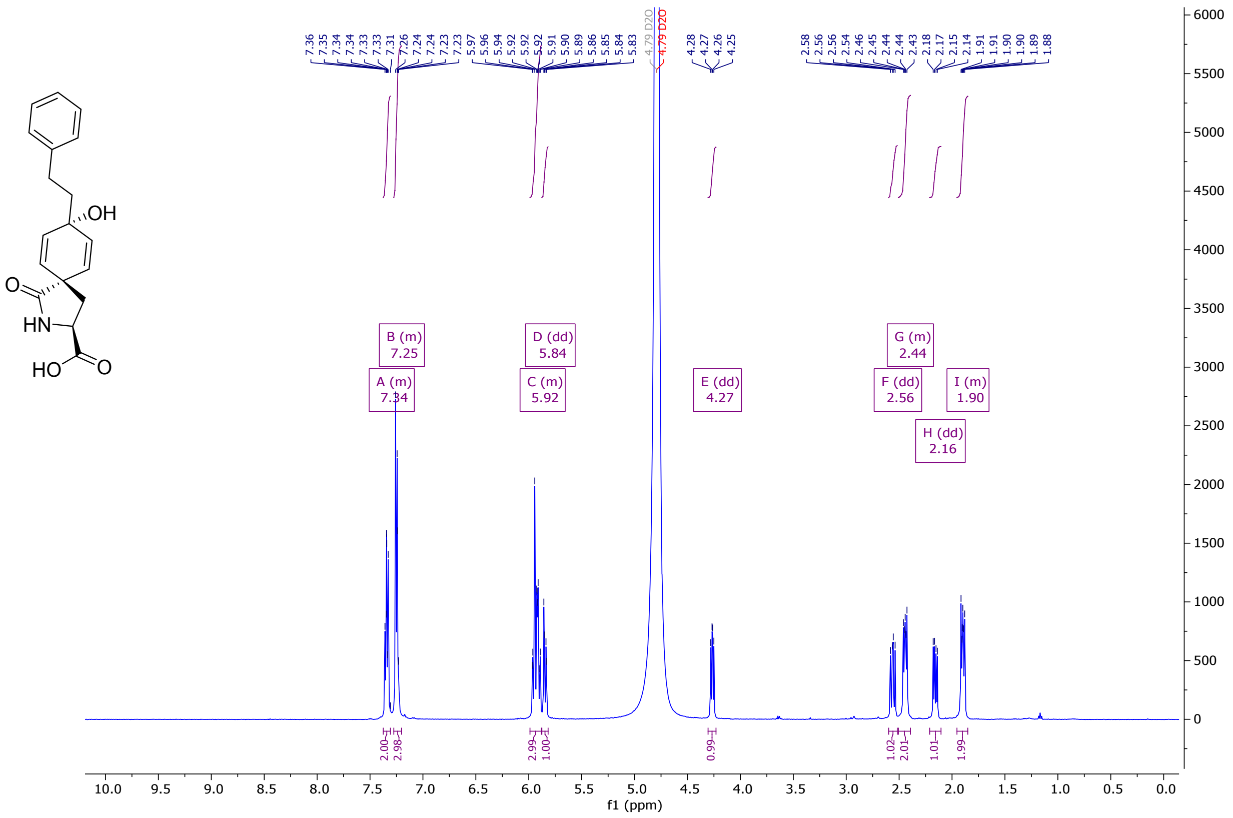

^{1}H NMR of S8

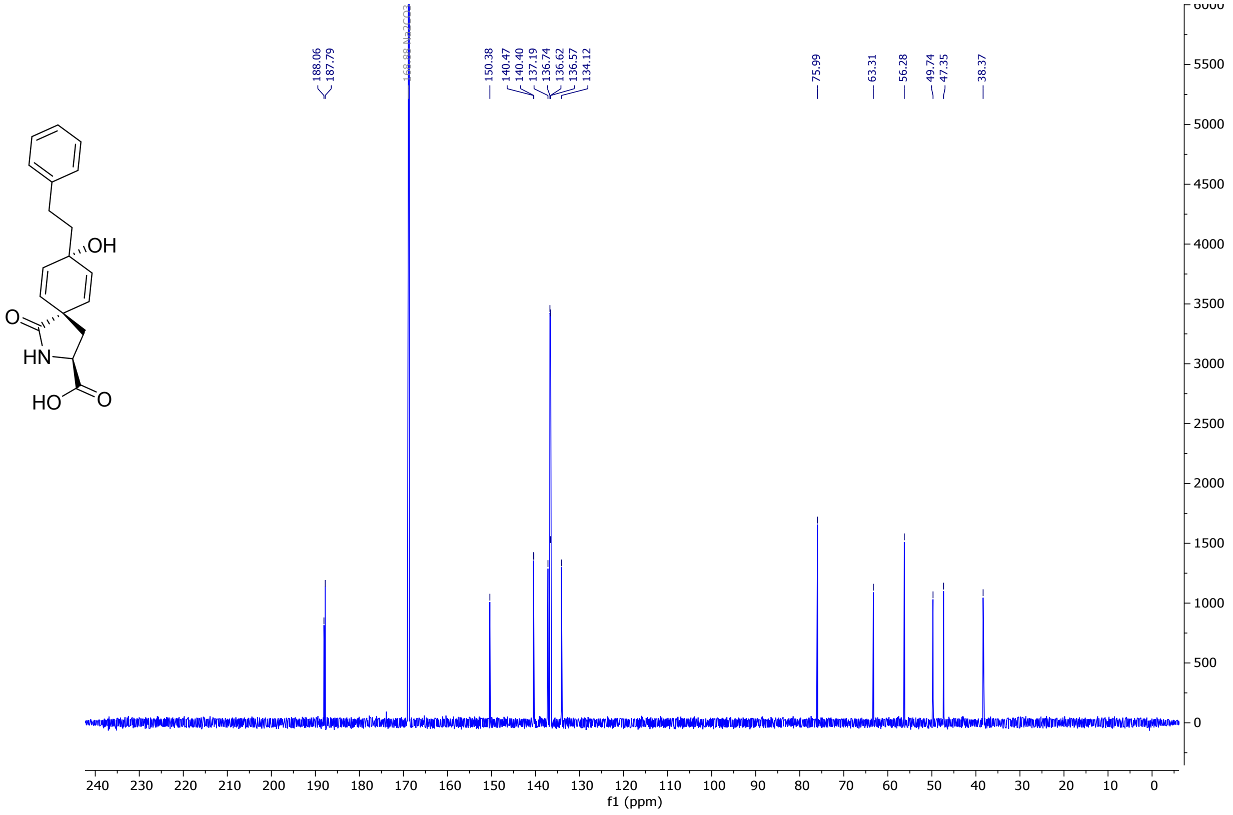

^{13}C NMR of S8 (referenced to sodium carbonate)[4]

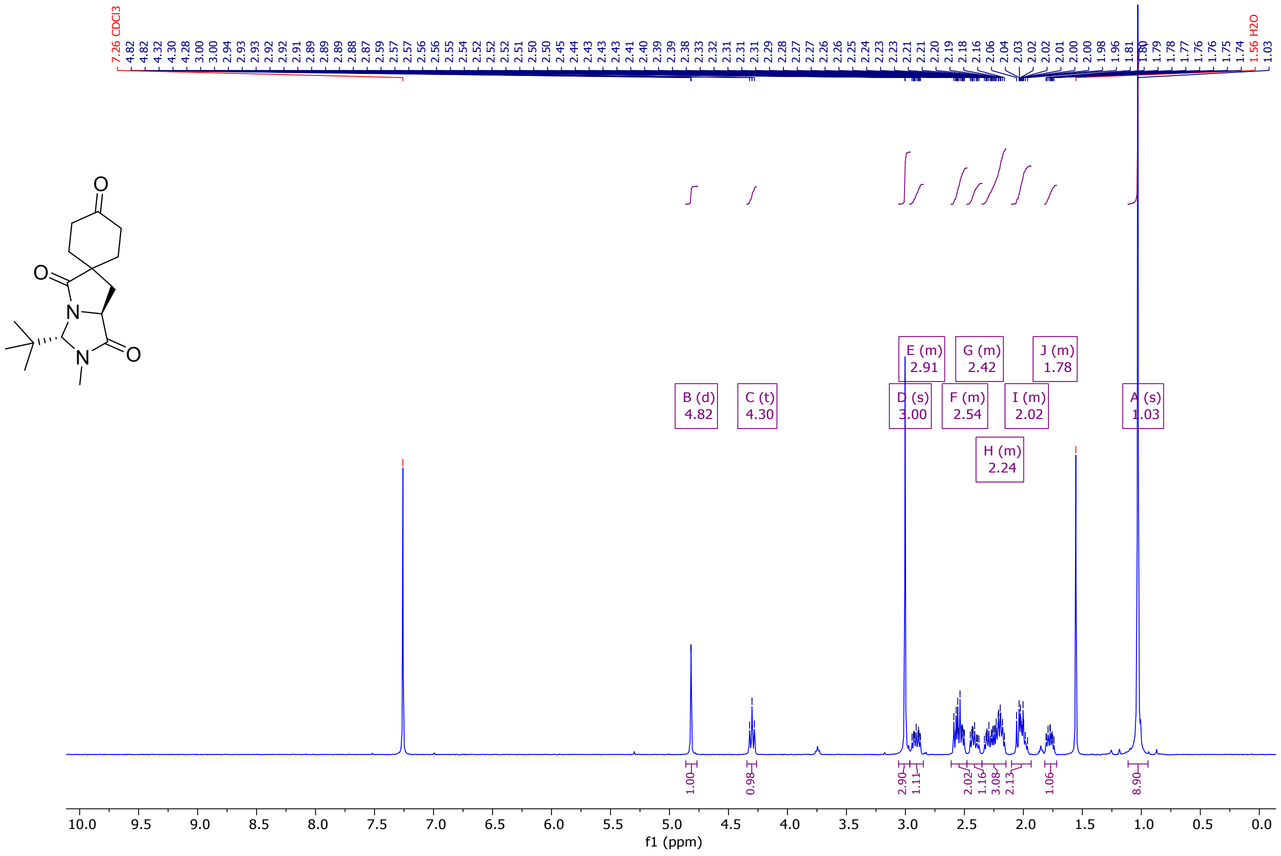

^{1}H NMR of 12

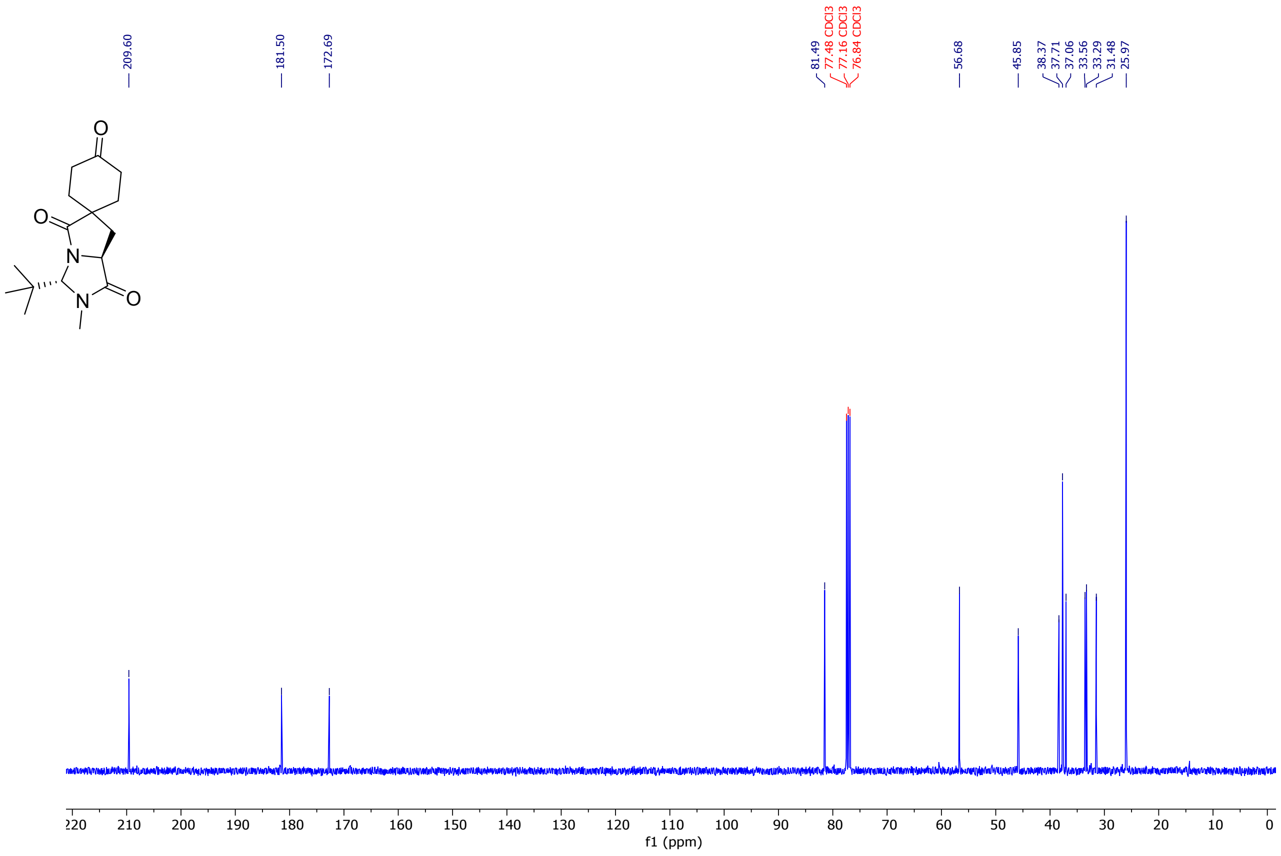

^{13}C NMR of 12

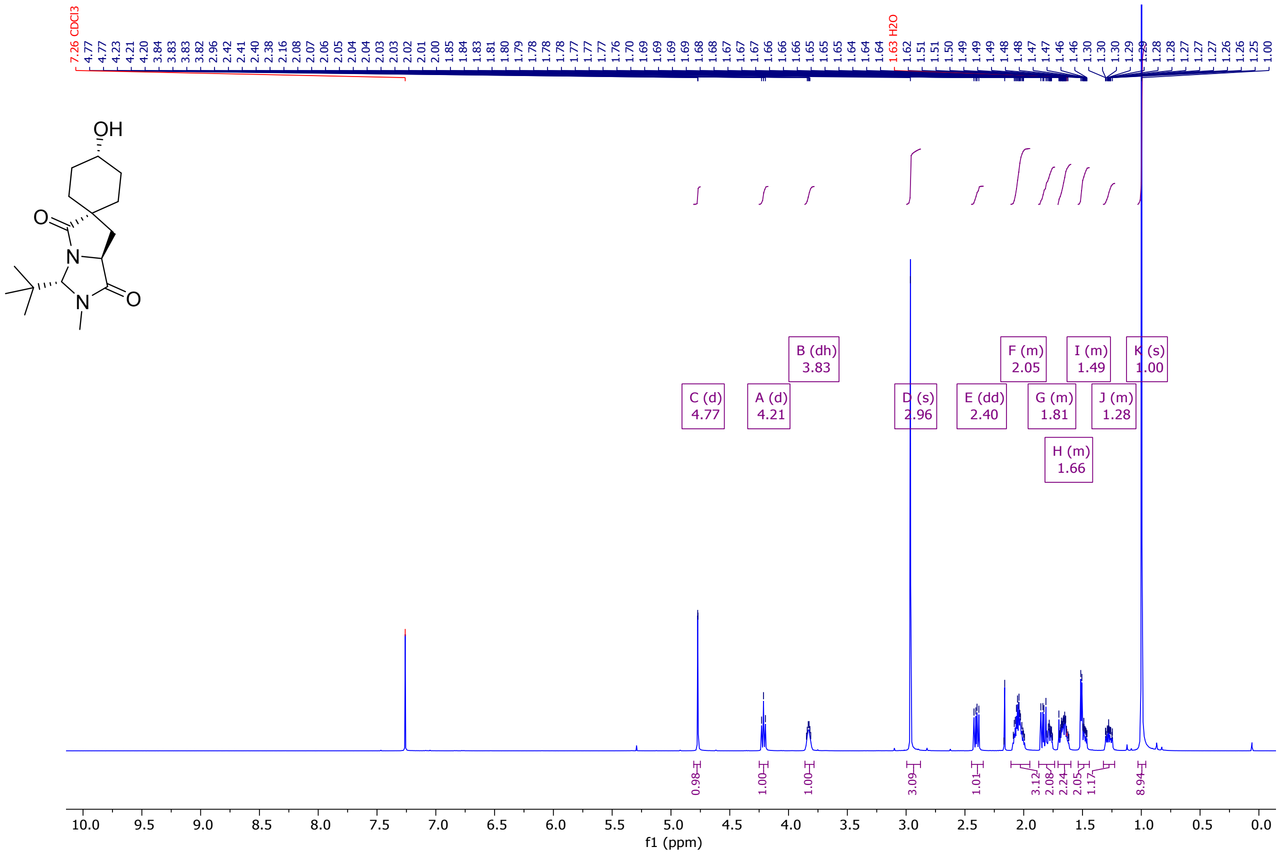

^{1}H NMR of 13a

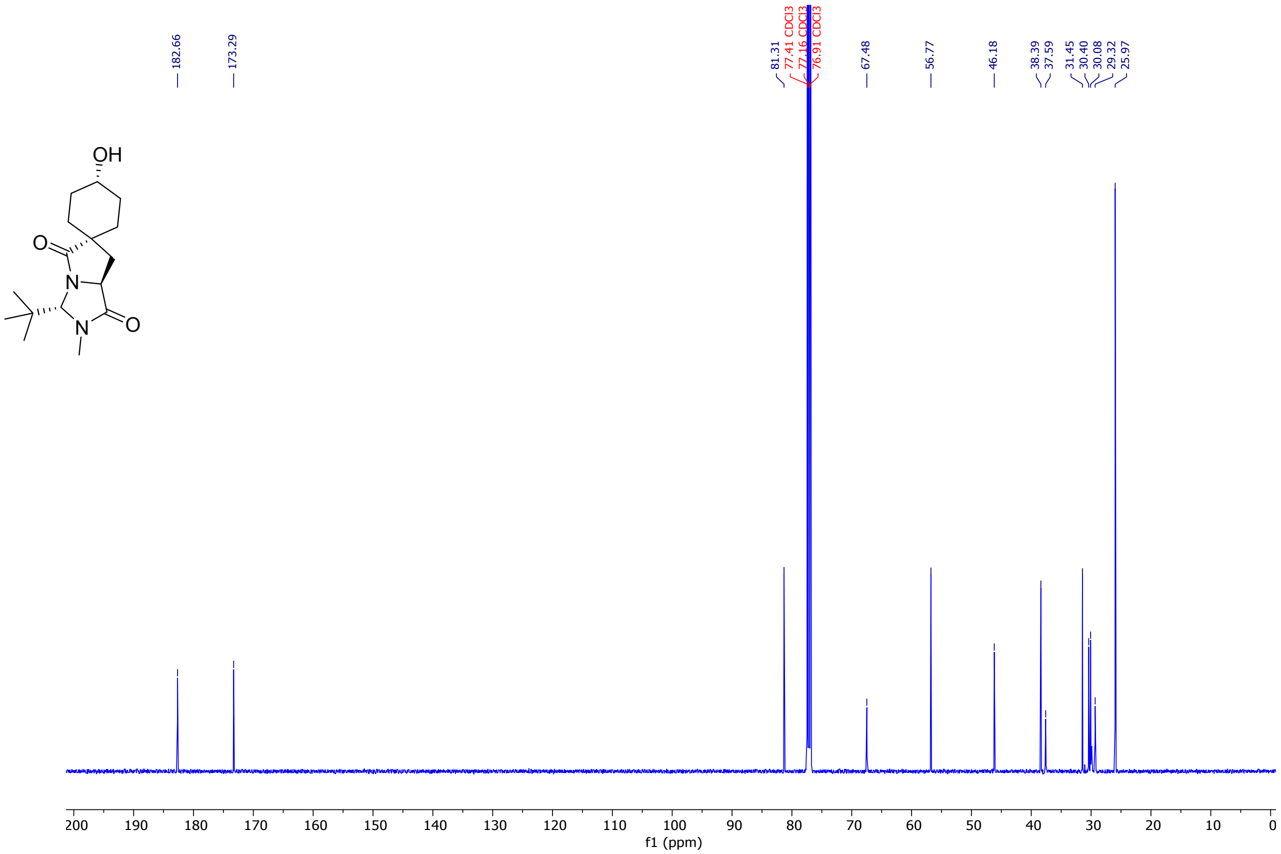

^{13}C NMR of 13a

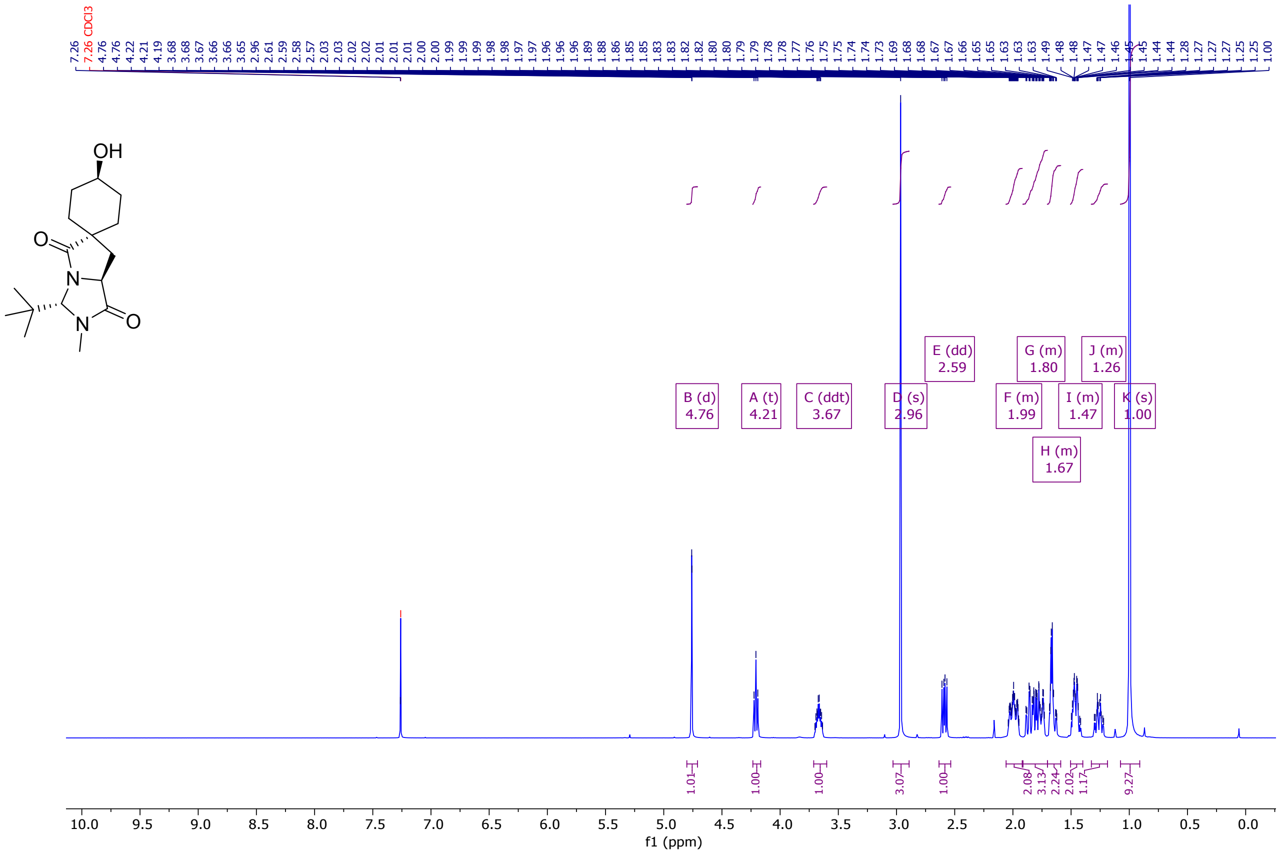

^{1}H NMR of 13b

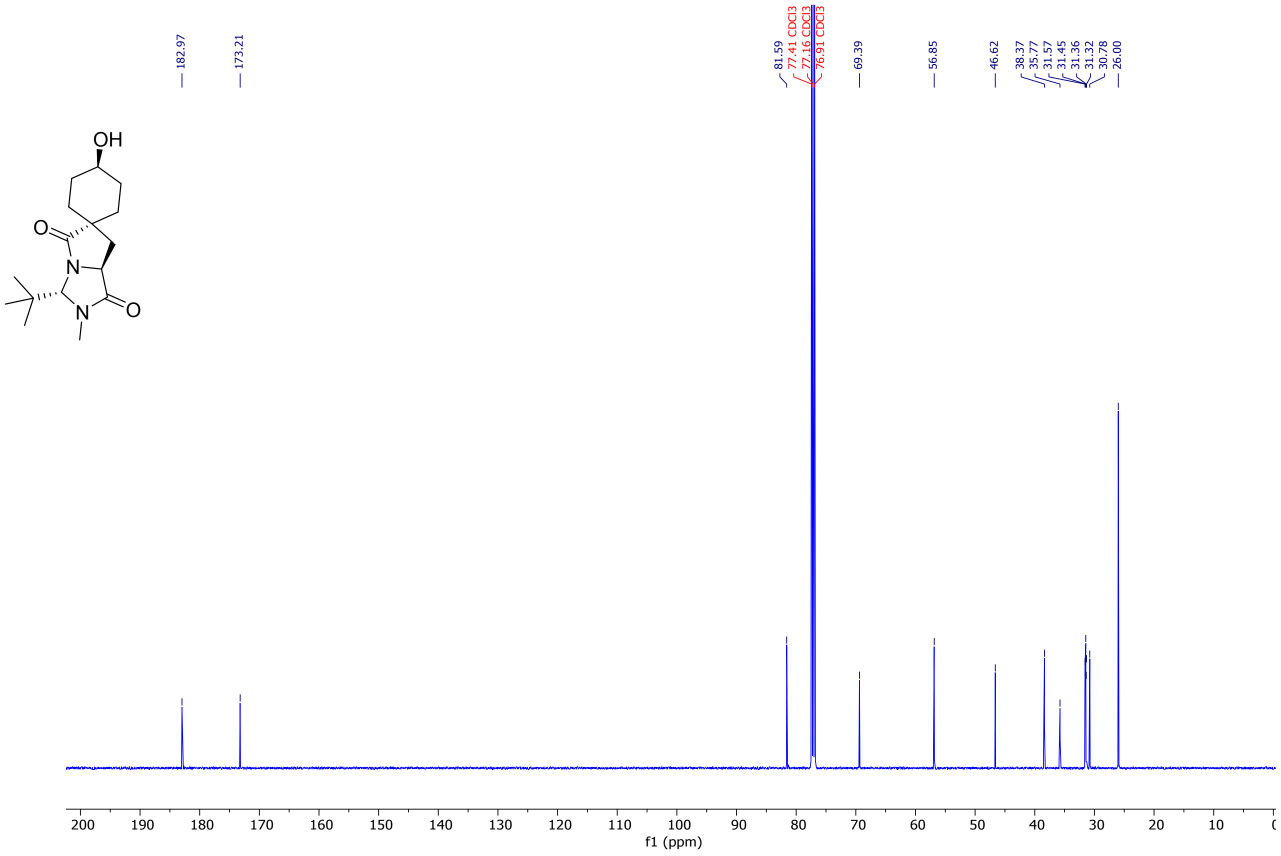

^{13}C NMR of 13b

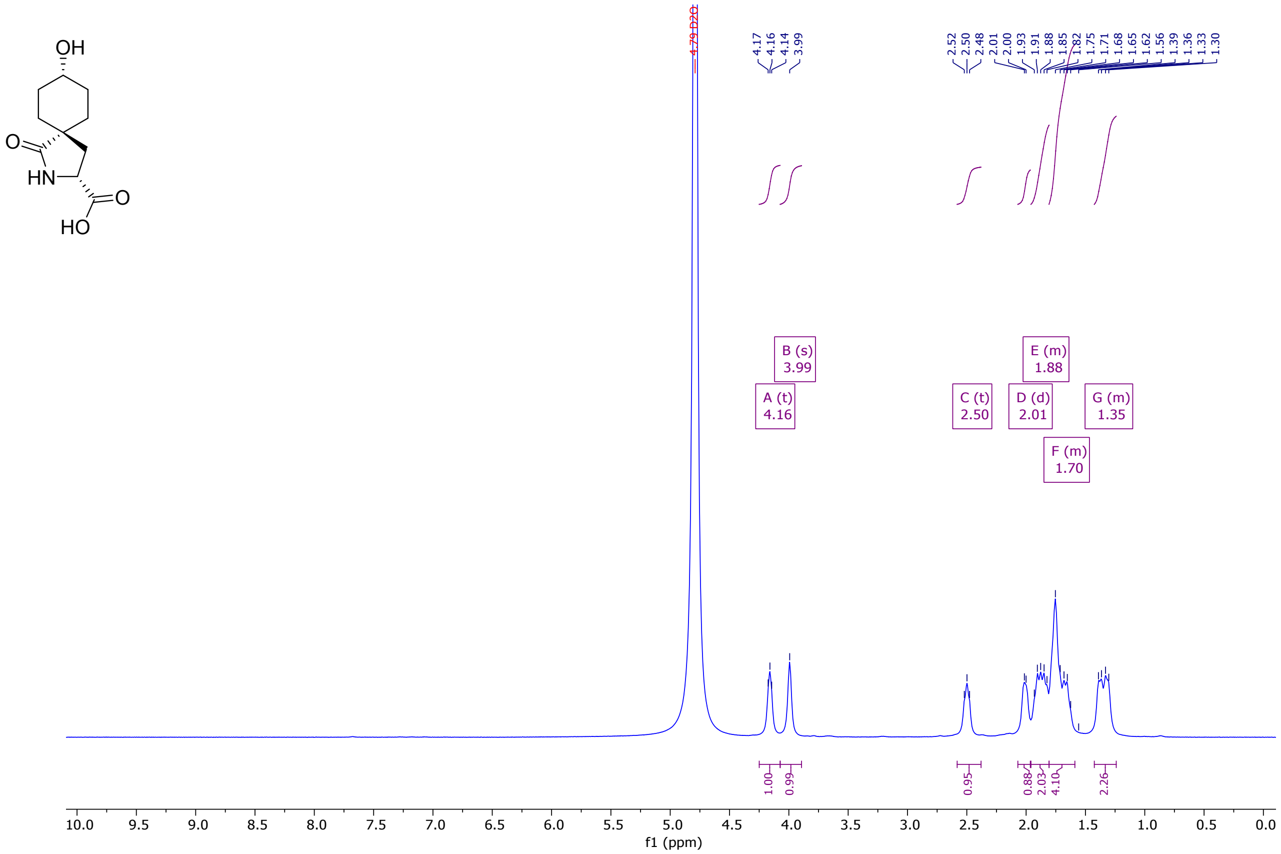

^{1}H NMR of 14

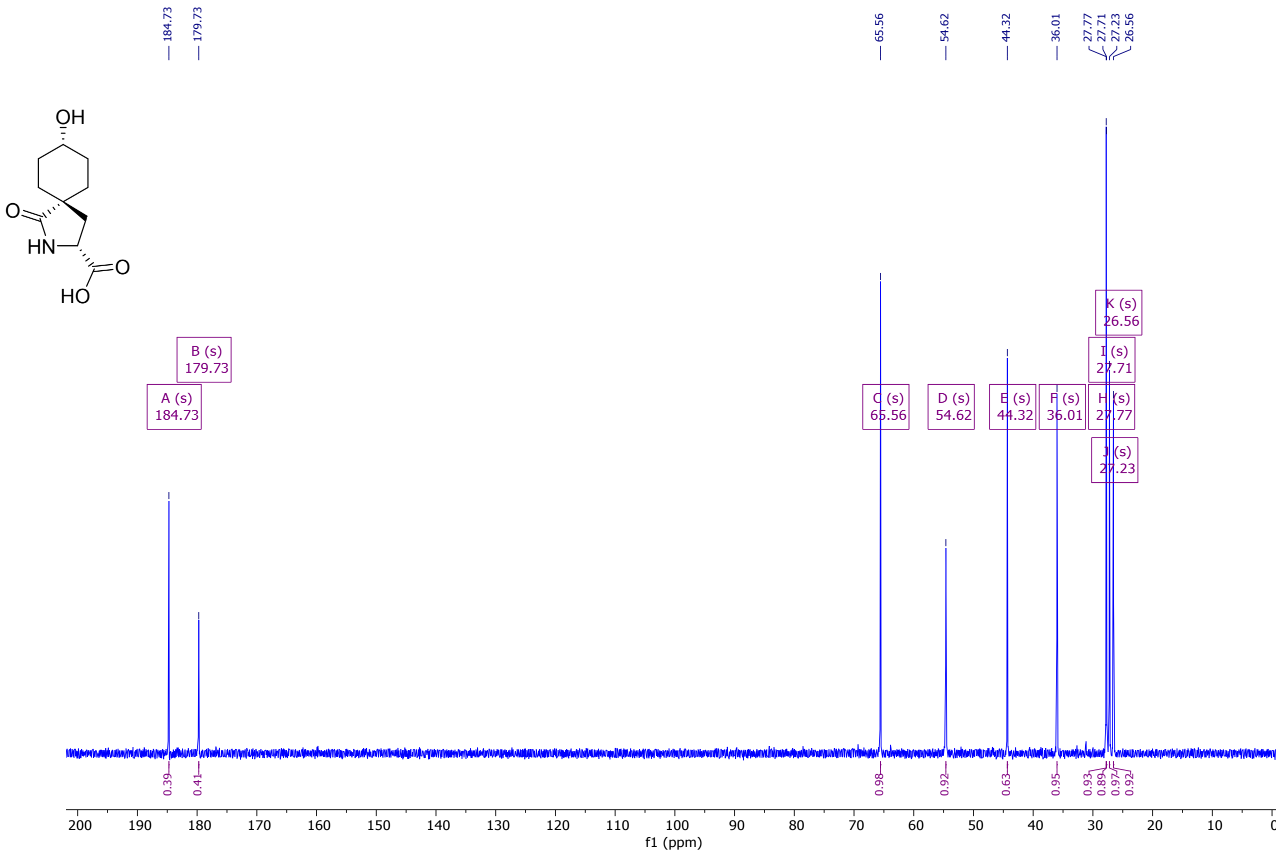

^{13}C NMR of 14

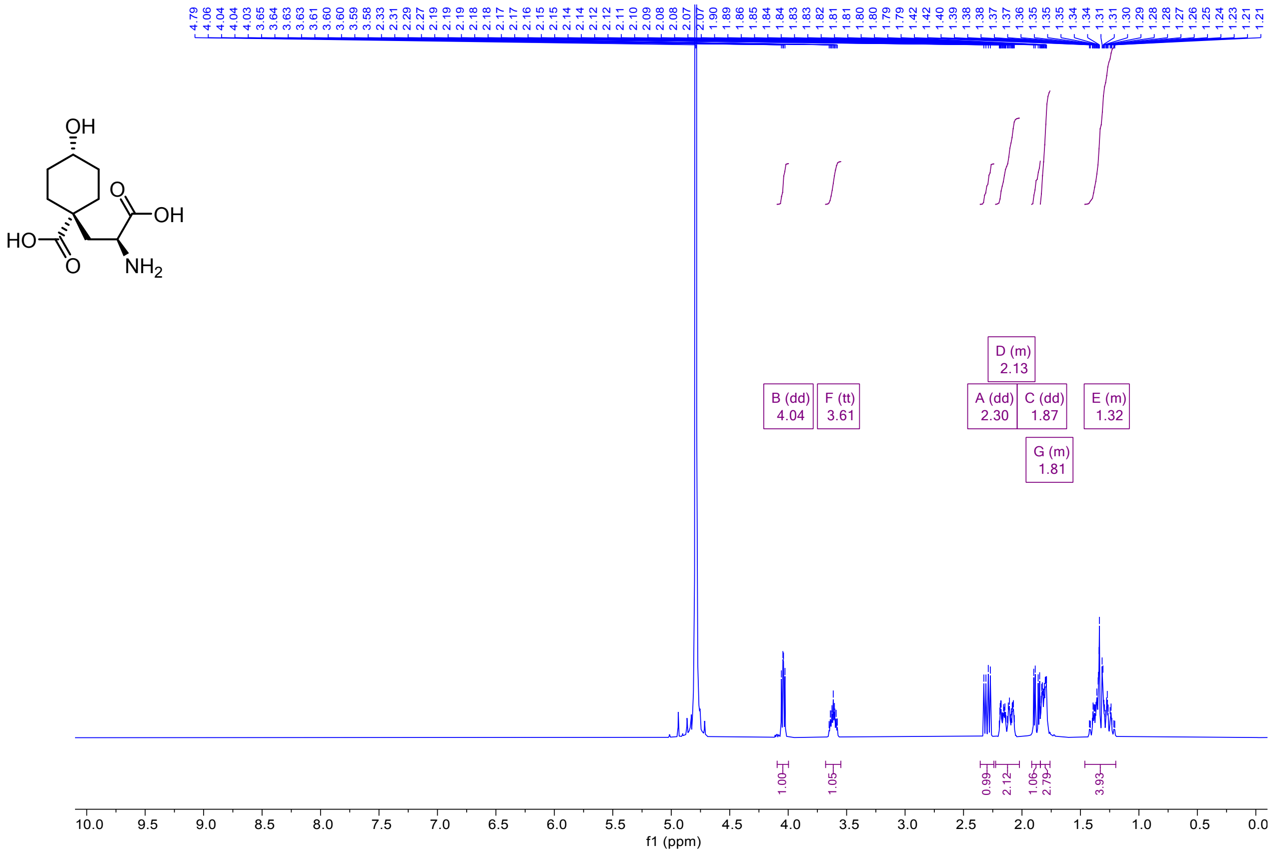

^{1}H NMR of 15

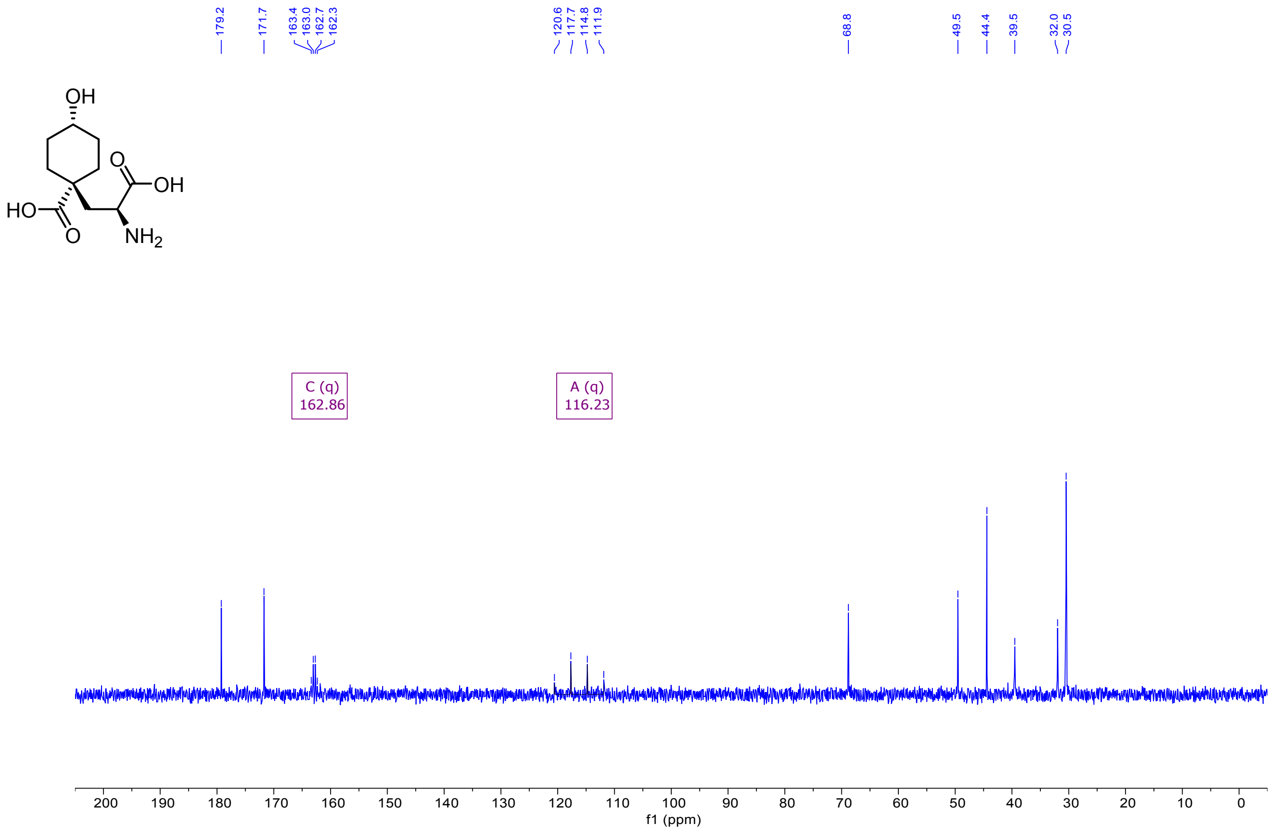

^{13}C NMR of 15

5. CD data

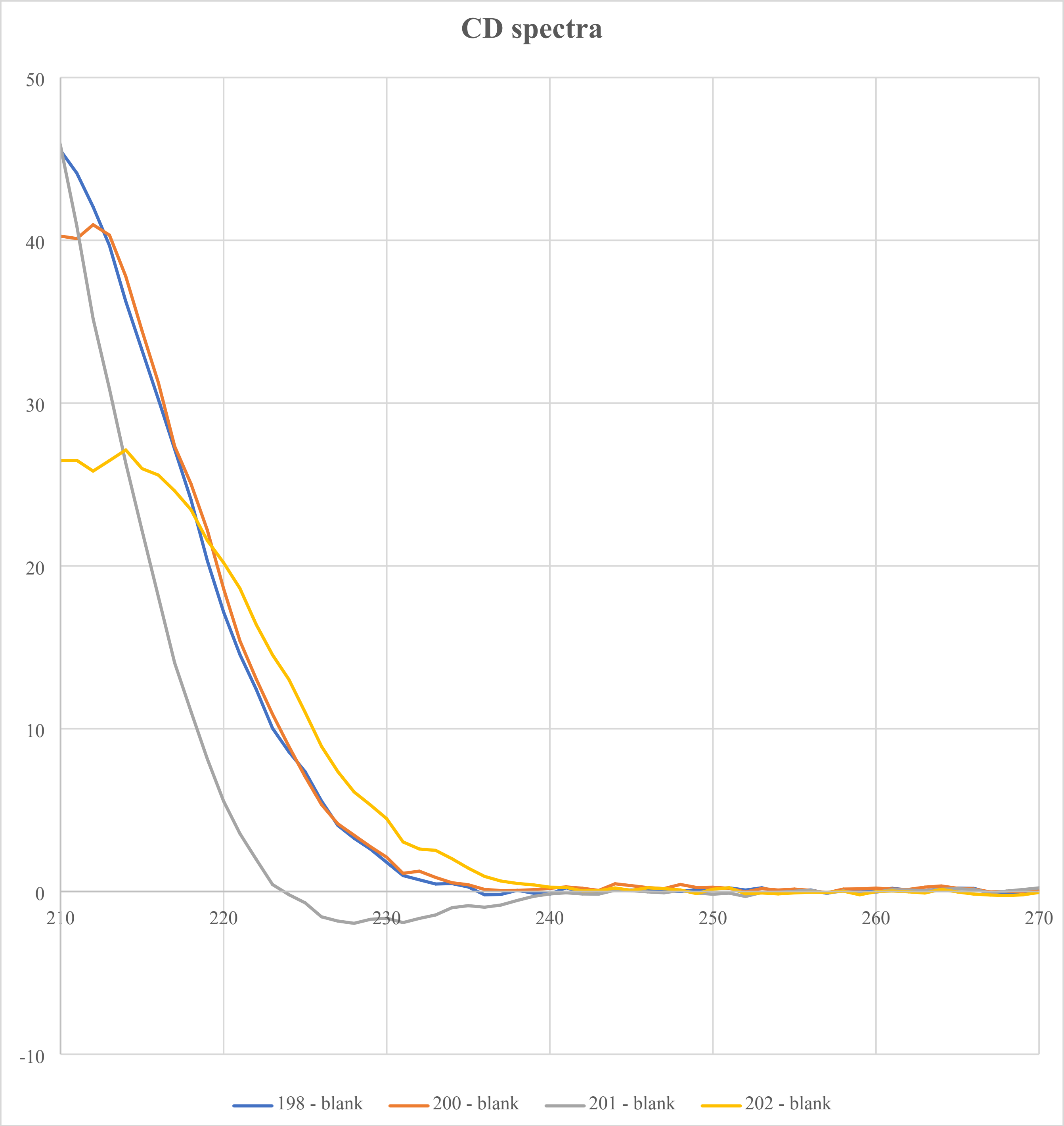

6. References

(1) Amer, M. M.; Carrasco, A. C.; Leonard, D. J.; Ward, J. W.; Clayden, J. *Org. Lett.* **2018**, *20*, 7977.

(2) Cerezo, V.; Amblard, M.; Martinez, J.; Verdié, P.; Planas, M.; Feliu, L. *Tetrahedron* **2008**, *64*, 10538.

(3) Knör, S.; Laufer, B.; Kessler, H. *J. Org. Chem.* **2006**, *71*, 5625.

(4) Fulmer, G. R.; M Miller, A. J.; Sherden, N. H.; Gottlieb, H. E.; Nudelman, A.; Stoltz, B. M.; Bercaw, J. E.; Goldberg, K. I.; Beckman, M. *Organometallics* **2010**, *29*, 2176.
